# Spatial-confinement induced electroreduction of CO and CO_2_ to diols on densely-arrayed Cu nanopyramids†

**DOI:** 10.1039/d1sc01694f

**Published:** 2021-05-07

**Authors:** Ling Chen, Cheng Tang, Kenneth Davey, Yao Zheng, Yan Jiao, Shi-Zhang Qiao

**Affiliations:** School of Chemical Engineering and Advanced Materials, The University of Adelaide South Australia 5005 Australia yan.jiao@adelaide.edu.au s.qiao@adelaide.edu.au

## Abstract

The electroreduction of carbon dioxide (CO_2_) and carbon monoxide (CO) to liquid alcohol is of significant research interest. This is because of a high mass-energy density, readiness for transportation and established utilization infrastructure. Current success is mainly around monohydric alcohols, such as methanol and ethanol. There exist few reports on converting CO_2_ or CO to higher-valued diols such as ethylene glycol (EG; (CH_2_OH)_2_). The challenge to producing diols lies in the requirement to retain two oxygen atoms in the compound. Here for the first time, we demonstrate that densely-arrayed Cu nanopyramids (Cu-DAN) are able to retain two oxygen atoms for hydroxyl formation. This results in selective electroreduction of CO_2_ or CO to diols. Density Functional Theory (DFT) computations highlight that the unique spatial-confinement induced by Cu-DAN is crucial to selectively generating EG through a new reaction pathway. This structure promotes C–C coupling with a decreased reaction barrier. Following C–C coupling the structure facilitates EG production by (1) retaining oxygen and promoting the *COH–CHO pathway, which is a newly identified pathway toward ethylene glycol production; and, (2) suppressing the carbon–oxygen bond breaking in intermediate *CH_2_OH–CH_2_O and boosting hydrogenation to EG. Our findings will be of immediate interest to researchers in the design of highly active and selective CO_2_ and CO electroreduction to diols.

## Abstract 2

Densely-arrayed Cu nanopyramids have spatial confinement induced by the additional Cu–O bond. This promotes C–C coupling, regulates post-C–C coupling, and retains both oxygen atoms in an alternative pathway toward ethylene glycol formation from CO.

## Graphical abstract

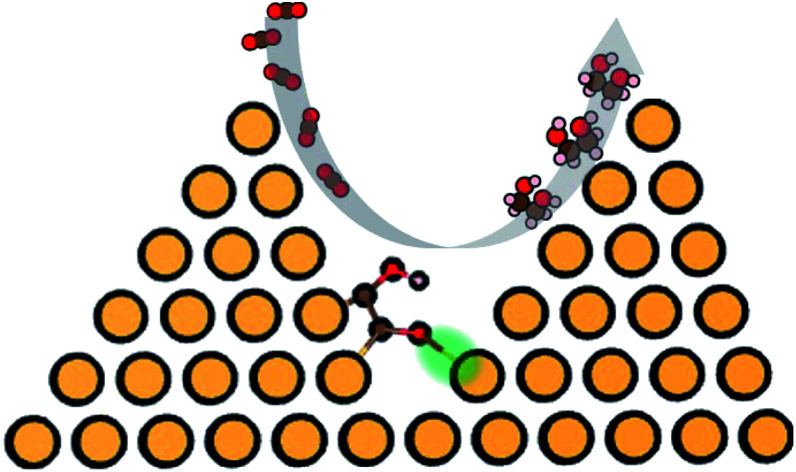

## Introduction

Electrocatalytic conversion of carbon dioxide (CO_2_) and carbon monoxide (CO) to value-added chemicals and fuels provides a sustainable and carbon-neutral route for storage of renewable energy.^1^ Electrocatalytic reduction of CO_2_ to C_1_ chemicals, including CO and formate (HCOO^−^), has been reported with relatively high efficiencies and reaction rates.^2,3^ Additionally, production activities and efficiencies toward particular C_2_ chemicals, including ethylene and ethanol, are improving.^4,5^ However, the selective production of higher-value C_2_ chemicals by electrochemical methods, such as diols, has not been demonstrated.

Diols are critical intermediates in synthetic chemistry and essential building blocks in organic chemistry. It is both practically important and fundamentally significant to discover new electrochemical production routes toward diols.^6,7^ For example, ethylene glycol ((CH_2_OH)_2_, EG) is the most common industrial diol.^8^ However, current mainstream technology for large-scale production of ethylene glycol relies on a multi-staged process that is energy and cost intensive, and is not economically sustainable.^8–12^ Alternatively, EG production from CO/CO_2_*via* electrochemical methods, especially when combining with renewable energy input, could offer an alternative route that is clean and sustainable.

A bottleneck for realizing conversion to diols is the development of necessary highly selective and active catalyst materials. This is practically challenging given that electrochemical reduction of CO_2_ or CO to alcohols has lower Faraday efficiency compared with hydrocarbon products, mainly because of the difficulty to retain oxygen for hydroxyl group formation.^13–15^ Several strategies have been proposed to improve selectivity of catalyst materials toward alcohol products. These strategies include morphology control,^16,17^ crystal phase engineering,^18^ bimetallic catalysts,^19^ molecule modification,^20^ vacancy engineering^21^ and compressive strain.^22^ In addition to these catalyst design strategies we have proposed a new strategy that could improve multi-carbon products selectivity, namely, formation of copper nanopyramids.^23^

These copper nanopyramids showed improved electrocatalytic activity that is attributed to a pyramidal effect that promotes C_2_ selectivity from three aspects: (1) improved *CO adsorption; (2) geometrically preferable sites for C–C coupling; and, (3) boosted surface electron transfer.^23^ In addition, we observed a unique feature which is the confined space between adjacent nanopyramids. We concluded that this might provide opportunities for C–C coupling and retaining of oxygen through O–Cu bond formation between the oxygen in the intermediates and copper on adjacent nanopyramids. Therefore we proposed nanopyramid-based structures to improve selectivity toward diols.

Given that reduction of CO_2_ to CO is close to 100% Faraday efficiency^2,4^ and that *CO is the most important intermediate in CORR and CO_2_RR,^6^ investigation of CO_2_ reduction to ethylene glycol can be readily combined with CO reduction. Following two *CO adsorption being formed on the surface, two post-C–C coupling pathways exist on a conventional copper surface following formation of *COH–CO as is outlined in Scheme S1.†^24–26^ Following dehydroxylation at different stages, both pathways lead to the formation of *CH–COH where the pathway bifurcates into two sub-routes toward hydrocarbons (*i.e.* ethylene) formation or oxygenates (*i.e.* ethanol) formation. The ratio of produced ethylene and ethanol is about 5 : 1, together with negligible diol such as ethylene glycol.^24,27,28^ The fundamental reason for a low selectivity for ethylene glycol lies in the dehydroxylation at early stage in conventional pathways: intermediates *COH–CO and *COH–COH are dehydroxylated to *C–CO and *C–COH, respectively, which make the pathway to diols blocked.^24,27^ Therefore existing catalysts that follow conventional reaction pathways are unable to retain the oxygen atoms in the key reaction intermediates for diol production.

The challenge could be addressed by enabling an alternative pathway by altering the coordination environment and confined-space, as such an environment could facilitate carbon–oxygen bond retention. Therefore we proposed that Cu nanopyramids with confined space could enable this new pathway for diol production.

The nanomorphology of a confined space boosts the adsorption of particular intermediates to drive selectivity along a desired reaction pathway. This is well-established and widely applied in catalyst design to regulate product selectivity. For example, Yang *et al.* used spatial confinement to explain a selectivity shift from C_1_ to C_2_ (ref. 29) and, Zhuang *et al.* applied it to boost C_3_ production through extending the retention of C_2_ species within copper nanocavity structures.^30^

**Fig. 1:**
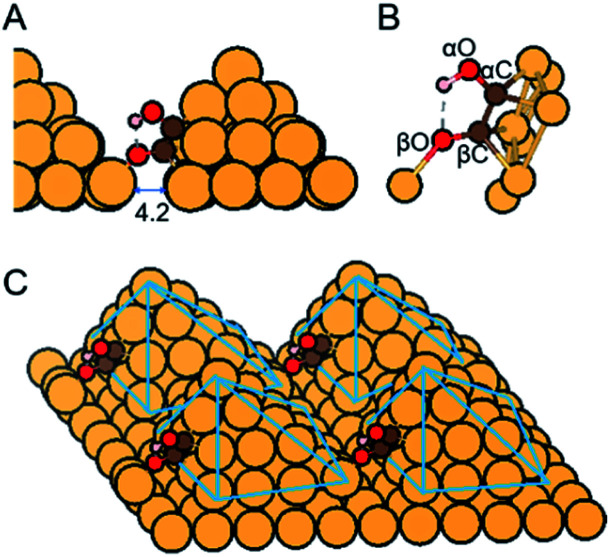
(A and B) Close-up and (C) overall view of atomic structure of *COH–CO reaction intermediate on Cu-DAN with one O atom binding with the adjacent nanopyramid. Distance shown is in Å. Color code: Cu, orange; C, brown; O, red; H, pink. Solid-blue lines are visual guides. *COH–CO with this atomistic arrangement has two C atoms bonded with each other; one of the carbon atoms connecting with a OH moiety, and the other with an oxygen atom. We refer to the C atom binding with OH moiety as αC, the O atom binding to αC as αO, and the rest C and O atoms as βC and βO, namely *(αC)(αO)H–(βC)(βO).

Here we report assessment of alternative pathways for the direct electroreduction of CO/CO_2_ to ethylene glycol on densely arrayed Cu nanopyramids (Cu-DAN) using density functional theory (DFT) computations. Our results show that a unique spatial-atomistic arrangement on Cu-DAN promotes C–C coupling and accelerates the formation of favorable intermediates following C–C coupling. Because of the confined space there is an extra bond between the adsorbed *COH–CO and the adjacent Cu nanopyramid as is shown in Fig. 1. This Cu–O interaction keeps the C–O bond intact against dehydroxylation, alters the objective and priority of hydrogenation to form intermediates with comparatively lower kinetic formation energy, and increases the kinetic barriers for competing pathways. This facilitates an alternative *COH–CHO reaction pathway that leads to direct electrosynthesis of ethylene glycol from CO.

## Methods and models

### Models

We examined three surfaces and compared the relevant ethylene glycol production pathways. The three structures include Cu-DAN, sparsely arrayed Cu nanopyramid (Cu-SAN), and planar Cu(100). These latter two are included for comparison. The lattice constant for Cu was optimized to be 3.64 Å in its fcc crystal structure. As is displayed in Fig. S1† four [111] diamond nanopyramids^23^ were proposed based on a 10 × 10 × 1 Cu surface with 4.2 Å distance in between to represent the dense-array, and on a 12 × 12 × 1 Cu surface with 5.8 Å distance to represent the sparse-array. The base layers were fixed whilst nanopyramids and adsorbates were permitted to fully relax in all configurations. Planar Cu(100) was modeled with a periodic 4 × 3 × 3 model with two bottom layers fixed and top layer relaxed. The vertical separation between periodically repeated images was set at least 10 Å in all cases to ensure no interaction between images. Moreover, the proposed Cu-DAN models show electrochemical stability as analyzed in ESI Fig. S2 and Note 1.†

### Computational methods

DFT computations were performed with the Vienna *Ab Initio* Simulation Package (VASP) code.^31^ The Perdew–Burke–Ernzerhof (PBE) functional was employed to compute the electron exchange–correlation energy.^32^ Projector Augmented Wave (PAW) potentials were used to describe the ionic cores.^33^ The atomic relaxations were carried out with the quasi-Newton minimization scheme, until the maximum force on any atom was less than 0.02 eV Å^−1^. Geometry optimizations were performed with a plane-wave cutoff of 400 eV. An irreducible 3 × 3 × 1 Monkhorst–Pack *k*-point grid was used^34^ with the center shifted to the gamma point. The Fermi level was smeared with the Methfessel–Paxton approach with a smearing of 0.1 eV. Dipole corrections were included in all computations to minimize inaccuracies in the total energy because of simulated slab interactions. The dipole moment was computed parallel to the *z*-direction.

A general electroreduction reaction is described by the following eqn (1):

1 *A + H^+^ + e^−^ = *AH

in which the asterisk denotes surface bound species. Potential-dependent free energy change Δ*G* can be determined by the linear free energy method under computational hydrogen electrode assumption as is shown in eqn (2):^33,34^

2 Δ*G* = G_*AH_ − G_*A_ − [*G*_H_2__/2 − *eU*]

where *G* denotes free energy for different state. To determine the free energy at room temperature (300 K), Zero Point Energy (ZPE), heat capacity and entropy were computed with standard methods.^35,36^ The free energy of relevant gas molecules are given in Table S1.†

Kinetic barrier is essential to determine the product selectivity and dominant reaction pathway. This was computed using the nudged elastic band (NEB) method.^37^ The total energy and force thresholds for geometry optimizations were 1 × 10^−5^ eV and 0.05 eV Å^−1^, respectively. The minimum energy pathway (MEP) was examined using six images during the transition state search.

We identified in total 10 relevant elementary reaction steps, and correspondingly calculated 13 possible pathways on three surfaces, as is summarized in Table S2.† Both H-shuttling and surface *H transfer mechanism were considered for these reactions. Solvation effects were considered under H-shuttling model by including one explicit water molecule in the computations.^36,38^ Such consideration for solvation effect was used previously to study facet dependence of CO_2_ reduction pathways on Cu surface.^39^ The MEP for these 13 pathways are presented in ESI Fig. S3–S12,† with the atomic coordinate of initial, transition and final state summarized in Tables S3–S15.† Each transition state was confirmed to have a single imaginary vibrational frequency along the reaction coordinate. The kinetic barriers Δ*G*^‡^ under potential *U* were then deduced from MEP obtained Δ*G*^‡^(*U*^0^) as is described in eqn (3).^39^

3 Δ*G*^‡^(*U*) = Δ*G*^‡^(*U*^0^) + *eβ′*(*U* − *U*^0^)

where *U*^0^ is the equilibrium potential for the reductive adsorption of one proton in the system, and *β′* is the reaction symmetry factor. The value of *β′* is 0.49 approximated from the average value for 36 CO_2_ Reduction Reaction (CRR) on copper surface (median = 0.49, standard deviation = 0.04).^28^ All Δ*G*^‡^ reported in this manuscript without specification of potential are under 0 V *vs.* RHE.

In computing relative onset potential we assumed that 0.75 eV is the threshold barrier (*E*^threshold^_a_) that can be overcome to give acceptable production rates at room temperature.^40^ For key reaction steps identified in the alternative pathway, a lower *E*^threshold^_a_ of 0.40 eV was adopted.^41^ The onset potential *U*_onset_ was computed *via* solving the expression of potential-determining step (PDS), eqn (4).

4 *E*^threshold^_a_ = Δ*G*^‡^(*U*^0^) + *eβ′*(*U*_onset_ − *U*^0^)

## Results and discussion

**Fig. 2:**
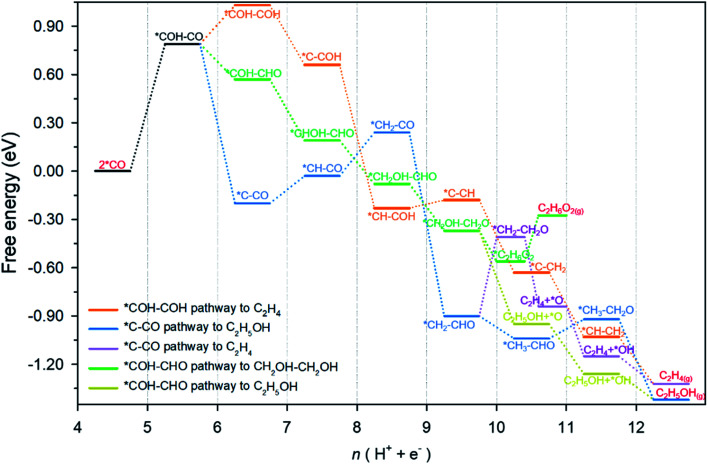
Reaction pathways and free energy for each state identified on Cu-DAN. The reaction pathway from CO_2_ or CO to *CO adsorption is not shown. The reference energy level is set to be two *CO adsorption at 0 V *vs.* reversible hydrogen electrode (RHE). Pathways toward C_2_ products (ethylene, ethanol and ethylene glycol) beyond 2 *CO are shown as different colored branches: orange, *COH–COH pathway toward C_2_H_4_, blue, *C–CO pathway toward C_2_H_5_OH, purple, *C–CO pathway bifurcating to C_2_H_4_, green, *COH–CHO pathway toward C_2_H_6_O_2_ and, brown, *COH–CHO pathway bifurcating to C_2_H_5_OH.

An overview of possible reaction pathways and related free energy values identified in this research is given in Fig. 2. Pathways demonstrated in the figure begin with 2 *CO on Cu-DAN. Further reduction of 2 *CO to C_2_ products proceeds in three major pathways, namely, (1) *COH–COH pathway (in orange color) – a conventional pathway toward ethylene (C_2_H_4_) formation;^24^ (2) *C–CO pathway which bifurcates from *CH_2_–CHO to ethylene (in purple) and ethanol (C_2_H_5_OH, blue);^25,26^ and, (3) *COH–CHO pathway for CH_2_OH–CH_2_OH formation (green) with a bifurcation to C_2_H_5_OH (brown) from *CH_2_OH–CH_2_O. Fig. S13† illustrates the chemical structure of all intermediates in the overall reaction network. The competing hydrogen evolution reaction (HER) is significantly inhibited on the structure, as is shown in ESI Fig. S14 and Note 2.† In the following we explore the reduction of *CO moieties on Cu-DAN surface in the major pathways, and provide quantitative insight into how the morphology drives selectivity for ethylene glycol over alternative C_2_ products, including ethylene/ethanol.

### CO–CO coupling to *COH–CO

The coupling of two *CO, and subsequent proton-coupled electron transfer (PCET, 2 *CO + H^+^ + e^−^ → *COH–CO), occur concomitantly. This was identified as the rate-determining step (RDS) for the onset potential.^22^ We referenced published data as the benchmark to decide whether C–C coupling was kinetically facile. Luo *et al.* for example, adopted this same computational model to determine the reaction barrier for C–C coupling on planar Cu(100) as equal to 1.52 eV.^39^ In consequence for a reaction barrier value less than 1.52 eV we reasonably assumed the C–C coupling is kinetically facile. This is because the coupling performance is better than planar Cu(100) surface known to favor C_2_ production, and *vice versa*. We calculated the C–C coupling steps from two models, namely the Cu-SAN and Cu-DAN, to reveal the coupling promotion supplied by confined-space. Additionally, *CO coverage has some impact on C–C coupling. This is analyzed in ESI Fig. S15, S16 and Note 3.†

**Fig. 3:**
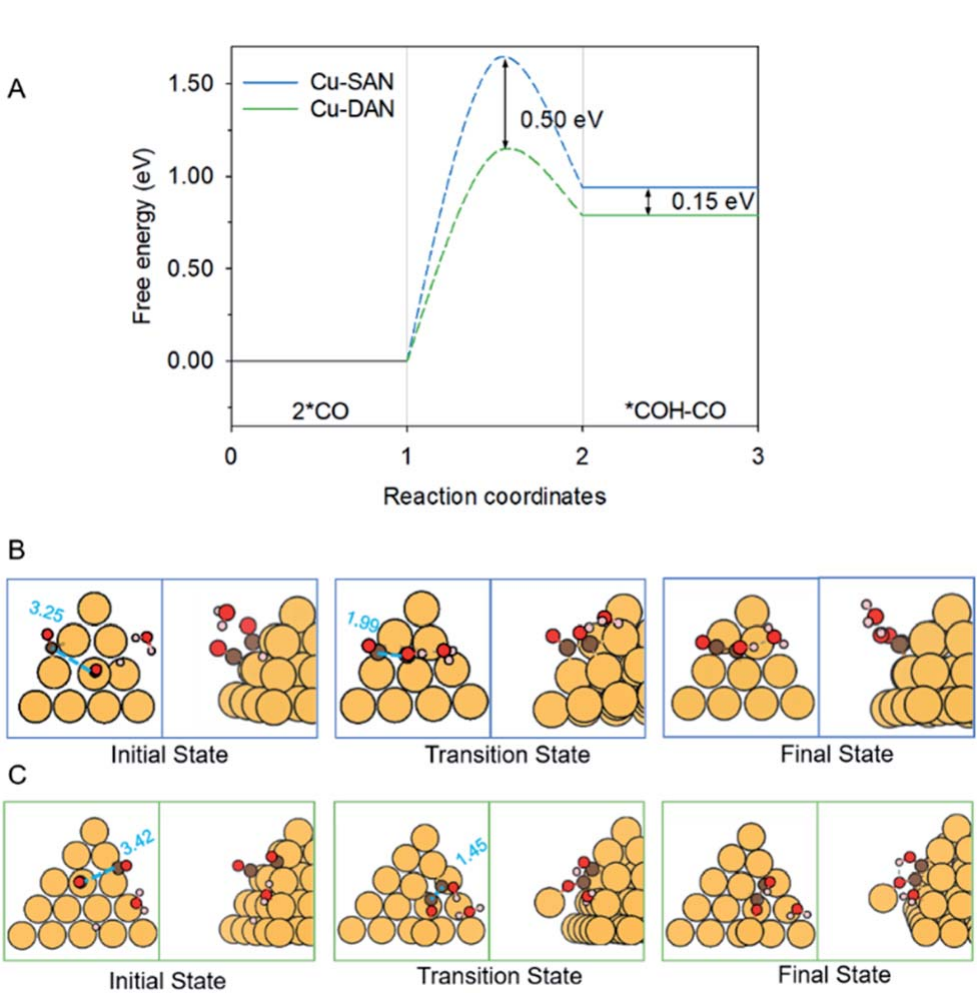
(A) Reaction energetics involved in 2 *CO dimerization and reduction to *COH–CO at 0 V *vs.* RHE. Structures of the initial, transition and final states in CO–CO coupling on (B) Cu-SAN and (C) Cu-DAN, respectively. Minimum energy path for reaction barriers in panel A is found in Fig. S3.† Color code: Cu, orange; C, brown; O, red; H, pink. Distances are in Å.

The reaction pathways and different transition state structures for *COH–CO formation on Cu-SAN and Cu-DAN are displayed as Fig. 3. As is shown in Fig. 3A *COH–CO formed on Cu-DAN establishes an extra O–Cu bond with the adjacent Cu nanopyramid. This state is more stable by 0.15 eV compared with that for Cu-SAN. The coupling barrier on Cu-DAN of 1.13 eV is significantly less than that of the adopted benchmark of 1.52 eV. This barrier value on Cu-DAN is 0.50 eV less than that on Cu-SAN of 1.63 eV. The lowered barrier significantly decreases the onset potential from −1.78 to −0.77 V *vs.* RHE, which means that the C–C coupling on Cu-DAN is significantly promoted. We conclude that this finding is a result of the combined effect of the breaking of scaling relation between key intermediates, together with more active C–C bond formation facilitated by spatial-confinement.

### Spatial-confinement facilitated *COH–CO formation

The binding energies of reaction intermediates on catalysts are dependent on each other, as is decided by scaling relationships.^42,43^ This is because adsorptions of different intermediates with the same pattern, *e.g.* C containing intermediates are correlated.^44,45^ Although this intrinsic relationship facilitates activity prediction of heterogeneous catalysts, it always imposes limitations to improve catalytic performance as optimizing for one adsorbed species will reduce other steps from optimal value. This underscores that establishing effective strategies to break the scaling relationships for optimized CRR performance is a present significant research theme.^46,47^

As is displayed in Fig. S17,† the adsorption energy of 2 *CO on Cu-SAN is linearly-correlated to the formation energy of *COH–CO with a slope of −0.37. However the formation energy of *COH–CO on Cu-DAN is significantly shifted away from the scaling relation, with the value reduced from 0.51 eV (the trend fitted level) to 0.18 eV. This finding indicates that the extra O–Cu bond formed between βO of *COH–CO and Cu atom of the adjacent nanopyramid breaks the scaling relation between carbonaceous intermediates, *CO and *COH–CO. The reason is that the new adsorption pattern (*via* O adsorption) introduced by forming extra O–Cu bond independently boosts the adsorption of *COH–CO, whilst the adsorption of 2 *CO in the previous step is almost unaffected because the electronic properties of surface Cu is unchanged. In consequence *CO dimerization is promoted on the densely-arrayed nanopyramids. This is in contrast to other approaches such as elemental alloying^48^ and vacancy engineering.^21^

We attribute the underlying reason for the breaking of scaling relationship to the spatial-confinement effect on Cu-DAN. As is shown in Fig. 3B, on Cu-SAN the distance between two *CO moieties in the initial state is 3.25 Å. In the transition state these two C atoms have not coupled, although the distance is decreased to 1.99 Å. The reaction barrier for this step of 1.63 eV at 0 V *vs.* RHE is greater than that of the benchmark. This means C–C coupling is not facile on this surface. As is shown schematically in Fig. 3C on Cu-DAN, the distance between two *CO moieties is significantly shortened from 3.42 Å in the initial state to 1.45 Å – this being a typical C–C single bond length – in the transition state, with a lowered reaction barrier of 1.13 eV. Both O–Cu and C–C bond formation complete in the transition state on Cu-DAN. This confirms that this structure with an extra O–Cu bond provides a more active C–C coupling site *via* reducing the energy of the transition state. Because of the difficulty of C–C coupling on Cu-SAN, this structure was not further investigated.

### *COH–COH pathway

**Fig. 4:**
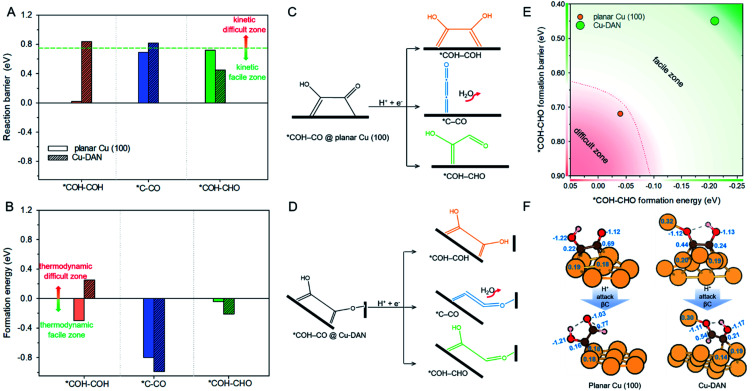
(A) Reaction barrier, (B) formation energy of key intermediates *COH–COH, *C–CO and *COH–CHO, and reaction schemes toward formation of intermediates on (C) planar Cu(100) surface, and (D) Cu-DAN. The reaction barriers for *COH–COH and *C–CO on planar Cu(100) surface were obtained from Cheng *et al.*^24^ The energy bars for *COH–COH, *C–CO and *COH–CHO are displayed in, respectively, orange color, blue and green, to coordinate with the code in Fig. 2. (E) Reaction energetics involved in *COH–CO reduction to *COH–CHO on planar Cu(100) surface and Cu-DAN with extra O–Cu bond. (F) Adsorption geometries of *COH–CO and *COH–CHO on, respectively, planar Cu(100) and Cu-DAN. The Bader charges (|*e*^−^|) on *COH–CO and *CHO–COH are marked on intermediates and copper atoms as active center. Color code: Cu, orange; C, brown; O, red; H, pink.

Following the formation of *COH–CO, the pathway trifurcates to *COH–COH, *C–CO and *COH–CHO. The reaction barrier and formation energy of the three key intermediates on all investigated surfaces are summarized in Fig. 4, together with the formation scheme on Cu(100) and Cu-DAN.

On Cu-DAN, the *COH–COH pathway starts with cleavage of strong O–Cu bond (2.63 eV, Fig. S18†) and successive protonation of O (MEP in Fig. S4†). This bond breaking imposes a higher barrier toward *COH–COH formation on Cu-DAN. This is 0.84 eV at 0 V *vs.* RHE, in comparison with 0.02 eV at 0 V *vs.* RHE on the Cu(100) surface.^24^ In addition, this process on Cu-DAN is endergonic with an uphill free energy change of 0.25 eV. Therefore the pathway is both thermodynamically and kinetically unfavored on Cu-DAN, compared with other competitive pathways.

### *C–CO pathway

On both Cu(100) surface and Cu-DAN, *C–CO formation is thermodynamically advantageous whilst kinetically disadvantageous (Fig. 4A and B).^24^ The barrier value for *COH–CO reduction to *C–CO on planar Cu(100) is high (Δ*G*^‡^ = 0.69 eV),^24^ and is attributed to the significant atomic configurations transformation from a parallel adsorption pattern with two C atoms binding to surface in the initial state, to a vertical adsorption pattern with only one bond formed between copper and carbon atom in the final state (Fig. 4C).^26^ With Cu-DAN, *COH–CO is dehydrated to *C–CO with a downhill Δ*G* = −0.99 eV and reaction barrier Δ*G*^‡^ = 0.82 eV (MEP in Fig. S5A†). This increased reaction barrier on Cu-DAN surface is attributed to the spatial-confinement effect (Fig. 4D and S19†). In the final state, three atoms in the *C–CO adsorbate align linearly, as is the same as that on Cu(100). However, the O–Cu bond between βO and Cu atom of the adjacent nanopyramid limits the stretching of βO atom, and therefore a higher reaction barrier. It is concluded therefore that on Cu-DAN surface the *C–CO pathway is less probable.

### *COH–CHO pathway

**Scheme 1:**
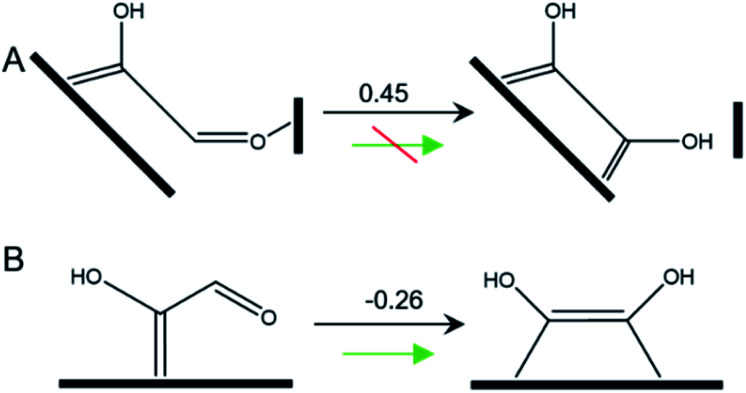
Tautomerization of *COH–CHO on (A) Cu-DAN surface and (B) planar Cu(100). The values shown above arrows are Δ*G* (eV) at 0 V *vs.* RHE.

In this pathway, βC on *COH–CO is protonated to form *COH–CHO. The free energy change Δ*G* is −0.21 eV for *COH–CHO formation with a low energy barrier Δ*G*^‡^ = 0.45 eV (Fig. S6A†). This barrier value is lower than that on Cu(100) (Fig. S6B†), and is contributed to two factors: (1) the extra βO–Cu bond impedes cleavage of O–Cu bond and the subsequent H atom shift from βC to βO toward *COH–COH (Scheme 1A). This significantly facilitates and stabilizes formation of *COH–CHO which is thermodynamically more stable than *COH–COH by 0.45 eV, and; (2) the (100) and (111) facets possessed by Cu-DAN facilitate *CHO and *COH adsorption, and correspondingly therefore promote coupling between *COH and *CHO.^37^ Consequently the formation of *COH–CHO has significantly lower formation energy and reaction barrier in comparison with planar Cu(100) surface. This is shown in Fig. 4E.

On Cu(100) such a pathway is unfavored due to the high energy barrier. In addition *COH–CHO can undergo a facile tautomerization to *COH=COH (Scheme 1B and ESI Note 4†). Therefore this new pathway is considered as exclusive to Cu-DAN.

### Origin of *COH–CHO formation on Cu-DAN

We performed further analyses to reveal how the extra O–Cu bond on Cu-DAN facilitates formation of *COH–CHO. Fig. 4F shows the adsorption geometries of the reactant and product regarding *COH–CHO formation (*COH–CO + H^+^ + e^−^ → *COH–CHO) on planar Cu(100) and Cu-DAN and corresponding Bader charge analysis of the surface atoms.^49^

On planar Cu(100) surface the *COH–CO binds through two C atoms to four surface Cu atoms. The number of electrons provided by Cu bonded to C atoms is around 0.20 e^−^. On Cu-DAN surface the *COH–CO binds through both C and βO atoms to five surface Cu atoms. The number of electrons provided by Cu to C atoms is similar to that on planar Cu(100). However the electron provided by Cu bonded to βO is greater (0.32 e^−^). This finding suggests that some electron are transferred to adsorbate *via* the extra Cu–O bond. The electrons on βO are similar on both planar Cu(100) and Cu-DAN surfaces (1.12 e^−^). Therefore the extra electron transfer to βO from the adjacent nanopyramid on Cu-DAN indicates that βC provides less electron charge to βO. This results in more electrons kept on the βC atom than on planar Cu(100) (0.25 e^−^ more).

The βC atom is a type of Lewis base with a lone electron pair, whilst H^+^ is a naked proton with an unoccupied molecular orbital. The protonation of βC is the process where βC utilizes the highest occupied molecular orbital (HOMO) to interact with the lowest unoccupied molecular orbital (LUMO) of H^+^.^50^ A more negatively charged βC means a stronger Lewis base with a higher nucleophilicity, which facilitates protonation.

### Post *COH–CHO pathways on Cu-DAN and role of spatial-confinement

Following the formation of *COH–CHO, reaction steps thereafter exhibit a preference toward hydrogenation of C atoms to produce *CHOH–CHO, rather than dehydroxylation to *C–CHO. This is because both the reaction barrier and free energy change for *CHOH–CHO (0.56 eV and −0.38 eV, Fig. S7†) are lower than those for *C–CHO (1.07 eV and 0.04 eV, Fig. S8†). *CHOH–CHO is then reduced successively to *CH_2_OH–CHO with Δ*G*^‡^ = 0.23 eV and Δ*G* = −0.27 eV (Fig. S9†), and *CH_2_OH–CH_2_O with Δ*G*^‡^ = 0.43 eV and Δ*G* = −0.29 eV (Fig. S10†). Notably, all reaction barriers are readily surmountable at room temperature. Amongst these reduction steps, spatial-confinement shows a pivotal role to preferential hydrogenation of C atoms, whilst keeping both O atoms intact. This is the key requirement to produce diols. How spatial-confinement drives selectivity toward oxygenates through concomitantly protecting both O atoms against dehydroxylation is explained in the following.

Further hydrogenation of key intermediate *COH–CHO on the COH moiety has two options as is illustrated in Scheme S2:† (1) protonation of αO and subsequent dehydroxylation to *C–CHO, and; (2) direct protonation of αC to *CHOH–CHO. *C–CHO transformation is not facile to process on Cu-DAN. This is because of spatial-confinement induced relocation of adsorption site. With the cleavage of αC–OH bond as a consequence, αC forms a stronger bond with surface Cu with shorter bond length so as to conform to the octet rule (namely, that every carbon atom must form four covalent bonds to achieve a stable structure with eight electrons in the valence shell).^51^ The *C–CHO transformation is not facile to proceed on Cu-DAN because the spatial confinement restricts both sides of the intermediate from stretching, and consequently relocates αC to hexagonal close packed (HCP) site (Fig. S20†). In contrast the formation of *CHOH–CHO is more facile. This is because of less restriction from spatial-confinement, and results in a significantly lower formation barrier. Therefore the spatial-confinement promotes selective hydrogenation on αC to leave αO reserved in the hydroxyl group.

**Fig. 5:**
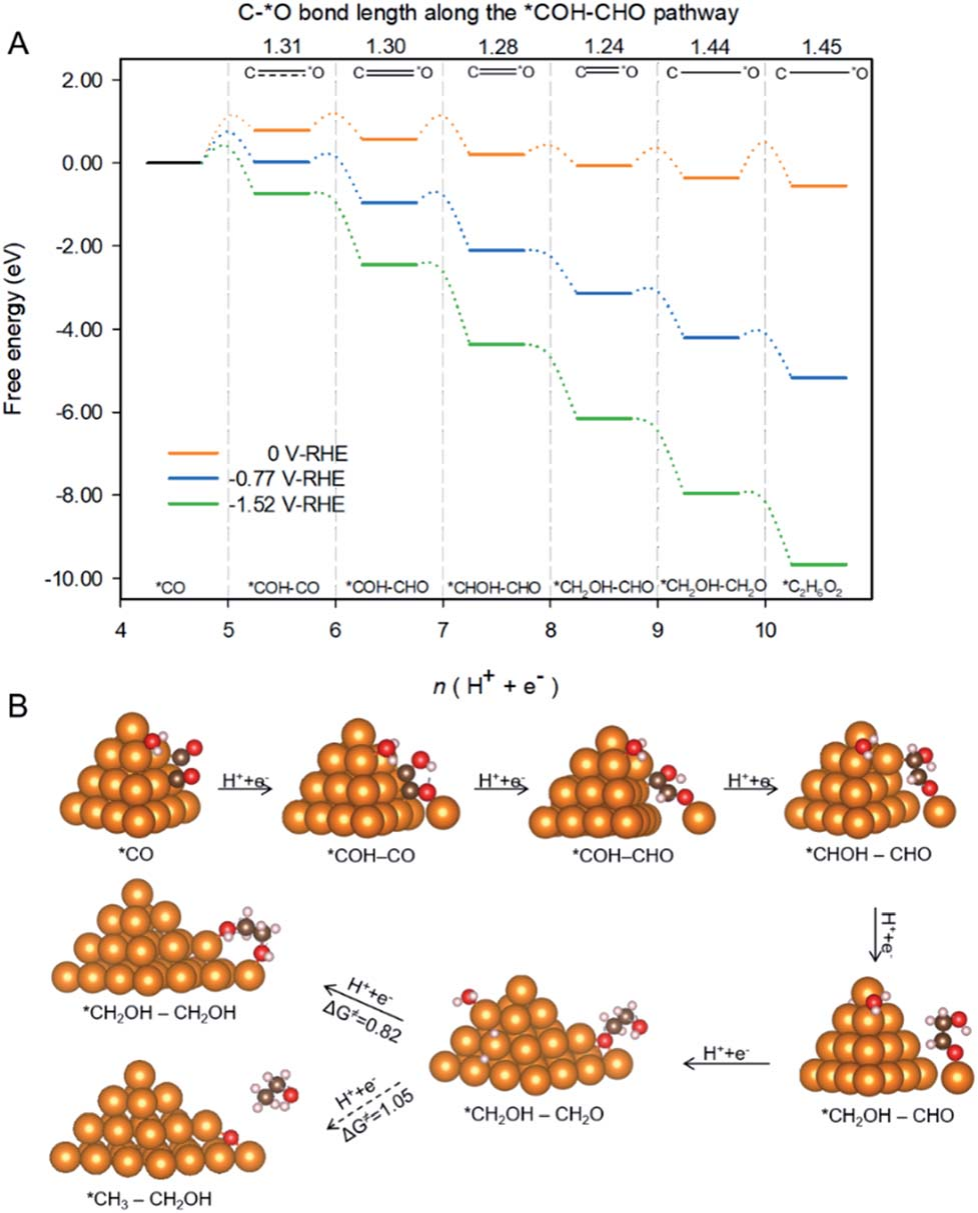
Reduction of *CO to ethylene glycol on Cu-DAN. (A) Preferred pathway and associated reaction energetics identified for production of CH_2_OH–CH_2_OH starting with 2 *CO on nanopyramid surface at 0, −0.77 and −1.52 V, *vs.* RHE. The C–O bond length (in Å) along the pathway is also given. This illustrates that the double bond between βC and βO is maintained until formation of *CH_2_OH–CH_2_O. (B) Atomic structures of the reaction intermediates along the pathway. The slashed-arrow indicates kinetically unfavorable selection toward ethanol due to higher activation barrier compared with EG (1.05 eV *vs.* 0.82 eV). Color code: Cu, orange; C, brown; O, red; H, pink.

Spatial-confinement also protects βO by maintaining the double bond between βC and βO until formation of *CH_2_OH–CH_2_O as is shown in Fig. 5A. The figure depicts the pathway and associated reaction energetics for ethylene glycol production starting with 2 *CO on Cu-DAN at 0 V, −0.77 V (the onset potential with 0.75 eV barrier threshold), and −1.52 V *vs.* RHE (the onset potential corresponding to 0.40 eV barrier threshold). The atomic structures of intermediates in the pathway are shown schematically in Fig. 5B where the βC–O bond length is used to estimate bond strength in the intermediates along the pathway.^26^ The βC–O bonds in the first three post *COH–CO intermediates along the pathway *i.e.* *COH–CHO, *CHOH–CHO and *CH_2_OH–CHO, can be classified as strong bonds with a bond length shorter than 1.30 Å, a typical C=O double bond. A strong C=O double bond hinders protonation of βO and subsequent dehydroxylation. It therefore reserves βO atoms in these intermediates.

Additionally, as a result of kinetic selectivity derived from spatial-confinement the hydrogenation preferentially happens to αC instead of αO, until the second-last step where αC is completely disconnected from original nanopyramid, Fig. 5B. Without the restriction of confined-space the βC=O double bond is facilely stretched, and turns into a single bond with a bond length longer than 1.44 Å. This facilitates hydrogenation of βO to yield the final product – ethylene glycol – with downhill Δ*G* = −0.19 eV.

### Post *CH_2_OH–CH_2_O to ethylene glycol due to spatial-confinement

The final step toward formation of ethylene glycol has a competing pathway which is the formation of CH_3_–CH_2_OH *via* an exothermal protonation of βC (Δ*G* = −0.58 eV at 0 V *vs.* RHE). However, the reaction barrier for the step (1.05 eV, Fig. S11†) is greater than that for ethylene glycol (0.82 eV, Fig. S12†), indicating that the production of ethanol is kinetically less favorable. Moreover the reaction barrier for the final step toward ethylene glycol is further reduced to 0.44 eV under potential of −0.77 V applied to facilitate the previous C–C coupling step. This is below the 0.75 eV threshold, and can therefore be overcome to give appreciable production rates at room temperature.^40^

In the pathway toward ethylene glycol, the two oxygen atoms have substantial binding with copper (O–Cu distance of ∼2.0 Å, as is shown schematically in Fig. S12†). This finding reveals that the hydrogenation of βO does not break the O–Cu bond. In contrast the hydrogenation of βC results in the breaking of O–C bond and the adsorbate *i.e.* ethanol is driven further away from the surface as a result of the adverse alignment of OH group dipole moment with charged surface (Fig. S11†).^28^ Therefore it is more kinetically facile to form the surface bound ethylene glycol because the process does not involve bond breaking. Additionally the desorption of ethylene glycol from nanopyramid surface is slightly endothermic at 0.28 eV, and can be readily overcome.

Overall, the extra binding between O and Cu that occurs because of the confined-space amongst adjacent nanopyramids promotes selectivity toward ethylene glycol by facilitating an alternative pathway of three parts: (1) the atomic arrangement with an extra O–Cu bond kinetically suppresses the other two competing pathways by increasing relevant reaction barriers; (2) greater electron transfer *via* O–Cu bond and consequent greater nucleophilicity on βC lowers the formation barrier of key intermediate *COH–CHO. This is the foundation for the new pathway, and; (3) spatial-confinement facilitates the extra O–Cu binding that preserves both O atoms against dehydroxylation through geometrically selecting hydrogenation toward formation of ethylene glycol.

## Conclusions

New reaction mechanisms and alternative pathways based on DFT computations for the electroreduction of CO_2_ to ethylene glycol on densely-arrayed Cu nanopyramids (Cu-DAN) show that there exists an alternative pathway that facilitates the direct electrosynthesis of ethylene glycol. This pathway is not favorable on planar Cu(100) surface and sparsely-arrayed nanopyramids without an extra binding between O atom and surface Cu atom from the adjacent nanopyramid. It is concluded that the extra O–Cu bond: (1) promotes C–C coupling; (2) reserves both O atoms against dehydroxylation through spatial-confinement; (3) disadvantages both two conventional electroreduction pathways; and, (4) facilitates an alternative pathway to directly synthesize ethylene glycol. Findings highlight the importance of forming Cu nanopyramids with desired morphologies that are carefully tuned to modify the activity and selectivity of electrocatalytic CO_2_ reduction reactions. These will aid practical efforts to increase selectivity toward poly-hydroxyl oxygenates, and be of immediate benefit in the design of highly active and selective electrocatalysts for CO_2_ electroreduction *via* morphology control.

## Author contributions

L. C. carried out the theoretical simulation and data analysis. C. T. carried out part of the simulation. K. D. and Y. Z. carried out part of the data analysis. Y. J. and S. Z. Q. conceived and supervised the research. Y. J. designed the theoretical simulation, and directed the manuscript writing and revision. All co-authors assisted in writing and revising the manuscript. All co-authors read and approved the final manuscript.

## Conflicts of interest

There are no conflicts to declare.

## Supplementary Material

SC-012-D1SC01694F-s001
