# Supplementary material for: Spatial-confinement induced electroreduction of CO and CO2 to diols on densely-arrayed Cu nanopyramids
Source: Chem Sci. 2021 May 7;12(23):8079–87. doi: 10.1039/d1sc01694f (PMC8208127; doi:10.1039/d1sc01694f)
Supplement: SC-012-D1SC01694F-s001 [file SC-012-D1SC01694F-s001.pdf]

Electronic Supplementary Material (ESI) for Chemical Science.
This journal is © The Royal Society of Chemistry 2021

Spatial-Confinement Induced Electroreduction of CO and CO_2 to Diols on Densely-Arrayed Cu Nanopyramids

Ling Chen, Cheng Tang, Kenneth Davey, Yao Zheng, Yan Jiao* and Shi-Zhang Qiao*

School of Chemical Engineering and Advanced Materials, The University of Adelaide, South Australia 5005, Australia

Corresponding author: * yan.jiao@adelaide.edu.au; s.qiao@adelaide.edu.au

This file includes:

- **Schemes S1-S2**
- **Figures S1-S20**
- **Supplementary Notes 1-4**
- **Tables S1-S15**
- **Supplementary References**

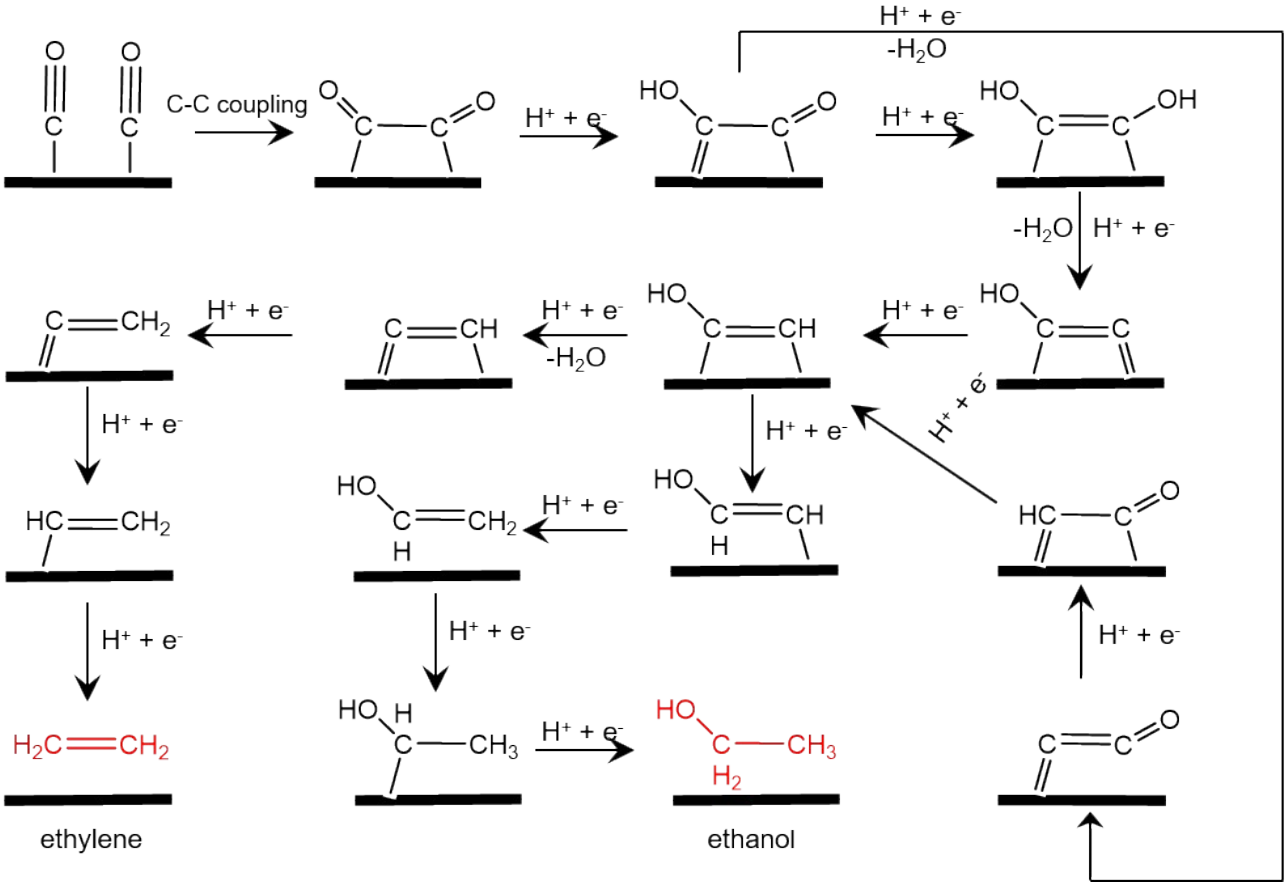

Scheme S1. Conventional *COH–COH and *C–CO pathways on Cu (100) following adsorption of two CO intermediates.[1-3]

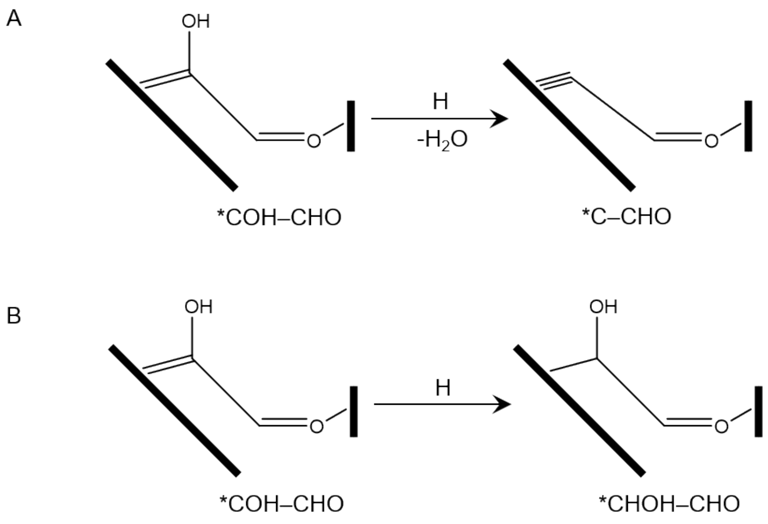

Scheme S2. Further hydrogenation step for *COH–CHO on Cu-DAN to: (A) *C–CHO, and; (B) *CHOH–CHO.

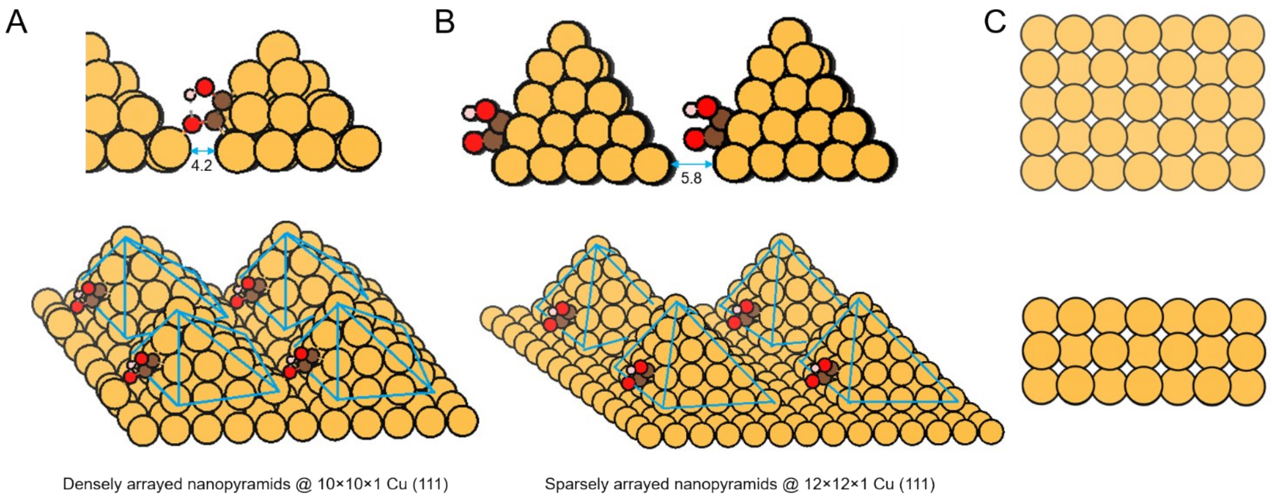

Fig. S1. Close-up (top panel) and overall (bottom panel) view of *COH–CO adsorption on: (A) densely-arrayed Cu nanopyramids, (B) sparsely-arrayed Cu nanopyramids, and top and side view of (C) planar Cu (100). The [111] diamond nanopyramid was built based on a 10×10×1 Cu surface with 4.2 Å distance between two nanopyramids to represent the dense-array, and on a 12×12×1 Cu surface to represent the sparse-array with 5.8 Å distance. Distance in figure is Å. Color code: Cu, orange; C, brown; O, red; H, pink. Solid-blue lines serve as a visual guide for the pyramid.

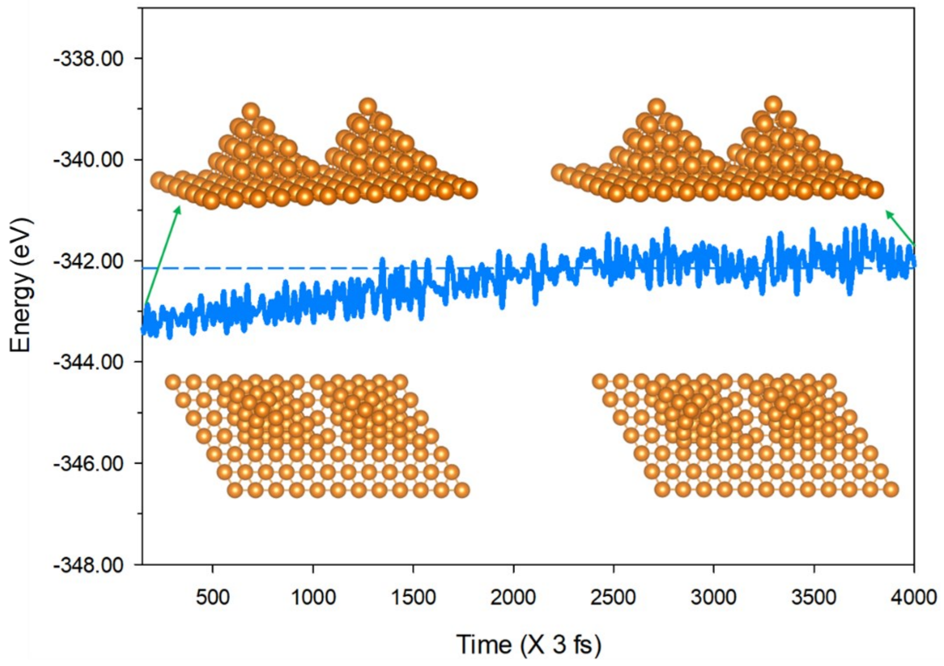

Fig. S2. *Ab initio* molecular dynamics (AIMD) simulated energy evolution for Cu-DAN at 300 K. Insets show the top and side views of the snapshots of atomic configurations at 0 and 12 ps. The blue dashed-line denotes equilibrium position.

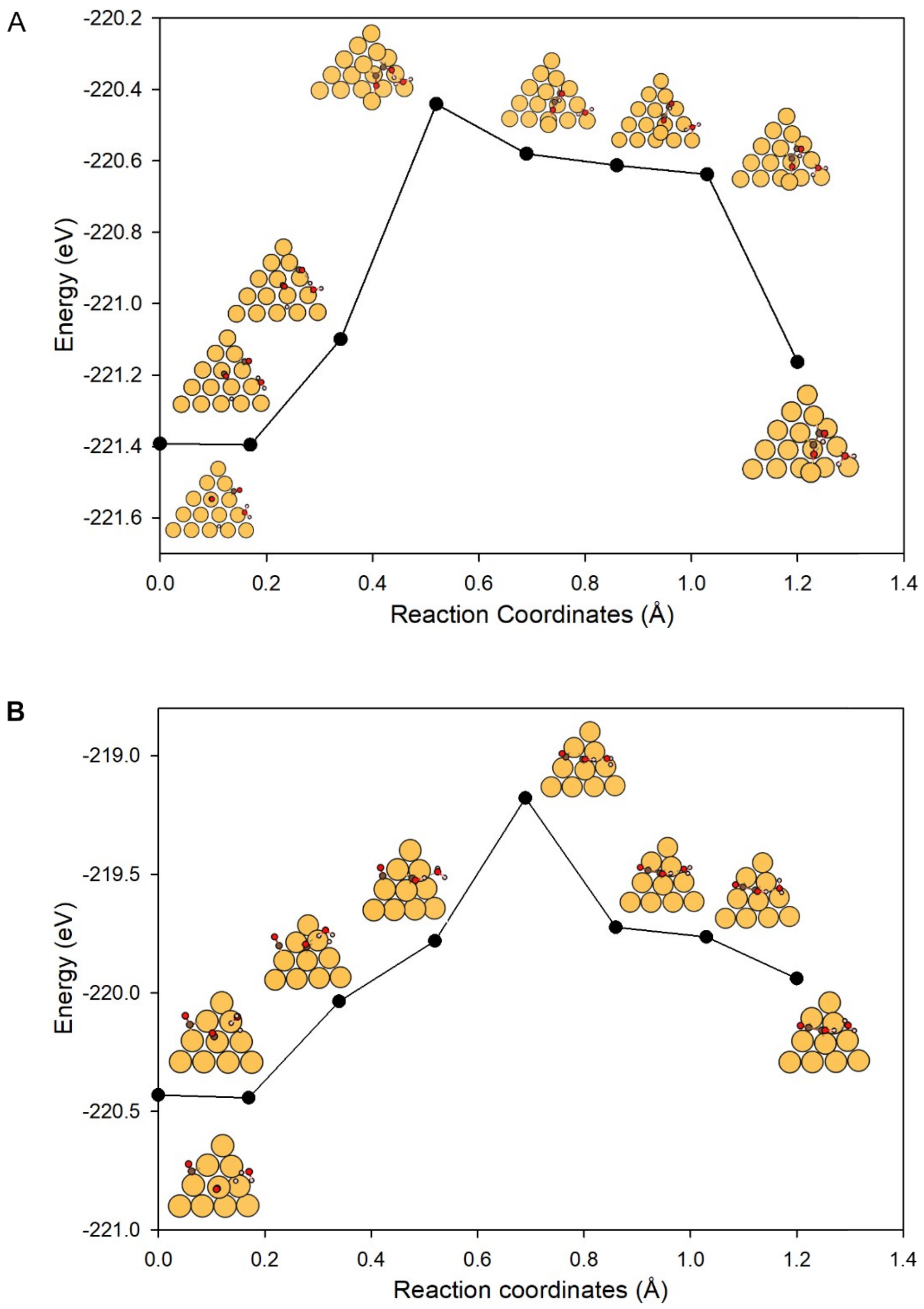

Fig. S3. Minimum energy path (MEP) involved in *CO + *CO + H^+ + e^- → *COH–CO via H-shuttling on (A) Cu-DAN, and (B) Cu-SAN. Color code: Cu, orange; C, brown; O, red; H, pink. Energy barrier in (A) is $\Delta G^{\neq}(U^0)$ = 1.07 eV and in (B), $\Delta G^{\neq}(U^0)$ = 1.50 eV.

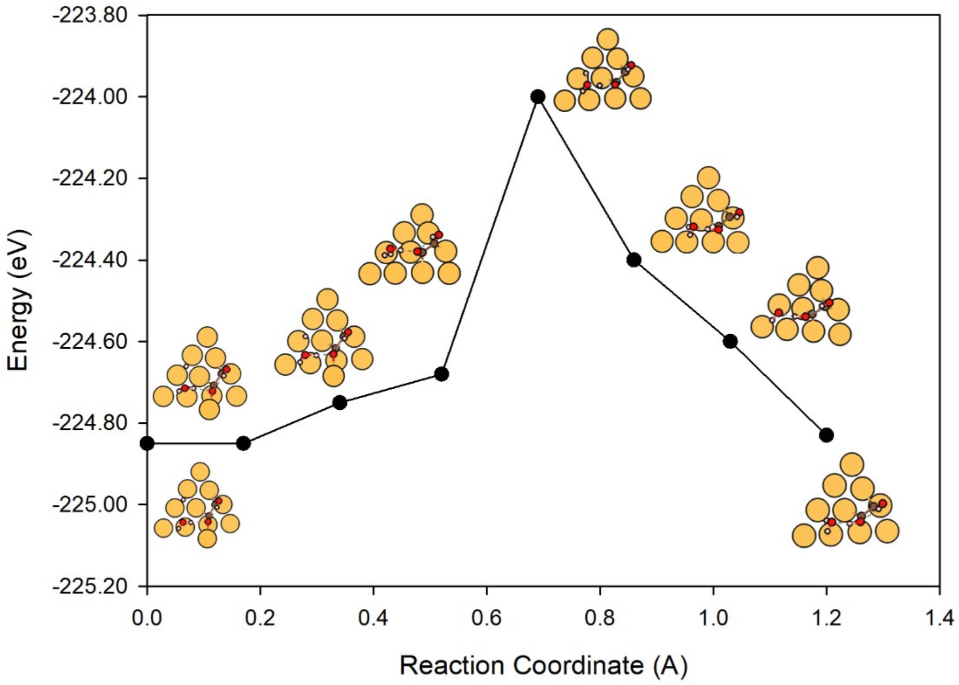

Fig. S4. MEP involved in *COH–CO + H^+ + e^- → *COH–COH on Cu-DAN via H-shuttling. Color code: Cu, orange; C, brown; O, red; H, pink. Energy barrier is $\Delta G^{\neq}(U^0)$ = 0.76 eV.

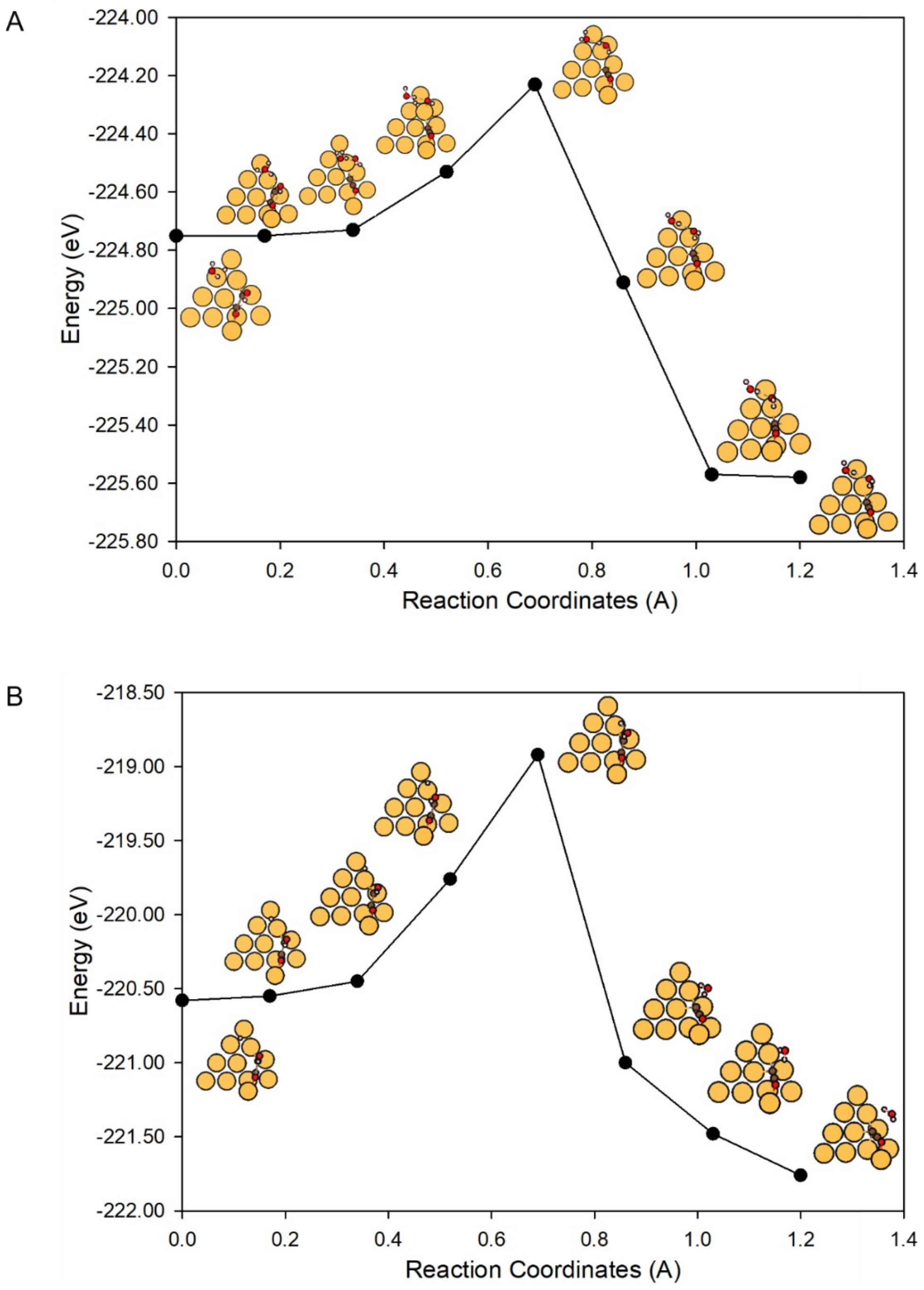

Fig. S5. MEP involved in *COH–CO + H^+ + e^- → *C–CO + H_2O on Cu-DAN via (A) H-shuttling and (B) surface *H transfer. Color code: Cu, orange; C, brown; O, red; H, pink. Energy barrier value in (A) is $\Delta G^{\neq}(U^0)$ = 0.72 eV and in (B), $\Delta G^{\neq}(U^0)$ = 1.66 eV

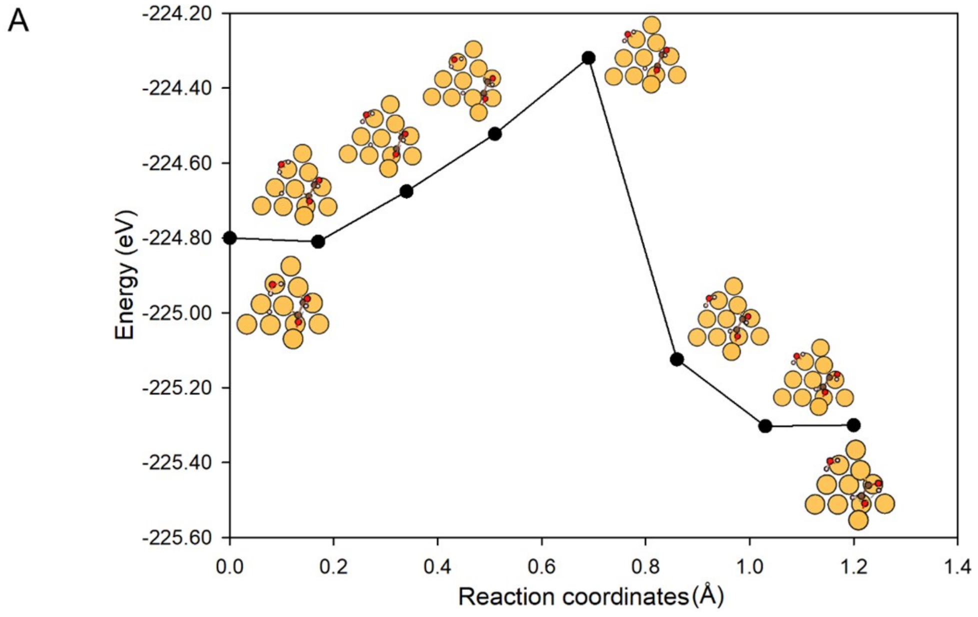

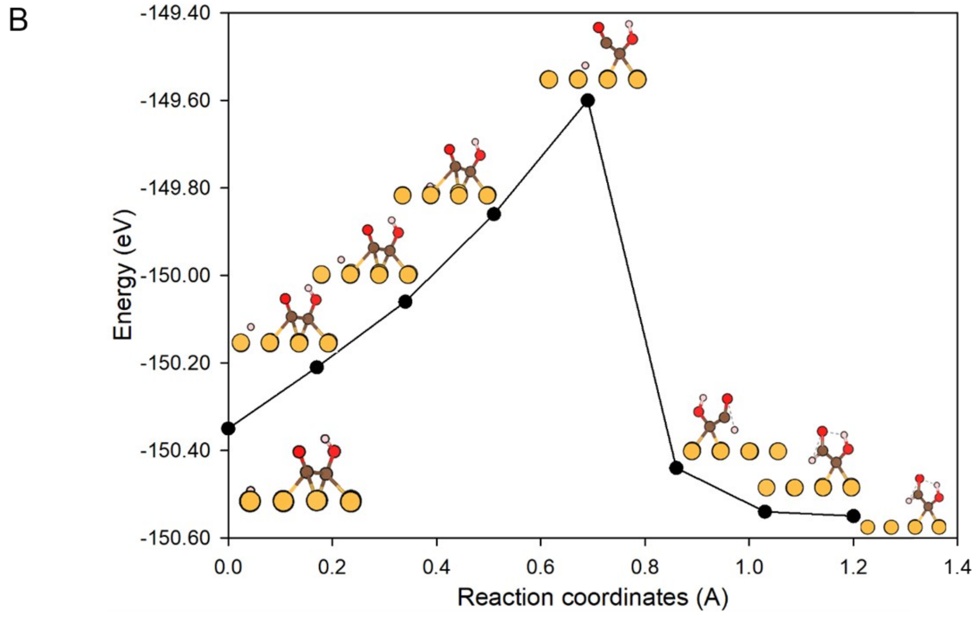

Fig. S6. MEP involved in *COH–CO + H^+ + e^- → *COH–CHO via surface *H transfer on (A) Cu-DAN and (B) planar Cu (100). Color code: Cu, orange; C, brown; O, red; H, pink. Energy barrier in (A) is $\Delta G^{\neq}(U^0)$ = 0.41 eV, in (B) $\Delta G^{\neq}(U^0)$ = 0.69 eV.

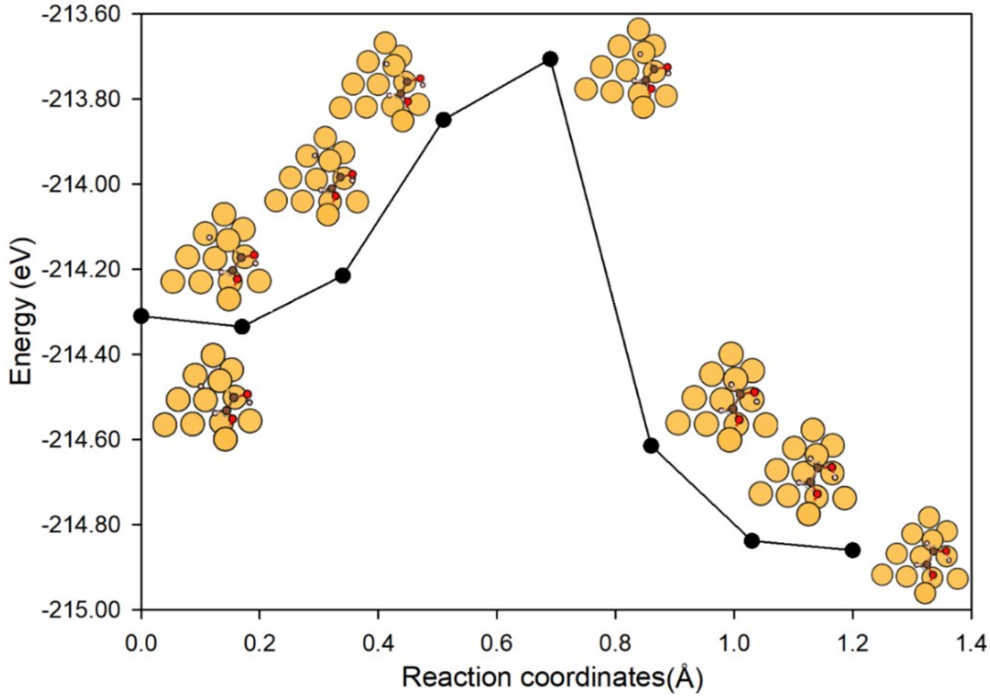

Fig. S7. MEP involved in *COH–CHO + H^+ + e^- → *CHOH–CHO via surface *H transfer on Cu-DAN. Color code: Cu, orange; C, brown; O, red; H, pink. Energy barrier value is $\Delta G^{\neq}(U^0) = 0.52$ eV.

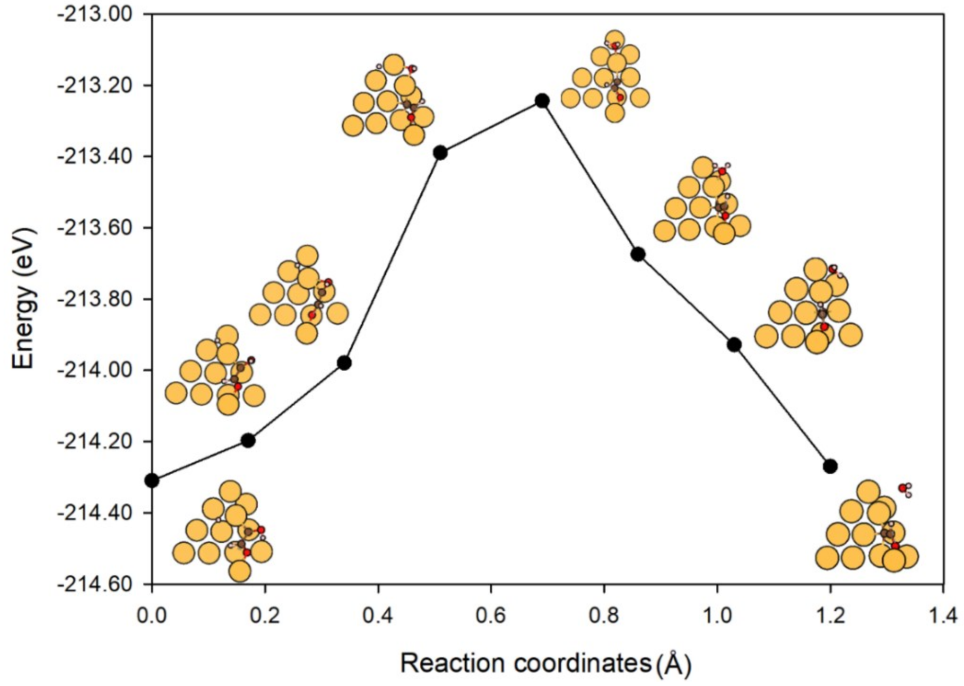

Fig. S8. MEP involved in *COH–CHO + H^+ + e^- → *C–CHO + H_2O on Cu-DAN via surface *H transfer. Color code: Cu, orange; C, brown; O, red; H, pink. Energy barrier value is $\Delta G^{\neq}(U^0)$ = 1.02 eV.

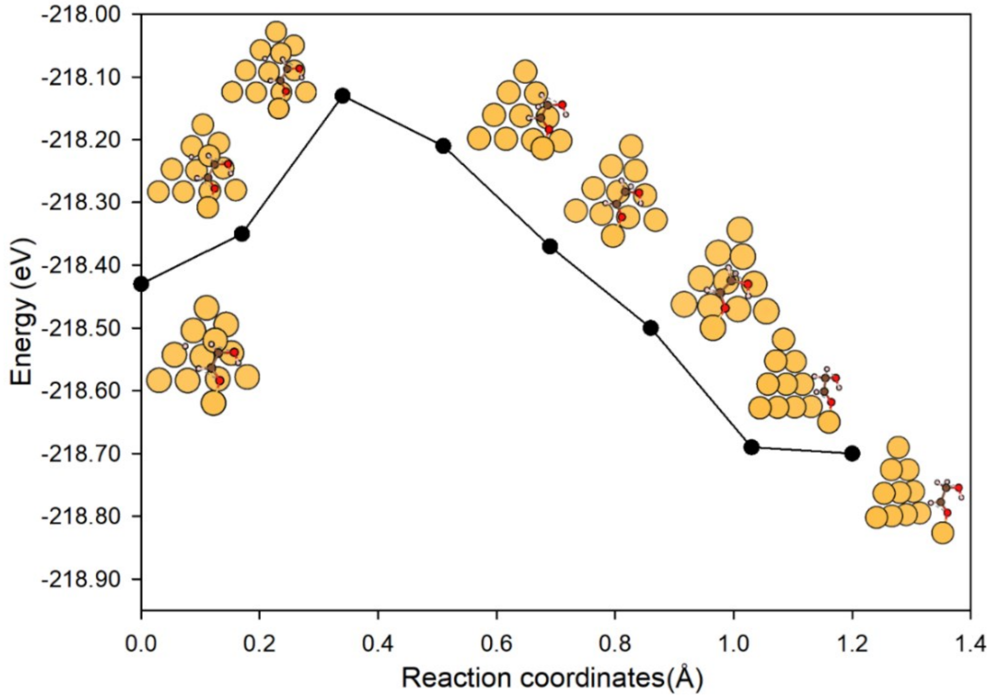

Fig. S9. MEP involved in *CHOH–CHO + H^+ + e^- → *CH_2OH–CHO on Cu-DAN via surface *H transfer. Color code: Cu, orange; C, brown; O, red; H, pink. Energy barrier value is $\Delta G^{\neq}(U^0)$ = 0.25 eV.

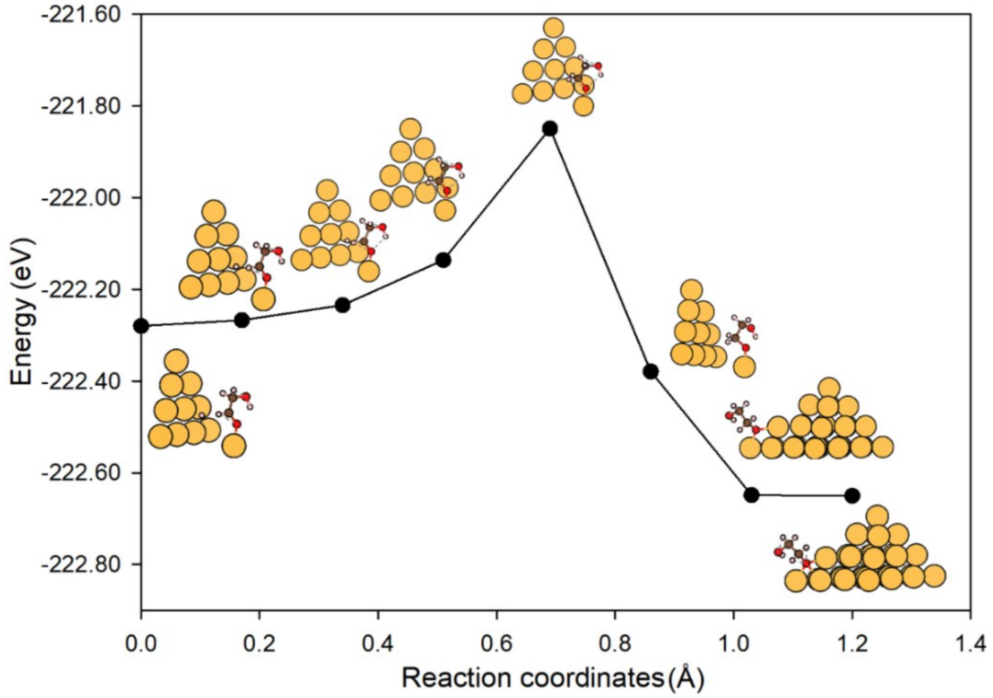

Fig. S10. MEP involved in $*CH_2OH–CHO + H^+ + e^- \rightarrow *CH_2OH–CH_2O$ on Cu-DAN via surface *H transfer. Color code: Cu, orange; C, brown; O, red; H, pink. Energy barrier value is $\Delta G^{\neq}(U^0) = 0.39$ eV.

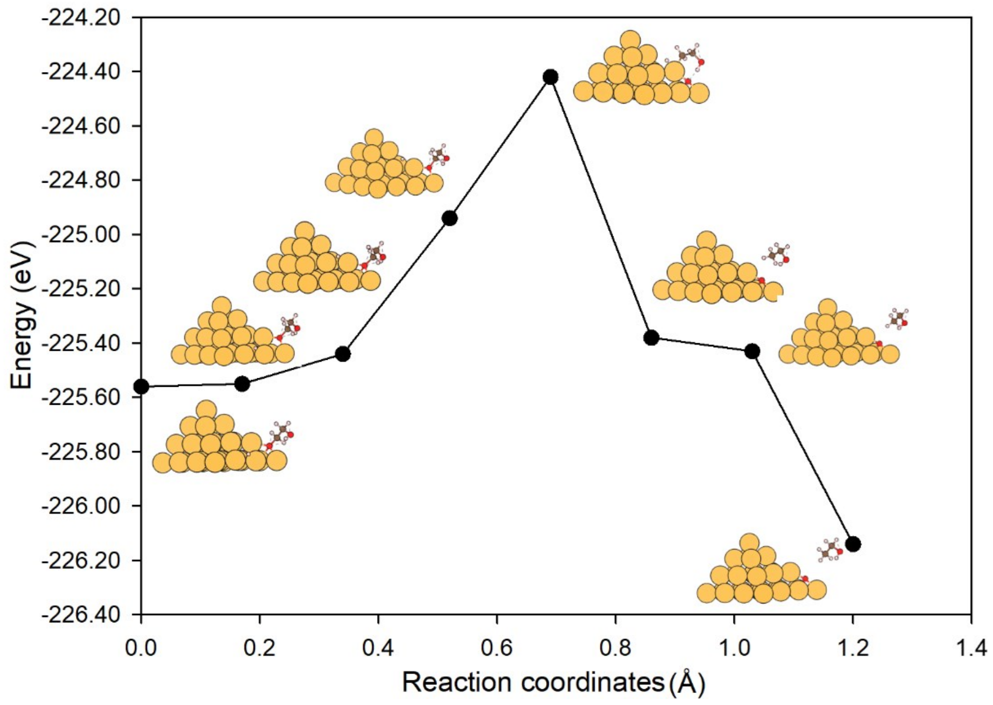

Fig. S11. MEP involved in $*CH_2OH–CH_2O + H^+ + e^- \rightarrow CH_2OH–CH_3 + *O$ on Cu-DAN via surface *H transfer. Color code: Cu, orange; C, brown; O, red; H, pink. Energy barrier value is $\Delta G^{\neq}(U^0)$ = 0.98 eV.

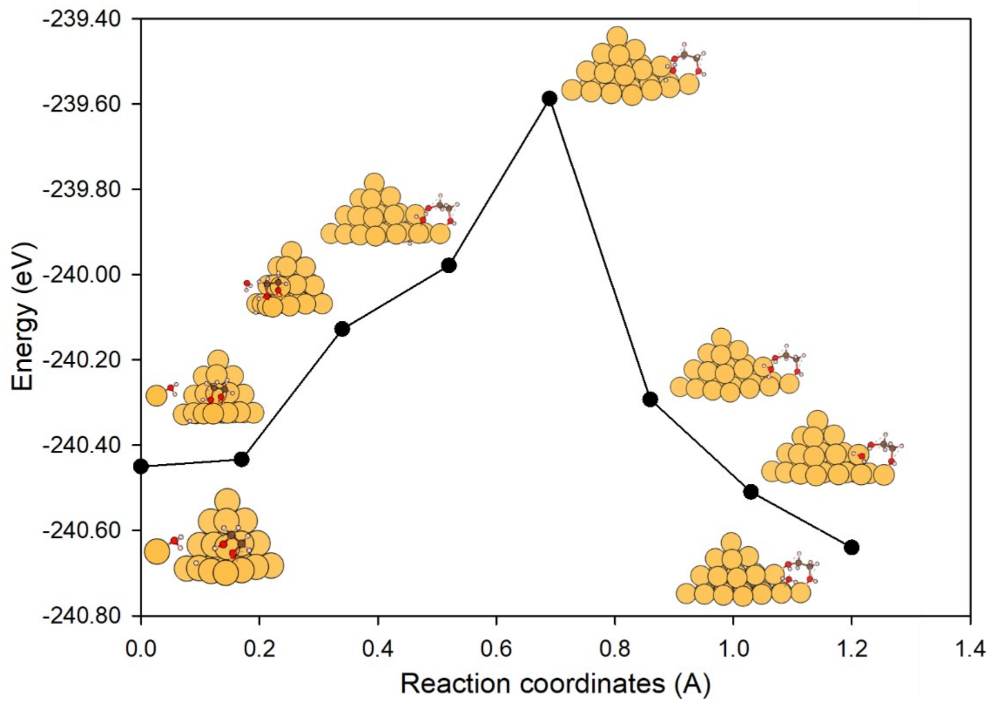

Fig. S12. MEP involved in $*CH_2OH–CH_2O + H^+ + e^- \rightarrow *CH_2OH–CH_2OH$ on Cu-DAN via H-shuttling. Color code: Cu, orange; C, brown; O, red; H, pink. Energy barrier value is $\Delta G^{\neq}(U^0)$ = 0.73 eV.

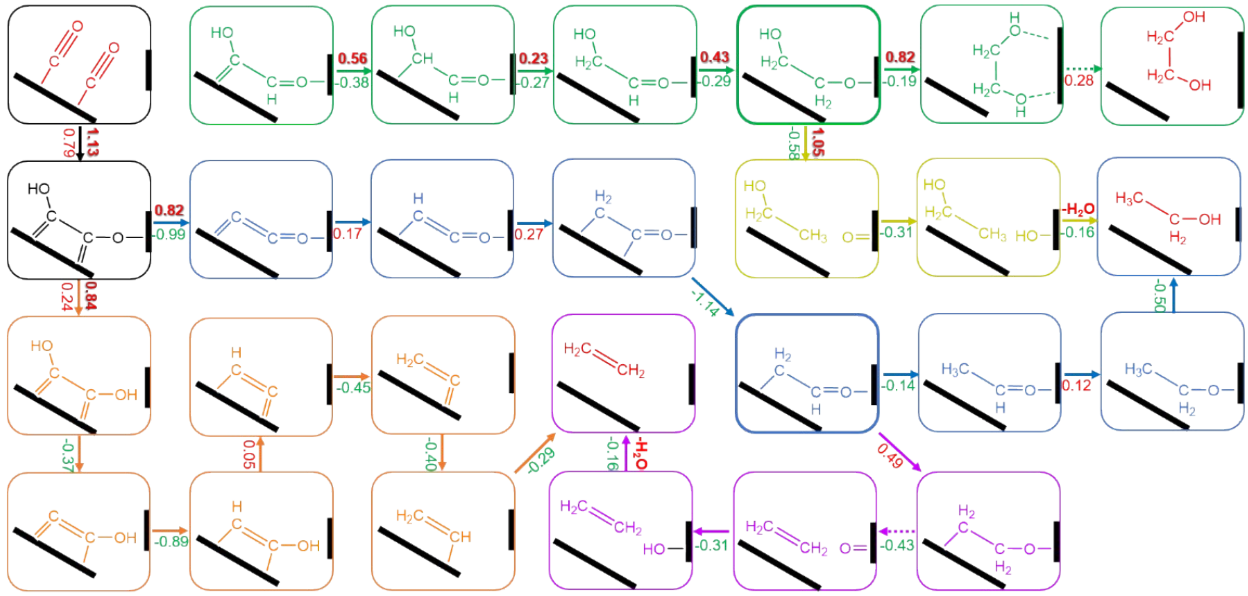

Fig. S13 Chemical structure of reaction intermediates in different pathways on Cu-DAN. Pathways toward C_2 products (ethylene, ethanol and ethylene glycol) beyond 2 *CO are shown as colored-branches: orange, *COH–COH pathway toward C_2H_4; blue, *C–CO pathway toward C_2H_5OH; purple, *C–CO pathway bifurcating to C_2H_4; green, *COH–CHO pathway toward $C_2H_6O_2$, and; brown, *COH–CHO pathway bifurcating to C_2H_5OH. Reaction barrier $\Delta G^{\neq}$ values at 0 V vs RHE appear in bolded- and shaded-font. Free energy change ΔG values appear in standard-font. Green and red values denote, respectively, exergonic and endergonic process. The solid-arrow denotes proton-coupled electron transfer ($H^+ + e^-$), and dotted-arrow denotes desorption. The unit of energy is eV.

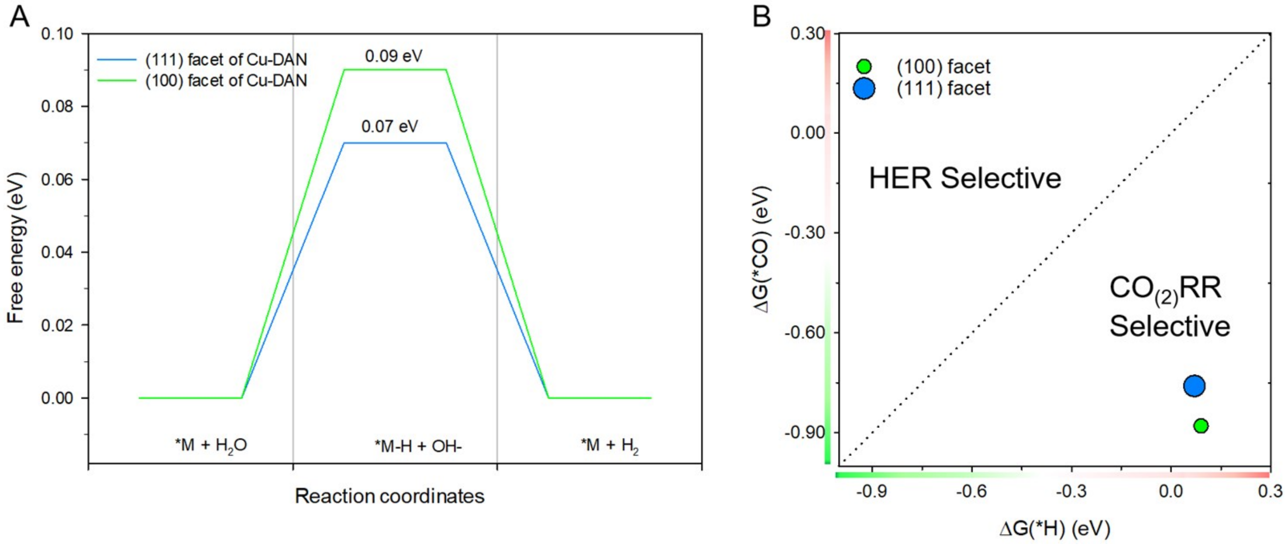

Fig. S14. (A) Free energy diagram for HER on Cu-DAN. (B) Comparison between hydrogen adsorption free energy ΔG(*H) and carbon monoxide adsorption free energy ΔG(*CO) on (111) and (100) facets of Cu-DAN.

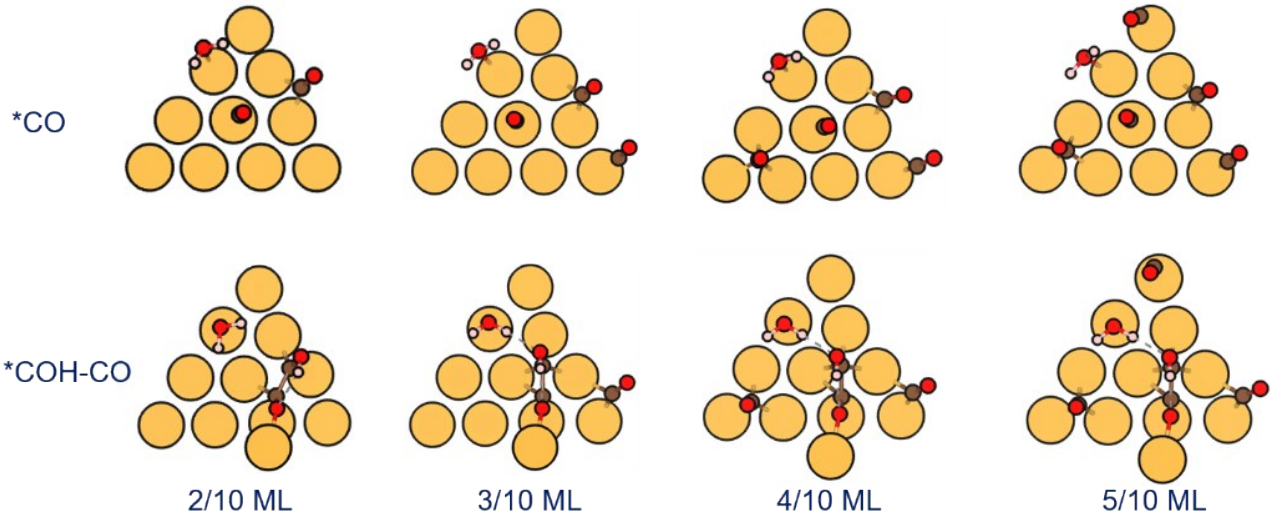

Fig. S15. Atomic structures of *CO and *COH–CO under *CO coverage of 2/10, 3/10, 4/10 and 5/10 ML. Color code: Cu, orange; C, brown; O, red; H, pink.

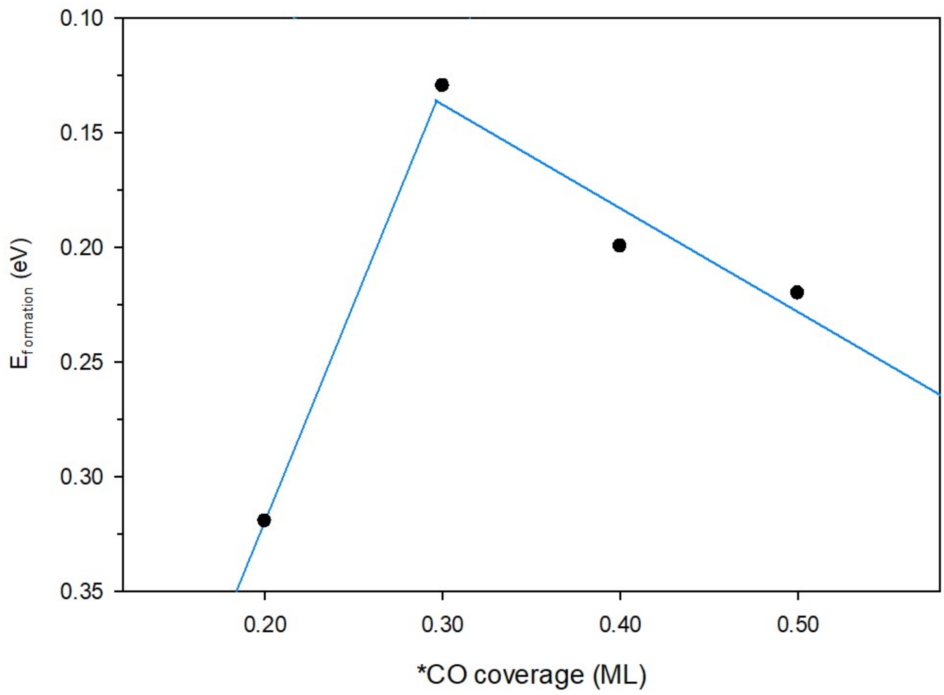

Fig. S16. Volcano-type relationship between *CO coverage and formation energy of *COH–CO.

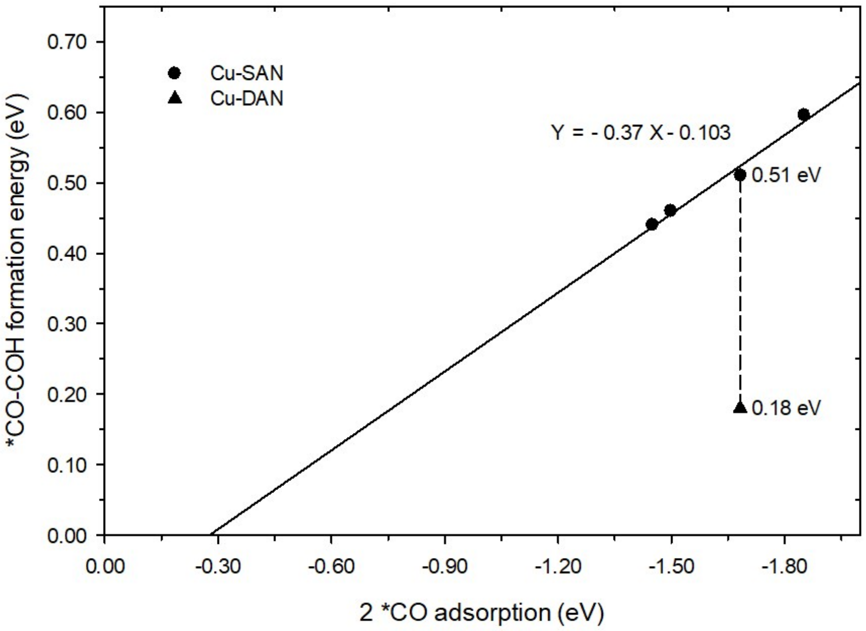

Fig. S17. Linear scaling relations between 2*CO adsorption and *COH–CO formation free energies on Cu-SAN and Cu-DAN.

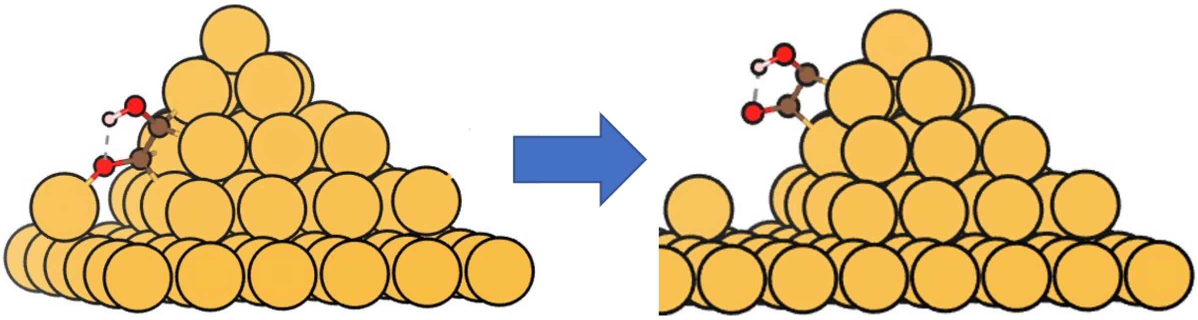

Fig. S18. Computation of Cu–O bond strength. To compute the strength of the bond between βO and Cu of the adjacent nanopyramid, we moved the *COH–CO along the nanopyramid surface until the Cu-O bond was completely broken. The energy difference between the translated structure with original is 2.63 eV (253.76 kJ mol^{-1}), and reflects the Cu–O bond strength. All atoms were fixed to compute the single-point energy of the structure.

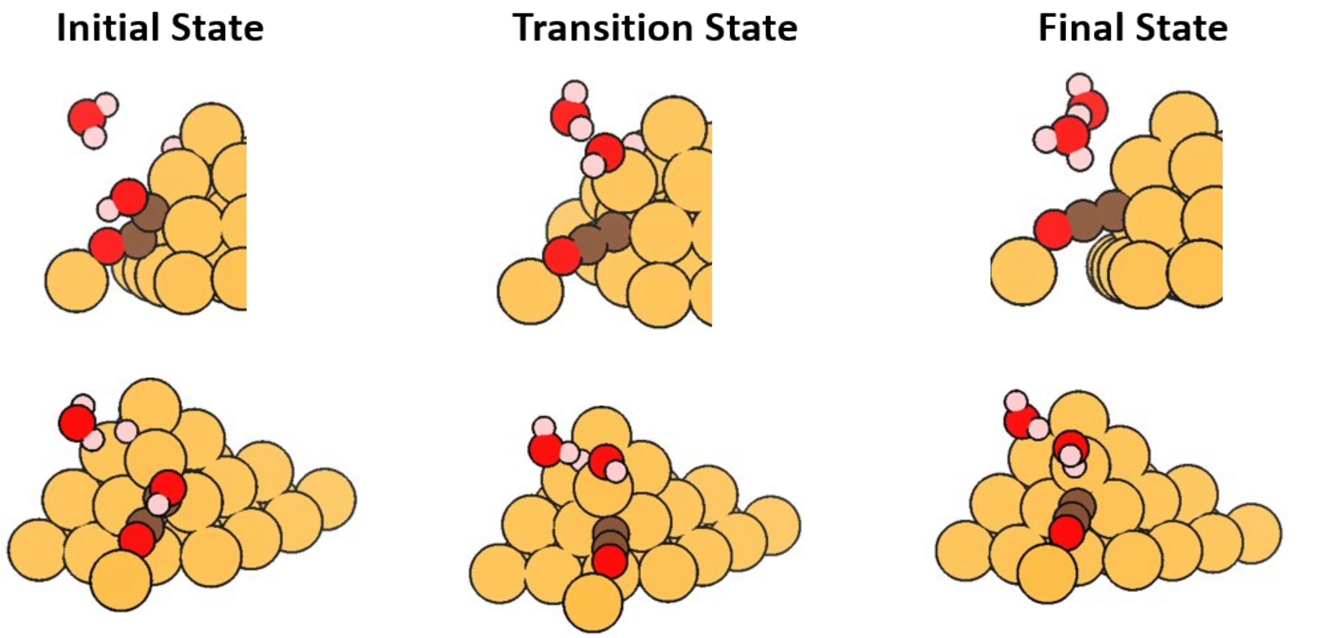

Fig. S19. Side and front view of optimized structures of, respectively, the initial, transition and final states involved in *COH–CO reduction to *C–CO on Cu-DAN. Color code: Cu, orange; C, brown; O, red; H, pink.

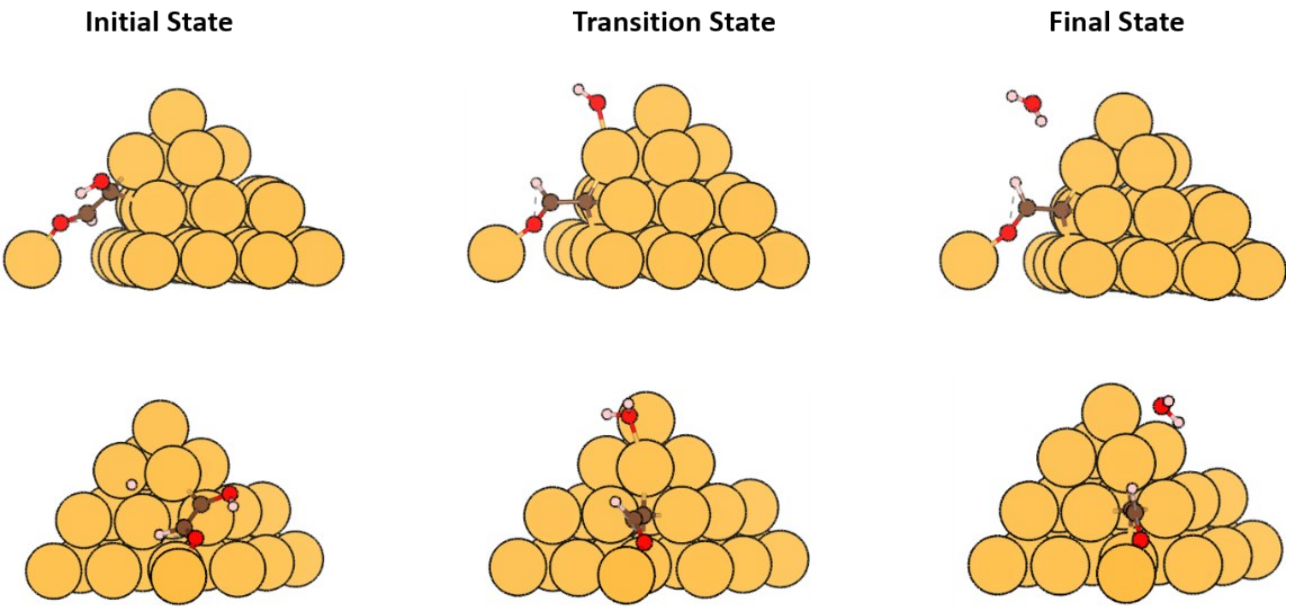

Fig. S20. Side and front view of optimized structures of, respectively, the initial, transition and final states involved in *COH–CHO reduction to *C–CHO on Cu-DAN (*COH–CHO + H^+ + e^- → *C–CHO + H_2O). Color code: Cu, orange; C, brown; O, red; H, pink.

Supplementary Note 1

Stability of Cu-DAN

Stability is a prerequisite for electrocatalytic materials. This is closely related to the experimental feasibility and catalytic activity for long-term use.

The dissolution potential (U_{diss}) of the surface atoms on nanopyramid, which assess the dissolution-resistance of one surface atom from the nanopyramid into water under an applied potential,[8] is calculated as:

$$U_{diss} = U^0_{diss(Cu)} - E_f / ne$$

where $U^0_{diss(Cu)}$ and n denote standard reduction potential of Cu and the number of electrons transferred during dissolution. The values are 0.34 V and 2, respectively.

E_f, the formation energy of one surface Cu atom, is defined as:

$$E_f = E_{nanopyramid} - E_{less} - E_{Cu}$$

where $E_{nanopyramid}$ is the total energy, E_{less} is the energy of nanopyramid without one surface Cu atom and E_{Cu} is the energy of isolated Cu atom.

The calculated positive U_{diss} of 1.72 V shows that the surface Cu atoms will be stable under electrochemical environments.

In addition, we carried out *ab initio* molecular dynamics (AIMD) simulations to explore the thermodynamic stability of the surface atoms of the Cu-DAN at 300K, as shown in Figure S2. We found that the thermal oscillations of surface Cu atoms occur near the equilibrium position following relaxation for 6 ps, and that the structure showed no significant structural distortion. This finding confirms thermodynamic stability of Cu-DAN.

Supplementary Note 2

Hydrogen evolution reaction (HER) on Cu-DAN

We used DFT computations to identity that the most stable *H adsorption sites, with minimum adsorption energy on Cu nanopyramids, is the 4-fold hollow site for (100) facets, and HCP hollow site for (111) facets, respectively. The performance of these active sites for the Volmer step of HER in alkaline condition (*M + H_2O + e^- →*M-H + OH^-) was investigated using free energy as an indicator. The free energy change of the Volmer step can be computed from:

$$\Delta G_{volmer} = (G_{*M-H} + G_{OH-}) - (G_{*M} + G_{H2O} + G_{e^-})$$

where G_{*M} is the free energy of the original pyramidal structure, $G_{*M\text{-}H}$ is the free energy of the pyramidal structure with adsorbed *H, G_{H2O} and G_{H2} is the free energy of a H_2O molecule and a H_2 molecule. G_{OH-} is the free energy of a hydroxide ion (OH^-) obtained as, $G_{OH-} = G_{H2O} - G_{H2}/2$.

The computational results are given in Figure S14A, and a comparison with *CO adsorption energy on the same sites is given in Figure S14B that indicates suppression of competing HER on Cu-DAN.

Supplementary Note 3

Effect of *CO coverage on the formation of *COH-CO

The coverage of *CO is expressed in monolayer (ML) units. These are defined as the number of adsorbed *CO molecules (*n, n* = 2, 3, 4 and 5 in this simulation) divided by the total number of atoms on one facet of Cu-DAN (10). The formation energy of intermediate *COH-CO under such *CO coverage then is computed as:

$$E_{formation} = E_{total} - (E_{slab+n*CO} + E_{H2}/2)$$

where E_{total} is the energy of the total system, $E_{slab+n*CO}$ is the energy of Cu-CAN with adsorption of n *CO molecules. E_{H2} is the energy of a hydrogen molecule (-6.76 eV).

We began by constructing the Cu-DAN surface with two *CO adsorbates (*CO coverage = 2/10 ML). To investigate the influence of *CO coverage on the formation of *COH–CO we added more *CO molecules on the surface to gradually increase the *CO coverage from 2/10 to 5/10 ML as is shown in Figure S15. We found volcano-type relationships between *CO coverage and formation energy of *COH–CO (Figure S16). With the increase of *CO coverage, the formation energy of *COH–CO increases first and reaches a peak at 3/10 ML. This is consistent with experimental observations that a higher *CO coverage promotes C–C coupling.

As the *CO coverage increases further, the formation energy of *COH–CO decreases. This is because the steric hindrance of O atom caused by excessive *CO adsorption destabilizes the dimer intermediate *COH–CO.

Supplementary Note 4

*COH–CHO formation on planar Cu (100)

*COH–CHO is the precursor of glycolaldehyde (CH_2OH–CHO). Two mechanisms have been hypothesized toward its formation on planar Cu surface.[1] These are: 1) *COH–CO + H → *COH–CHO, and; 2) *CHO + *COH → *COH–CHO. On planar Cu (100) surface *COH–COH prevails over *COH–CHO. The reason is discussed in detail.

For the first mechanism Cheng et al.[1] hypothesized a pathway on planar Cu (100) facets in which *COH–CO formation is followed by hydrogenation of βO atoms. This rules out the possibility of hydrogenation of βC to *COH–CHO. We attribute this to the instability of intermediate *COH–CHO. The structures of *COH–CHO and *COH–COH are tautomeric. The former is comparatively unstable because it is vertically adsorbed on surface via a double bond, and because it has a free radical on the C atom. We computed *COH–CHO to be 0.26 eV less stable than *COH–COH on planar Cu (100) surface.

This finding means the bulk of *COH–CHO are spontaneously tautomerized to *COH–COH, despite being formed first. Because *COH–CHO is not a stable molecular compound with a significant concentration on planar Cu (100) surface, the *COH–CHO pathway is blocked. Additionally if the formation of *COH–CHO follows the second mechanism, it is difficult for it to proceed on a planar surface. *COH and *CHO are preferably, respectively, stabilized on Cu (111) and Cu (100) surface.[4]

It is reportedly rare for those two adsorbates to simultaneously attain the kinetically reactable concentration on a homogeneous facet. This is the reason why we record a high-level barrier of 0.72 eV at 0 V vs RHE for *COH–CHO formation on planar Cu (100) surface.

We conclude therefore that on a planar Cu (100) surface *COH–CHO is not favored and the pathway is blocked.

Table S1. Free energy for gas phase species.

Gas Molecule	***E*** **(eV)**	***ZPE*** **(eV)**	***–TS*** **(eV)**	***G*** **(eV)**
H_2 (g)	–6.75	0.27	–0.41	–6.89
H_2O (g)	–14.22	0.56	–0.67	–14.33
CO (g)	–14.80	0.13	–0.61	-14.70
CO_2 (g)	–23.01	0.31	–0.66	–23.36
C_2H_4 (g)	–31.97	1.37	–0.55	–31.15
C_2H_5OH (g)	–46.88	2.13	–0.60	–45.35
$(CH_2OH)_2$ (g)	–53.31	2.27	–0.65	–51.69

Table S2. Summary of elementary reaction steps, hydrogen transfer model, reaction barriers at various electrode potentials and involved parameter for computation. ΔG(0V) and $\Delta G^{\neq}$(0V) are the free energy change and reaction barrier without potential bias, $\Delta G^{\neq}(U^0)$ is the reaction barrier under U^0. U^0 is the equilibrium potential for the reductive adsorption of one proton in the system, and β' is the reaction symmetry factor, as defined in the manuscript. The two hydrogen models considered were, 1) Langmuir-Hinshelwood (LH) mechanism (i.e. direct transfer of an adsorbed *H), and 2) Eley-Rideal (ER) mechanism (i.e. water molecule shuttles an adsorbed *H). Adding hydrogen to carbon species via LH mechanism always gives lower kinetic barriers, comparied with hydrogen transfer via ER mechanism. ER mechanism contributed to a lower kinetic barrier when hydrogen is added to oxygen species.[5,6] For all steps computed with H-shuttling, one H_2O molecule was included in the computation.

No.	Catalyst	Reaction Steps	Model	$\Delta G(0V)$	$\Delta G^{\ddagger}(U^0)$	U^0 [a]	β' [b]	$\Delta G^{\ddagger}(0V)$
1a	Cu-DAN	$*CO+*CO+H^++e^- \rightarrow *COH–CO$	H-shuttling	0.79	1.07	–0.12	0.49	1.13
1b	Cu-SAN		H-shuttling	0.94	1.50	–0.25	0.49	1.63
2	Cu-DAN	$*COH–CO+ H^++e^- \rightarrow *COH–COH$	H-shuttling	0.24	0.76	–0.17	0.49	0.84
3a	Cu-DAN	$*COH–CO+ H^++e^- \rightarrow *C–CO + H_2O$	H-shuttling	–0.99	0.72	–0.21	0.49	0.82
3b	Cu-DAN		surface *H transfer	–1.25	1.66	–0.39	0.49	1.86
4a	Cu-DAN	$*COH–CO+ H^++e^- \rightarrow *COH–CHO$	surface *H transfer	–0.21	0.41	–0.10	0.49	0.45
4b	Cu(100)		surface *H transfer	–0.04	0.69	–0.05	0.49	0.72
5	Cu-DAN	$*COH–CHO+ H^++e^- \rightarrow *CHOH–CHO$	surface *H transfer	–0.38	0.52	–0.08	0.49	0.56
6	Cu-DAN	$*COH–CHO+ H^++e^- \rightarrow *C–CHO$	surface *H transfer	0.04	1.02	–0.11	0.49	1.07
7	Cu-DAN	$*CHOH–CHO+ H^++e^- \rightarrow *CH_2OH–CHO$	surface *H transfer	–0.27	0.25	0.04	0.49	0.23
8	Cu-DAN	$*CH_2OH–CHO+ H^++e^- \rightarrow *CH_2OH–CH_2O$	surface *H transfer	–0.29	0.39	–0.08	0.49	0.43
9	Cu-DAN	$*CH_2OH–CH_2O+ H^++e^- \rightarrow CH_2OH–CH_3+*O$	surface *H transfer	–0.58	0.98	–0.15	0.49	1.05
10	Cu-DAN	$*CH_2OH–CH_2O+ H^++e^- \rightarrow *CH_2OH–CH_2OH$	H-shuttling	–0.19	0.73	–0.18	0.49	0.82

[a] U^0 is the potential where reaction $*A+H^++e^- \rightarrow *A+*H$ possess zero free energy change i.e. $G(*A+*H) + eU^0 – G(*A) – 1/2H_2 = 0$, where *A means the system with A adsorbed on the surface.

[b] β' is the reaction symmetry factor, which is approximated as 0.49 for all elementary steps.[7]

Table S3. Atomic coordinates of initial state (IS), transition state (TS), and final state (FS) of Reaction 1a listed in Table S1.

Reaction 1a: *CO+*CO+ H^++e^- →*COH–CO on Cu-DAN via H-shuttling.		
IS	TS	FS
Cu C H O	Cu C H O	Cu C H O
1.00000000000000	1.00000000000000	1.00000000000000
12.8585004805000001 0.0000000000000000 0.0000000000000000	12.8585004805000001 0.0000000000000000 0.0000000000000000	12.8585004805000001 0.0000000000000000 0.0000000000000000
-6.4291002405000004 11.1357880710000003 0.0000000000000000	-6.4291002405000004 11.1357880710000003 0.0000000000000000	-6.4291002405000004 11.1357880710000003 0.0000000000000000
0.0000000000000000 0.0000000000000000 15.2994003295999992	0.0000000000000000 0.0000000000000000 15.2994003295999992	0.0000000000000000 0.0000000000000000 15.2994003295999992
Cu C H O	Cu C H O	Cu C H O
55 2 3 3	55 2 3 3	55 2 3 3
Selective dynamics	Selective dynamics	Selective dynamics
Direct	Direct	Direct
0.0000000000000000 0.0000000000000000 0.0000000000000000 F F F	0.0000000000000000 0.0000000000000000 0.0000000000000000 F F F	0.0000000000000000 0.0000000000000000 0.0000000000000000 F F F
0.0000000000000000 0.2000000000000028 0.0000000000000000 F F F	0.0000000000000000 0.2000000000000028 0.0000000000000000 F F F	0.0000000000000000 0.2000000000000028 0.0000000000000000 F F F
0.0000000000000000 0.3999999999999986 0.0000000000000000 F F F	0.0000000000000000 0.3 999999999999986 0.0000000000000000 F F F	0.0000000000000000 0.3999999999999986 0.0000000000000000 F F F
0.0000000000000000 0.6000000000000014 0.0000000000000000 F F F	0.0000000000000000 0.6000000000000014 0.0000000000000000 F F F	0.0000000000000000 0.6000000000000014 0.0000000000000000 F F F
0.0000000000000000 0.7999999999999972 0.0000000000000000 F F F	0.0000000000000000 0.7999999999999972 0.0000000000000000 F F F	0.0000000000000000 0.7999999999999972 0.0000000000000000 F F F
0.2000000000000028 0.0000000000000000 0.0000000000000000 F F F	0.2000000000000028 0.0000000000000000 0.0000000000000000 F F F	0.2000000000000028 0.0000000000000000 0.0000000000000000 F F F
0.2000000000000028 0.2000000000000028 0.0000000000000000 F F F	0.2000000000000028 0.2000000000000028 0.0000000000000000 F F F	0.2000000000000028 0.2000000000000028 0.0000000000000000 F F F
0.2000000000000028 0.3999999999999986 0.0000000000000000 F F F	0.2000000000000028 0.3999999999999986 0.0000000000000000 F F F	0.2000000000000028 0.3999999999999986 0.0000000000000000 F F F
0.2000000000000028 0.6000000000000014 0.0000000000000000 F F F	0.2000000000000028 0.6000000000000014 0.0000000000000000 F F F	0.2000000000000028 0.6000000000000014 0.0000000000000000 F F F
0.2000000000000028 0.7999999999999972 0.0000000000000000 F F F	0.2000000000000028 0.7999999999999972 0.0000000000000000 F F F	0.2000000000000028 0.7999999999999972 0.0000000000000000 F F F
0.3999999999999986 0.0000000000000000 0.0000000000000000 F F F	0.3999999999999986 0.0000000000000000 0.0000000000000000 F F F	0.3999999999999986 0.0000000000000000 0.0000000000000000 F F F
0.3999999999999986 0.2000000000000028 0.0000000000000000 F F F	0.3999999999999986 0.2000000000000028 0.0000000000000000 F F F	0.3999999999999986 0.2000000000000028 0.0000000000000000 F F F
0.3999999999999986 0.3999999999999986 0.0000000000000000 F F F	0.3999999999999986 0.3999999999999986 0.0000000000000000 F F F	0.3999999999999986 0.3999999999999986 0.0000000000000000 F F F
0.3999999999999986 0.6000000000000014 0.0000000000000000 F F F	0.3999999999999986 0.6000000000000014 0.0000000000000000 F F F	0.3999999999999986 0.6000000000000014 0.0000000000000000 F F F
0.3999999999999986 0.7999999999999972 0.0000000000000000 F F F	0.3999999999999986 0.7999999999999972 0.0000000000000000 F F F	0.3999999999999986 0.7999999999999972 0.0000000000000000 F F F
0.6000000000000014 0.0000000000000000 0.0000000000000000 F F F	0.6000000000000014 0.0000000000000000 0.0000000000000000 F F F	0.6000000000000014 0.0000000000000000 0.0000000000000000 F F F
0.6000000000000014 0.2000000000000028 0.0000000000000000 F F F	0.6000000000000014 0.2000000000000028 0.0000000000000000 F F F	0.6000000000000014 0.2000000000000028 0.0000000000000000 F F F
0.6000000000000014 0.3999999999999986 0.0000000000000000 F F F	0.6000000000000014 0.3999999999999986 0.0000000000000000 F F F	0.6000000000000014 0.3999999999999986 0.0000000000000000 F F F
0.6000000000000014 0.6000000000000014 0.0000000000000000 F F F	0.6000000000000014 0.6000000000000014 0.0000000000000000 F F F	0.6000000000000014 0.6000000000000014 0.0000000000000000 F F F
0.6000000000000014 0.7999999999999972 0.0000000000000000 F F F	0.6000000000000014 0.7999999999999972 0.0000000000000000 F F F	0.6000000000000014 0.7999999999999972 0.0000000000000000 F F F
0.7999999999999972 0.0000000000000000 0.0000000000000000 F F F	0.7999999999999972 0.0000000000000000 0.0000000000000000 F F F	0.7999999999999972 0.0000000000000000 0.0000000000000000 F F F
0.7999999999999972 0.2000000000000028 0.0000000000000000 F F F	0.7999999999999972 0.2000000000000028 0.0000000000000000 F F F	0.7999999999999972 0.2000000000000028 0.0000000000000000 F F F
0.7999999999999972 0.3999999999999986 0.0000000000000000 F F F	0.7999999999999972 0.3999999999999986 0.0000000000000000 F F F	0.7999999999999972 0.3999999999999986 0.0000000000000000 F F F
0.7999999999999972 0.6000000000000014 0.0000000000000000 F F F	0.7999999999999972 0.6000000000000014 0.0000000000000000 F F F	0.7999999999999972 0.6000000000000014 0.0000000000000000 F F F
0.7999999999999972 0.7999999999999972 0.0000000000000000 F F F	0.7999999999999972 0.7999999999999972 0.0000000000000000 F F F	0.7999999999999972 0.7999999999999972 0.0000000000000000 F F F
0.2763734059729984 0.9275805429559576 0.1323374930139236 T T T	0.2774940410672451 0.9280976607649216 0.1317780662736026 T T T	0.2759330261349316 0.9292308917795646 0.1317089167533536 T T T
0.4666644456031577 0.7333340047990546 0.1368027475005462 T T T	0.4666644456031577 0.7333340047990546 0.1368027475005462 T T T	0.4666644456031577 0.7333340047990546 0.1368027475005462 T T T
0.6666644456031534 0.5333340047990589 0.1368027475005462 T T T	0.6666644456031534 0.5333340047990589 0.1368027475005462 T T T	0.6666644456031534 0.5333340047990589 0.1368027475005462 T T T
0.8587517665096048 0.3369126022125444 0.1288542110224797 T T T	0.9107456377686554 0.3891761840436492 0.1452875898208144 T T T	0.9190442831622360 0.3937321265562653 0.1445233992367152 T T T
0.2673107969312974 0.7323697467352438 0.1328570400956452 T T T	0.2669076665179617 0.7337354542110339 0.1309960252533542 T T T	0.2666548723923730 0.7338547123876894 0.1303586864887370 T T T
0.4748398886885825 0.9371504415854010 0.1336454669735738 T T T	0.4772382711489767 0.9376764055425930 0.1333858270090803 T T T	0.4747502626958304 0.9378712991954916 0.1329048241918831 T T T
0.4666644456031577 0.5333340047990589 0.1368027475005462 T T T	0.4666644456031577 0.5333340047990589 0.1368027475005462 T T T	0.4666644456031577 0.5333340047990589 0.1368027475005462 T T T
0.6666644456031534 0.7333340047990546 0.1368027475005462 T T T	0.6666644456031534 0.7333340047990546 0.1368027475005462 T T T	0.6666644456031534 0.7333340047990546 0.1368027475005462 T T T
0.8646808727375266 0.5335379161241262 0.1330206632040215 T T T	0.8719818857330018 0.5513186033960692 0.1294535602581317 T T T	0.8701348516754888 0.5490595109827400 0.1312027634142850 T T T
0.6670901971597442 0.3376262796167326 0.1354501242003021 T T T	0.6823027579348296 0.3443873202718245 0.1336984272199554 T T T	0.6828311563827477 0.3435999011484749 0.1333104628216771 T T T
0.2659671450664912 0.5297374832318184 0.1326172965422326 T T T	0.2627767275792590 0.5266180113629346 0.1367424001198007 T T T	0.2580069375754813 0.5256869548378506 0.1342513334313770 T T T
0.4688531600558845 0.3377931505960579 0.1335124512974523 T T T	0.4692607333348094 0.3364758276350812 0.1328521695781531 T T T	0.4717352632819780 0.3377373782389445 0.1344536975241202 T T T
0.2652459217657827 0.3252916947539419 0.1326304864369880 T T T	0.2618428387581179 0.3200483205943768 0.1359906255681420 T T T	0.2683895676526589 0.3273145569517538 0.1302994779630633 T T T
0.6717486266915081 0.9401245602328060 0.1315022288117634 T T T	0.6746812928747101 0.9400692341397296 0.1309381516961471 T T T	0.6725805591362400 0.9409420603254508 0.1313276838781081 T T T
0.8637354127747792 0.7324007891150058 0.1344845768594131 T T T	0.8601807064837215 0.7386126104769808 0.1395123965514964 T T T	0.8610699750975009 0.7376523016365167 0.1381371911658770 T T T
0.8665442598648233 0.9320059744664064 0.1271837419818141 T T T	0.8713398506143269 0.9403682121830951 0.1299028999044375 T T T	0.8658018713769059 0.9342615579522536 0.1285389844395953 T T T
0.3540191320134473 0.8554934880420786 0.2650033421136150 T T T	0.3561299777248476 0.8531865478011363 0.2642911559234872 T T T	0.3514201686116833 0.8572239397585841 0.2636496285666115 T T T
0.5333313179997745 0.6666662410030355 0.2736054950010853 T T T	0.5333313179997745 0.6666662410030355 0.2736054950010853 T T T	0.5333313179997745 0.6666662410030355 0.2736054950010853 T T T
0.3188766647719964 0.6488187840672958 0.2687601250681162 T T T	0.2801378194322235 0.6382681734967898 0.2674058082643626 T T T	0.2984676526058692 0.6472542572947131 0.2653991764656990 T T T
0.5382631571133357 0.4669125775865266 0.2692881665299257 T T T	0.5500212162369112 0.4565748025006875 0.2682324730782887 T T T	0.5449003368391923 0.4650622281396075 0.2696067727817257 T T T
0.3351176546384318 0.4552150221002709 0.2606168721557106 T T T	0.3553732630591587 0.4394418261125406 0.2686349911220476 T T T	0.3443043674062445 0.4377012117476474 0.2669083987829295 T T T
0.5515924808258462 0.8692617558384113 0.2673685577364773 T T T	0.5592980096287755 0.8753736813472028 0.2652301638467532 T T T	0.5515341169015823 0.8731952918179514 0.2645371422653834 T T T
0.7352200185292200 0.6726063159845377 0.2710685609579087 T T T	0.7237763797173608 0.6639341384125518 0.2790180036275586 T T T	0.7271529433833688 0.6677975125838332 0.2769854931575866 T T T
0.7462346774873381 0.8729453308557734 0.2572214213435596 T T T	0.7470839185826691 0.8678297424612305 0.2609811284577673 T T T	0.7437262806691043 0.8703979729314869 0.2601624658391956 T T T
0.7367333643578425 0.4795598127212142 0.2660530765993048 T T T	0.7539293488264153 0.4857431965640973 0.2611047264921190 T T T	0.7449621791178439 0.4820572643970041 0.2622049230399967 T T T
0.4437388617199887 0.7520995584855686 0.3956250039526304 T T T	0.4250809449052425 0.7603753654113374 0.3808505316407349 T T T	0.4229596606100138 0.7734253254431469 0.3869251773955821 T T T
0.4011663657743640 0.5367304101343726 0.4011566015302212 T T T	0.3269856612559799 0.5389543335899524 0.3903693147921470 T T T	0.3528528885633301 0.5570258723382353 0.3893890333310938 T T T
0.6515543550239720 0.7961079887994450 0.3931962634000917 T T T	0.6334340319938657 0.7885306556989164 0.3952845826238620 T T T	0.6286996969401791 0.8000716509867355 0.3932370098061388 T T T
0.6130266235387344 0.5891649929695767 0.4020872971111915 T T T	0.5377019471394135 0.5596380945257142 0.3981811244362828 T T T	0.5614693328128721 0.5798427271205151 0.4032444899061187 T T T
0.5261346846866674 0.6728481536628044 0.5099049131585643 T T T	0.4714711369717424 0.6624165318745637 0.4983866899032818 T T T	0.4831651937079132 0.6772469999270274 0.5025021802926787 T T T
0.2709046029175053 0.3751160441741725 0.3699373039390935 T T T	0.1974724511719206 0.4169857562681114 0.3133159876282083 T T T	0.2036226109258781 0.4437936300455465 0.3226087406267485 T T T
0.1707288893422144 0.5710322659738197 0.3239301645063316 T T T	0.1487678283928907 0.4618144575424958 0.2467042156132055 T T T	0.1568421647179248 0.4688717419132175 0.2447163349009794 T T T
0.1378896696683089 0.1647357361323532 0.2637395175544845 T T T	0.0644823250848737 0.2564801728847045 0.3042927860928553 T T T	0.0402129468101246 0.3405310331728101 0.3525643085812615 T T T
0.0789184999077361 0.1066076324611441 0.1725876699338647 T T T	0.1358092741634129 0.1229270633089940 0.1960307479592454 T T T	0.1022005801524051 0.1088437143457875 0.1754540610427815 T T T
0.1343322016134791 0.4675219092665182 0.0619676134352680 T T T	0.1222540560123422 0.2563360978012678 0.1486800590235611 T T T	0.0496737686410891 0.1923185775042381 0.1906097238264829 T T T
0.1223213247497480 0.1830284426468037 0.2048172445445267 T T T	0.1182104312546736 0.1858927512169251 0.1794875275188736 T T T	0.1208990886922510 0.1854463305528166 0.2030307059661308 T T T
0.0750432236247554 0.5203415532732601 0.3534734654023226 T T T	0.0359435015453488 0.4065354029343360 0.2294403723717657 T T T	0.0405684917239722 0.4097960821227892 0.2326101652874483 T T T
0.2054810037805627 0.2816122894957866 0.4024585066221745 T T T	0.1227808175756999 0.3074926090646437 0.3496004291861500 T T T	0.1189878580029992 0.3643200633339206 0.3798766154856253 T T T

Table S4. Atomic coordinates of IS, TS, and FS of Reaction 1b listed in Table S1.

Reaction 1b: *CO+*CO+ H^++e^-→*COH–CO on Cu-SAN via H-shuttling		
IS	TS	FS
Cu C H O<br>1.00000000000000<br>12.8585004805000001 0.0000000000000000 0.0000000000000000<br>-6.4291002405000004 11.1357880710000003 0.0000000000000000<br>0.0000000000000000 0.0000000000000000 15.2994003295999992<br>Cu C H O<br>55 2 3 3<br>Selective dynamics<br>Direct<br>0.0000000000000000 0.0000000000000000 0.0000000000000000 F F F<br>0.0000000000000000 0.2000000000000028 0.0000000000000000 F F F<br>0.0000000000000000 0.3999999999999986 0.0000000000000000 F F F<br>0.0000000000000000 0.6000000000000014 0.0000000000000000 F F F<br>0.0000000000000000 0.7999999999999972 0.0000000000000000 F F F<br>0.2000000000000028 0.0000000000000000 0.0000000000000000 F F F<br>0.2000000000000028 0.2000000000000028 0.0000000000000000 F F F<br>0.2000000000000028 0.3999999999999986 0.0000000000000000 F F F<br>0.2000000000000028 0.6000000000000014 0.0000000000000000 F F F<br>0.2000000000000028 0.7999999999999972 0.0000000000000000 F F F<br>0.3999999999999986 0.0000000000000000 0.0000000000000000 F F F<br>0.3999999999999986 0.2000000000000028 0.0000000000000000 F F F<br>0.3999999999999986 0.3999999999999986 0.0000000000000000 F F F<br>0.3999999999999986 0.6000000000000014 0.0000000000000000 F F F<br>0.3999999999999986 0.7999999999999972 0.0000000000000000 F F F<br>0.6000000000000014 0.0000000000000000 0.0000000000000000 F F F<br>0.6000000000000014 0.2000000000000028 0.0000000000000000 F F F<br>0.6000000000000014 0.3999999999999986 0.0000000000000000 F F F<br>0.6000000000000014 0.6000000000000014 0.0000000000000000 F F F<br>0.6000000000000014 0.7999999999999972 0.0000000000000000 F F F<br>0.7999999999999972 0.0000000000000000 0.0000000000000000 F F F<br>0.7999999999999972 0.2000000000000028 0.0000000000000000 F F F<br>0.7999999999999972 0.3999999999999986 0.0000000000000000 F F F<br>0.7999999999999972 0.6000000000000014 0.0000000000000000 F F F<br>0.7999999999999972 0.7999999999999972 0.0000000000000000 F F F<br>0.2781713742405466 0.9232736646864270 0.1334675070203859 T T T<br>0.4666644456031577 0.7333340047990546 0.1368027475005462 T T T<br>0.6666644456031534 0.5333340047990589 0.1368027475005462 T T T<br>0.8608168405116802 0.3383705232036037 0.1287910777447995 T T T<br>0.2675101587519791 0.7263712996367083 0.1347911413219611 T T T<br>0.4761965227769058 0.9349936774286948 0.1340783519541450 T T T<br>0.4666644456031577 0.5333340047990589 0.1368027475005462 T T T<br>0.6666644456031534 0.7333340047990546 0.1368027475005462 T T T<br>0.8641592565059918 0.5333395873127199 0.1335969653093571 T T T<br>0.6664040774298703 0.3358425977692778 0.1340350886498072 T T T<br>0.2622036449511278 0.5270552565275325 0.1302665396273993 T T T<br>0.4676335346565644 0.3377508709548033 0.1357100120603911 T T T<br>0.2689071366844191 0.3323478895934193 0.1273298656835757 T T T<br>0.6758113681043060 0.9409436774728084 0.1303837583481261 T T T<br>0.8622025491442600 0.7319480281249270 0.1360032930494933 T T T<br>0.8689163491742296 0.9323602815516367 0.1277731327676248 T T T<br>0.3546779915154790 0.8522543805573838 0.2664375071382198 T T T<br>0.5333313179997745 0.6666662410030355 0.2736054950010853 T T T<br>0.2877702311233457 0.6189134530057901 0.2765927082067832 T T T<br>0.5354703453960126 0.4675507384435624 0.2714435750750320 T T T<br>0.3322166902066265 0.4512834466394971 0.2587668366348156 T T T<br>0.5610041677233826 0.8737154036495405 0.2633865062806024 T T T<br>0.7302927778672698 0.6654997869311104 0.2727490056026862 T T T<br>0.7507714232546595 0.8696997129202274 0.2594441956214128 T T T<br>0.7309417466198633 0.4724353273373051 0.2645927617712177 T T T<br>0.4328247684431472 0.7738068615299414 0.3894450441840109 T T T<br>0.3901604978335294 0.5593636570633219 0.3954084632211706 T T T<br>0.6374274701739334 0.8058077410085771 0.3921455434060183 T T T<br>0.5971732095834336 0.5958215679353642 0.4048500405645403 T T T<br>0.5109911345472963 0.6876618591752239 0.5085602966892898 T T T<br>0.3502966453364271 0.8926564632371545 0.3834581858764686 T T T<br>0.1575021777794805 0.6177633214096356 0.3305940146070169 T T T<br>0.9948901367792010 0.3149562186678643 0.4721764104446685 T T T<br>0.0117950291673530 0.4202815925667958 0.5298277171492181 T T T<br>0.2621911864578094 0.4815688409318894 0.3369618523570627 T T T<br>0.0322244313096630 0.3570798390862418 0.5261692711456819 T T T<br>0.0756956245153165 0.6133076455880032 0.3658885271834095 T T T<br>0.3389762906524047 0.9308740666329433 0.4498159328679205 T T T	Cu C H O<br>1.00000000000000<br>12.8585004805000001 0.0000000000000000 0.0000000000000000<br>-6.4291002405000004 11.1357880710000003 0.0000000000000000<br>0.0000000000000000 0.0000000000000000 15.2994003295999992<br>Cu C H O<br>55 2 3 3<br>Selective dynamics<br>Direct<br>0.0000000000000000 0.0000000000000000 0.0000000000000000 F F F<br>0.0000000000000000 0.2000000000000028 0.0000000000000000 F F F<br>0.0000000000000000 0.3999999999999986 0.0000000000000000 F F F<br>0.0000000000000000 0.6000000000000014 0.0000000000000000 F F F<br>0.0000000000000000 0.7999999999999972 0.0000000000000000 F F F<br>0.2000000000000028 0.0000000000000000 0.0000000000000000 F F F<br>0.2000000000000028 0.2000000000000028 0.0000000000000000 F F F<br>0.2000000000000028 0.3999999999999986 0.0000000000000000 F F F<br>0.2000000000000028 0.6000000000000014 0.0000000000000000 F F F<br>0.2000000000000028 0.7999999999999972 0.0000000000000000 F F F<br>0.3999999999999986 0.0000000000000000 0.0000000000000000 F F F<br>0.3999999999999986 0.2000000000000028 0.0000000000000000 F F F<br>0.3999999999999986 0.3999999999999986 0.0000000000000000 F F F<br>0.3999999999999986 0.6000000000000014 0.0000000000000000 F F F<br>0.3999999999999986 0.7999999999999972 0.0000000000000000 F F F<br>0.6000000000000014 0.0000000000000000 0.0000000000000000 F F F<br>0.6000000000000014 0.2000000000000028 0.0000000000000000 F F F<br>0.6000000000000014 0.3999999999999986 0.0000000000000000 F F F<br>0.6000000000000014 0.6000000000000014 0.0000000000000000 F F F<br>0.6000000000000014 0.7999999999999972 0.0000000000000000 F F F<br>0.7999999999999972 0.0000000000000000 0.0000000000000000 F F F<br>0.7999999999999972 0.2000000000000028 0.0000000000000000 F F F<br>0.7999999999999972 0.3999999999999986 0.0000000000000000 F F F<br>0.7999999999999972 0.6000000000000014 0.0000000000000000 F F F<br>0.7999999999999972 0.7999999999999972 0.0000000000000000 F F F<br>0.2783873916485336 0.9275281115388972 0.1281277979589293 T T T<br>0.4645692318962702 0.7323670548956187 0.1354057277978384 T T T<br>0.6662002356971897 0.5338169553963326 0.1358095665998107 T T T<br>0.8614943482297626 0.3375885859910882 0.1259431139605189 T T T<br>0.2647073752363598 0.7287292205703846 0.1294622219763576 T T T<br>0.4780965449700690 0.9368078844213678 0.1295463279222082 T T T<br>0.4657270387003507 0.5320977479026254 0.1346313791995470 T T T<br>0.6657510079991198 0.7327853666011066 0.1349863735969024 T T T<br>0.8664329469018256 0.5332869701675668 0.1290283040895703 T T T<br>0.6667612273759520 0.3345429816646794 0.1300800898255548 T T T<br>0.2607430715802793 0.5247128770550800 0.1283930810090224 T T T<br>0.4673909960366869 0.3331494006990769 0.1291942303696207 T T T<br>0.2711834484776260 0.3349396910449310 0.1270883766527399 T T T<br>0.6763060332669681 0.9413661619574479 0.1281313314783594 T T T<br>0.8611589073832124 0.7309979628830492 0.1331216668749192 T T T<br>0.8666113672291268 0.9311479124514984 0.1243083134367012 T T T<br>0.3567881048269144 0.8612401320109135 0.2599398067120629 T T T<br>0.5296525373004926 0.6640206900000720 0.2689701485971625 T T T<br>0.3044046074378170 0.6398325027807998 0.2602307093677210 T T T<br>0.5326928650501128 0.4591883111431778 0.2651453474565855 T T T<br>0.3387914807791660 0.4595143797966413 0.2657134204713398 T T T<br>0.5616469221640331 0.8772745282487266 0.2622723185162716 T T T<br>0.7205881888802885 0.6614023225002197 0.2744889594999732 T T T<br>0.7496633050788375 0.8702296112120280 0.2579945027244480 T T T<br>0.7350269814763948 0.4725272336872526 0.2589192882804509 T T T<br>0.4460956362498132 0.8030086568117158 0.3977108125654101 T T T<br>0.3645090220552314 0.5862964967468515 0.3928438498553001 T T T<br>0.6335379945814071 0.7957473383677766 0.3894484287774286 T T T<br>0.5439150305360235 0.5670119758342237 0.3995952015073908 T T T<br>0.5043432079384352 0.6873227519553563 0.5079265701981914 T T T<br>0.3070746502358982 0.8342810541658814 0.3801915158326334 T T T<br>0.2564968387795805 0.6602739253742683 0.3786313093800443 T T T<br>0.1178297339510612 0.5250643289614290 0.4356620215454704 T T T<br>0.9926619666032525 0.3322083320773632 0.4451014973126790 T T T<br>0.1285087395850572 0.3707498066926874 0.4378409539475828 T T T<br>0.0727620715414432 0.3927682764322704 0.4640718532483881 T T T<br>0.1565130895185713 0.6153211778889837 0.4231926653263935 T T T<br>0.2572498024076048 0.8617803921903634 0.4333807013306434 T T T	Cu C H O<br>1.00000000000000<br>12.8585004805000001 0.0000000000000000 0.0000000000000000<br>-6.4291002405000004 11.1357880710000003 0.0000000000000000<br>0.0000000000000000 0.0000000000000000 15.2994003295999992<br>Cu C H O<br>55 2 3 3<br>Selective dynamics<br>Direct<br>0.0000000000000000 0.0000000000000000 0.0000000000000000 F F F<br>0.0000000000000000 0.2000000000000028 0.0000000000000000 F F F<br>0.0000000000000000 0.3999999999999986 0.0000000000000000 F F F<br>0.0000000000000000 0.6000000000000014 0.0000000000000000 F F F<br>0.0000000000000000 0.7999999999999972 0.0000000000000000 F F F<br>0.2000000000000028 0.0000000000000000 0.0000000000000000 F F F<br>0.2000000000000028 0.2000000000000028 0.0000000000000000 F F F<br>0.2000000000000028 0.3999999999999986 0.0000000000000000 F F F<br>0.2000000000000028 0.6000000000000014 0.0000000000000000 F F F<br>0.2000000000000028 0.7999999999999972 0.0000000000000000 F F F<br>0.3999999999999986 0.0000000000000000 0.0000000000000000 F F F<br>0.3999999999999986 0.2000000000000028 0.0000000000000000 F F F<br>0.3999999999999986 0.3999999999999986 0.0000000000000000 F F F<br>0.3999999999999986 0.6000000000000014 0.0000000000000000 F F F<br>0.3999999999999986 0.7999999999999972 0.0000000000000000 F F F<br>0.6000000000000014 0.0000000000000000 0.0000000000000000 F F F<br>0.6000000000000014 0.2000000000000028 0.0000000000000000 F F F<br>0.6000000000000014 0.3999999999999986 0.0000000000000000 F F F<br>0.6000000000000014 0.6000000000000014 0.0000000000000000 F F F<br>0.6000000000000014 0.7999999999999972 0.0000000000000000 F F F<br>0.7999999999999972 0.0000000000000000 0.0000000000000000 F F F<br>0.7999999999999972 0.2000000000000028 0.0000000000000000 F F F<br>0.7999999999999972 0.3999999999999986 0.0000000000000000 F F F<br>0.7999999999999972 0.6000000000000014 0.0000000000000000 F F F<br>0.7999999999999972 0.7999999999999972 0.0000000000000000 F F F<br>0.2791554626737874 0.9271617504996736 0.1325692200874938 T T T<br>0.4629978217000001 0.7316418424999966 0.1343579629999994 T T T<br>0.6658520781999968 0.5341791682999997 0.1350646809000011 T T T<br>0.8612533056452588 0.3400411119091570 0.1289789960082511 T T T<br>0.2638780217162340 0.7286711196464688 0.1333415773895474 T T T<br>0.4756963312307834 0.9365787896217104 0.1342596486729646 T T T<br>0.4650239835999983 0.5311705551999992 0.1330028530000007 T T T<br>0.6650659298000008 0.7323738879000032 0.1336240931000034 T T T<br>0.8669008927108031 0.5358006293217177 0.1321842064057241 T T T<br>0.6673093272410257 0.3375933696474878 0.1346265931703926 T T T<br>0.2625139712363505 0.5262575409971320 0.1309940157198037 T T T<br>0.4665512971025032 0.3325004612184439 0.1316402639515181 T T T<br>0.2697878367673198 0.3351702921777122 0.1320653906482623 T T T<br>0.6737187119256833 0.9390688078856624 0.1306109870118736 T T T<br>0.8599938610757893 0.7314435265328054 0.1382410162763697 T T T<br>0.8689165231372130 0.9328252369087676 0.1275099162905208 T T T<br>0.3563975324814424 0.8532483625193893 0.2687254563242980 T T T<br>0.5268934517999995 0.6620365268000015 0.2654936388000024 T T T<br>0.3011471294477961 0.6375709591416200 0.2636805931066236 T T T<br>0.5327234443271590 0.4571442296816284 0.2646404400488176 T T T<br>0.3411157017640520 0.4628012432476342 0.2673955950278046 T T T<br>0.5581121420201568 0.8727260335752981 0.2651624392487927 T T T<br>0.7180449979782720 0.6603559098522268 0.2769927930456657 T T T<br>0.7474097807438934 0.8674448149942438 0.2569134880340316 T T T<br>0.7368059863854634 0.4774864429669193 0.2621422235730532 T T T<br>0.4655281122060988 0.8209172660383157 0.4078030398043653 T T T<br>0.3686389184751044 0.5984607700535707 0.3978216401318439 T T T<br>0.6373393903430410 0.7863056091284745 0.3882490064518874 T T T<br>0.5356517432955212 0.5615413491809571 0.3973590610078179 T T T<br>0.5135313462457620 0.6901583548485293 0.5106571314273624 T T T<br>0.2954057906979471 0.7861644422126015 0.3925887121963051 T T T<br>0.2469803934712830 0.6549857978734962 0.3849587192991351 T T T<br>0.1104682528824686 0.5038108215020743 0.4274641637754976 T T T<br>0.9828423867862420 0.2863248866269069 0.4640024924788302 T T T<br>0.0010831920880507 0.3778656667380190 0.5367683234185990 T T T<br>0.0412273605829685 0.3640668916733043 0.4879188581374252 T T T<br>0.1361193241016149 0.5901831120039787 0.4212226351954583 T T T<br>0.2488641751469659 0.8410265269215254 0.4255409914963029 T T T

Table S5. Atomic coordinates of IS, TS, and FS of Reaction 2a listed in Table S1.

Reaction 2: *CO-COH+ $H^{+}+e^{-}$→*COH–COH on Cu-DAN via H-shuttling.

IS	TS	FS
Cu C H O	Cu C H O	Cu C H O
1.00000000000000	1.00000000000000	1.00000000000000
12.8585004805000001 0.0000000000000000 0.0000000000000000	12.8585004805000001 0.0000000000000000 0.0000000000000000	12.8585004805000001 0.0000000000000000 0.0000000000000000
-6.4291002405000004 11.1357880710000003 0.0000000000000000	-6.4291002405000004 11.1357880710000003 0.0000000000000000	-6.4291002405000004 11.1357880710000003 0.0000000000000000
0.0000000000000000 0.0000000000000000 15.2994003295999992	0.0000000000000000 0.0000000000000000 15.2994003295999992	0.0000000000000000 0.0000000000000000 15.2994003295999992
Cu C H O	Cu C H O	Cu C H O
55 2 4 3	55 2 4 3	55 2 4 3
Selective dynamics	Selective dynamics	Selective dynamics
Direct	Direct	Direct
0.0000000000000000 0.0000000000000000 0.0000000000000000 F F F	0.0000000000000000 0.0000000000000000 0.0000000000000000 F F F	0.0000000000000000 0.0000000000000000 0.0000000000000000 F F F
0.0000000000000000 0.2000000000000028 0.0000000000000000 F F F	0.0000000000000000 0.2000000000000028 0.0000000000000000 F F F	0.0000000000000000 0.2000000000000028 0.0000000000000000 F F F
0.0000000000000000 0.3999999999999986 0.0000000000000000 F F F	0.0000000000000000 0.3999999999999986 0.0000000000000000 F F F	0.0000000000000000 0.3999999999999986 0.0000000000000000 F F F
0.0000000000000000 0.6000000000000014 0.0000000000000000 F F F	0.0000000000000000 0.6000000000000014 0.0000000000000000 F F F	0.0000000000000000 0.6000000000000014 0.0000000000000000 F F F
0.0000000000000000 0.7999999999999972 0.0000000000000000 F F F	0.0000000000000000 0.7999999999999972 0.0000000000000000 F F F	0.0000000000000000 0.7999999999999972 0.0000000000000000 F F F
0.2000000000000028 0.0000000000000000 0.0000000000000000 F F F	0.2000000000000028 0.0000000000000000 0.0000000000000000 F F F	0.2000000000000028 0.0000000000000000 0.0000000000000000 F F F
0.2000000000000028 0.2000000000000028 0.0000000000000000 F F F	0.2000000000000028 0.2000000000000028 0.0000000000000000 F F F	0.2000000000000028 0.2000000000000028 0.0000000000000000 F F F
0.2000000000000028 0.3999999999999986 0.0000000000000000 F F F	0.2000000000000028 0.3999999999999986 0.0000000000000000 F F F	0.2000000000000028 0.3999999999999986 0.0000000000000000 F F F
0.2000000000000028 0.6000000000000014 0.0000000000000000 F F F	0.2000000000000028 0.6000000000000014 0.0000000000000000 F F F	0.2000000000000028 0.6000000000000014 0.0000000000000000 F F F
0.2000000000000028 0.7999999999999972 0.0000000000000000 F F F	0.2000000000000028 0.7999999999999972 0.0000000000000000 F F F	0.2000000000000028 0.7999999999999972 0.0000000000000000 F F F
0.3999999999999986 0.0000000000000000 0.0000000000000000 F F F	0.3999999999999986 0.0000000000000000 0.0000000000000000 F F F	0.3999999999999986 0.0000000000000000 0.0000000000000000 F F F
0.3999999999999986 0.2000000000000028 0.0000000000000000 F F F	0.3999999999999986 0.2000000000000028 0.0000000000000000 F F F	0.3999999999999986 0.2000000000000028 0.0000000000000000 F F F
0.3999999999999986 0.3999999999999986 0.0000000000000000 F F F	0.3999999999999986 0.3999999999999986 0.0000000000000000 F F F	0.3999999999999986 0.3999999999999986 0.0000000000000000 F F F
0.3999999999999986 0.6000000000000014 0.0000000000000000 F F F	0.3999999999999986 0.6000000000000014 0.0000000000000000 F F F	0.3999999999999986 0.6000000000000014 0.0000000000000000 F F F
0.3999999999999986 0.7999999999999972 0.0000000000000000 F F F	0.3999999999999986 0.7999999999999972 0.0000000000000000 F F F	0.3999999999999986 0.7999999999999972 0.0000000000000000 F F F
0.6000000000000014 0.0000000000000000 0.0000000000000000 F F F	0.6000000000000014 0.0000000000000000 0.0000000000000000 F F F	0.6000000000000014 0.0000000000000000 0.0000000000000000 F F F
0.6000000000000014 0.2000000000000028 0.0000000000000000 F F F	0.6000000000000014 0.2000000000000028 0.0000000000000000 F F F	0.6000000000000014 0.2000000000000028 0.0000000000000000 F F F
0.6000000000000014 0.3999999999999986 0.0000000000000000 F F F	0.6000000000000014 0.3999999999999986 0.0000000000000000 F F F	0.6000000000000014 0.3999999999999986 0.0000000000000000 F F F
0.6000000000000014 0.6000000000000014 0.0000000000000000 F F F	0.6000000000000014 0.6000000000000014 0.0000000000000000 F F F	0.6000000000000014 0.6000000000000014 0.0000000000000000 F F F
0.6000000000000014 0.7999999999999972 0.0000000000000000 F F F	0.6000000000000014 0.7999999999999972 0.0000000000000000 F F F	0.6000000000000014 0.7999999999999972 0.0000000000000000 F F F
0.7999999999999972 0.0000000000000000 0.0000000000000000 F F F	0.7999999999999972 0.0000000000000000 0.0000000000000000 F F F	0.7999999999999972 0.0000000000000000 0.0000000000000000 F F F
0.7999999999999972 0.2000000000000028 0.0000000000000000 F F F	0.7999999999999972 0.2000000000000028 0.0000000000000000 F F F	0.7999999999999972 0.2000000000000028 0.0000000000000000 F F F
0.7999999999999972 0.3999999999999986 0.0000000000000000 F F F	0.7999999999999972 0.3999999999999986 0.0000000000000000 F F F	0.7999999999999972 0.3999999999999986 0.0000000000000000 F F F
0.7999999999999972 0.6000000000000014 0.0000000000000000 F F F	0.7999999999999972 0.6000000000000014 0.0000000000000000 F F F	0.7999999999999972 0.6000000000000014 0.0000000000000000 F F F
0.7999999999999972 0.7999999999999972 0.0000000000000000 F F F	0.7999999999999972 0.7999999999999972 0.0000000000000000 F F F	0.7999999999999972 0.7999999999999972 0.0000000000000000 F F F
0.2835502922722780 0.9342747891772436 0.1319146728921217 T T T	0.2779455656897309 0.9295960235720864 0.1313142688210651 T T T	0.2775500050716046 0.9303139179094220 0.1326916034017981 T T T
0.4666644456187115 0.7333340047990546 0.1368027474874722 T T T	0.4666644456098155 0.7333340047990546 0.1368027474949471 T T T	0.4666644456031577 0.7333340047990546 0.1368027475005462 T T T
0.6666644456187072 0.5333340047990589 0.1368027474874722 T T T	0.6666644456098112 0.5333340047990589 0.1368027474949471 T T T	0.6666644456031534 0.5333340047990589 0.1368027475005462 T T T
0.9136136382241256 0.3860781389677790 0.1434650844879624 T T T	0.8724444846941888 0.3491438192389205 0.1319717726102385 T T T	0.8630007317752241 0.3408958891126946 0.1298305367030093 T T T
0.2562335090357815 0.7284156988889460 0.1332433033892266 T T T	0.2635151243749909 0.7323790592435730 0.1318317351849264 T T T	0.2693676970648352 0.7366196943778275 0.1306997882781857 T T T
0.4758481046697432 0.9355328846303884 0.1332868994508577 T T T	0.4776402902196143 0.9394419802653150 0.1339479250019352 T T T	0.4769941652552201 0.9389523224533096 0.1329389971400161 T T T
0.4666644456187115 0.5333340047990589 0.1368027474874722 T T T	0.4666644456098155 0.5333340047990589 0.1368027474949471 T T T	0.4666644456031577 0.5333340047990589 0.1368027475005462 T T T
0.6666644456187072 0.7333340047990546 0.1368027474874722 T T T	0.6666644456098112 0.7333340047990546 0.1368027474949471 T T T	0.6666644456031534 0.7333340047990546 0.1368027475005462 T T T
0.8641457753245826 0.5423124521639792 0.1294354325990739 T T T	0.8685578260115528 0.5379945479432033 0.1274368038962395 T T T	0.8672946208409289 0.5355451576020245 0.1280545827415542 T T T
0.6778934104712169 0.3393174330706111 0.1328922161884148 T T T	0.6707719542425775 0.3402707231930604 0.1358441947905093 T T T	0.6672328164292048 0.3383098757276715 0.1352077831132083 T T T
0.2577558360131925 0.5249245762240896 0.1337967542621427 T T T	0.2551701275493903 0.5236335584084898 0.1334786092455027 T T T	0.2549370537992985 0.5253884487477735 0.1374106826332533 T T T
0.4666868242519626 0.3363107069761908 0.1354591639721820 T T T	0.4646096539117688 0.3362373644834863 0.1360486250710114 T T T	0.4635984560227422 0.3364319878776870 0.1359923875959986 T T T
0.2660059241603797 0.3294905368891279 0.1241126730267999 T T T	0.2655580435582382 0.3313228667977165 0.1259230082635669 T T T	0.2657963278267210 0.3319501139633022 0.1254726353937373 T T T
0.6751160710588726 0.9398987043257256 0.1302285815846208 T T T	0.6736219596012564 0.9406921078031253 0.1317007387293935 T T T	0.6739621380783565 0.9408547923033490 0.1318082958277499 T T T
0.8613753602157161 0.7350931323516167 0.1367776748547810 T T T	0.8611209008010134 0.7312280408313447 0.1369082085708132 T T T	0.8607009195042434 0.7311604362123854 0.1366844684035086 T T T
0.8678697105792029 0.9341158001817512 0.1280052400387505 T T T	0.8658332425606766 0.9314835424371285 0.1281676803004135 T T T	0.8663088942032635 0.9312754386397296 0.1281374507559500 T T T
0.3577600288954836 0.8535702620914803 0.2638304255268804 T T T	0.3500244482020876 0.8479905746065853 0.2634200585805256 T T T	0.3562380773407067 0.8605866555724276 0.2652281687232057 T T T
0.5333313180231087 0.6666662410030355 0.2736054949749445 T T T	0.5333313180097790 0.6666662410030355 0.2736054949898943 T T T	0.5333313179997745 0.6666662410030355 0.2736054950010853 T T T
0.3020759451455687 0.6405257097111191 0.2685269376990197 T T T	0.3132359929670718 0.6454371477716326 0.2625746855299592 T T T	0.3194814679040059 0.6564779088817901 0.2640512622929446 T T T
0.5453286065120457 0.4636308358708069 0.2689065113254756 T T T	0.5580063369420402 0.4566956793750671 0.2698404790882278 T T T	0.5551653960604628 0.4589077260567607 0.2696546190196086 T T T
0.3387047774911725 0.4156755497522417 0.2620817394175483 T T T	0.3538096990481284 0.4072639257823029 0.2655943950342262 T T T	0.3519137247435537 0.4117970128219803 0.2651912124528561 T T T
0.5626595788420253 0.8770658925817201 0.2647468309001976 T T T	0.5542932067354300 0.8727024378495978 0.2658034674527213 T T T	0.5526237748648817 0.8704368508761507 0.2652055782253372 T T T
0.7287511528164691 0.6662386056414704 0.2758859215898822 T T T	0.7280832730829411 0.6682512877707601 0.2769403785715870 T T T	0.7293560439890621 0.6681914538530680 0.2766745517750628 T T T
0.7488481359246552 0.8697778566060876 0.2604410210721365 T T T	0.7455037383526721 0.8706058746161622 0.2605571749617737 T T T	0.7453611353173032 0.8700648564978183 0.2601941256637296 T T T
0.7433386803634637 0.4784198409643872 0.2620719113375907 T T T	0.7618642086301018 0.4933270176905362 0.2618537343647314 T T T	0.7546967494084782 0.4858255589942364 0.2615486101522623 T T T
0.4311407404758031 0.7635837125944975 0.3860952219541567 T T T	0.4295362879553933 0.7598493261944923 0.3871416774204932 T T T	0.4320561159744617 0.7666200060248543 0.3902385260215490 T T T
0.3575006803438855 0.5377258137531022 0.3843006990163961 T T T	0.3553152493296443 0.5370194205531169 0.3812740196989704 T T T	0.3574360238493586 0.5423879207060736 0.3819299163764152 T T T
0.6365037421000918 0.7989653989205500 0.3937687017705365 T T T	0.6370413361810955 0.7928890525261297 0.3947662731363703 T T T	0.6379014972504718 0.7943269323535470 0.3945705193136908 T T T
0.5738010246767696 0.5795554274850822 0.4011280088441613 T T T	0.5678632268589742 0.5714950195412650 0.3984533093248877 T T T	0.5695522987486519 0.5743148382348380 0.3991200959121098 T T T
0.4855227558485977 0.6647637134214995 0.4984617777439444 T T T	0.4830381123642439 0.6576074316016135 0.4982609426706787 T T T	0.4852580757185387 0.6613046616518690 0.4990418442552703 T T T
0.2024434792603950 0.4273295416347467 0.3153335902283774 T T T	0.2119245464882060 0.4084635171636491 0.3107137293471379 T T T	0.2110930012744050 0.4124854214112594 0.3117034838271494 T T T
0.1580906540985562 0.4655192038783846 0.2430408514807233 T T T	0.1714986820070377 0.4734801447383217 0.2552915437486925 T T T	0.1805237963426727 0.4901859204576344 0.2660755730053772 T T T
0.0397555716120231 0.3371257039511362 0.3508369397944492 T T T	0.0497313042405485 0.3219724448128812 0.3473127112246813 T T T	0.0493607536379949 0.3244479493615915 0.3522875764199763 T T T
0.0571045643292034 0.5865855822376161 0.2386268995324084 T T T	0.0578366140322912 0.5679263497869494 0.2682001659844938 T T T	0.0527961617522649 0.5318705642457534 0.2699043948444215 T T T
0.0328742704738582 0.6889942618238053 0.2074936753679775 T T T	0.0228619602304507 0.6662450617955663 0.2369347852564271 T T T	0.9983381883586280 0.6723388401265855 0.2499338703092511 T T T
0.3028879969680451 0.7496614238664289 0.3456536897162604 T T T	0.1943722072250951 0.7236918845637723 0.2486868614852565 T T T	0.1344662571912320 0.7241939169072963 0.2723928301314573 T T T
0.1176840759660155 0.3508890625754952 0.3739940694702909 T T T	0.1248100463613042 0.3217329226212039 0.3620670736294938 T T T	0.1233855612120618 0.3197518515299390 0.3587732268159530 T T T
0.0415455012628862 0.4248852794336532 0.2376432356656975 T T T	0.0595383060380262 0.4446526600368031 0.2578101016258156 T T T	0.0620884505032217 0.4584308543935757 0.2733050009380276 T T T
0.0956302343597504 0.6748940771351921 0.2322907042597538 T T T	0.0741993084292500 0.6539812068256405 0.2789980266785403 T T T	0.0526646557553851 0.6603494023167612 0.2888136207561173 T T T

Table S6. Atomic coordinates of IS, TS, and FS of Reaction 3a listed in Table S1.

Reaction 3a: *COH–CO+ H^{+}+e^{-}→*C–CO + H_2O on Cu-DAN via H-shuttling

IS	TS	FS
Cu C H O	Cu C H O	Cu C H O
1.00000000000000	1.00000000000000	1.00000000000000
12.8585004805000001 0.0000000000000000 0.0000000000000000	12.8585004805000001 0.0000000000000000 0.0000000000000000	12.8585004805000001 0.0000000000000000 0.0000000000000000
-6.4291002405000004 11.1357880710000003 0.0000000000000000	-6.4291002405000004 11.1357880710000003 0.0000000000000000	-6.4291002405000004 11.1357880710000003 0.0000000000000000
0.0000000000000000 0.0000000000000000 15.2994003295999992	0.0000000000000000 0.0000000000000000 15.2994003295999992	0.0000000000000000 0.0000000000000000 15.2994003295999992
Cu C H O	Cu C H O	Cu C H O
55 2 4 3	55 2 4 3	55 2 4 3
Selective dynamics	Selective dynamics	Selective dynamics
Direct	Direct	Direct
0.0000000000000000 0.0000000000000000 0.0000000000000000 F F F	0.0000000000000000 0.0000000000000000 0.0000000000000000 F F F	0.0000000000000000 0.0000000000000000 0.0000000000000000 F F F
0.0000000000000000 0.2000000000000028 0.0000000000000000 F F F	0.0000000000000000 0.2000000000000028 0.0000000000000000 F F F	0.0000000000000000 0.2000000000000028 0.0000000000000000 F F F
0.0000000000000000 0.3999999999999986 0.0000000000000000 F F F	0.0000000000000000 0.3999999999999986 0.0000000000000000 F F F	0.0000000000000000 0.3999999999999986 0.0000000000000000 F F F
0.0000000000000000 0.6000000000000014 0.0000000000000000 F F F	0.0000000000000000 0.6000000000000014 0.0000000000000000 F F F	0.0000000000000000 0.6000000000000014 0.0000000000000000 F F F
0.0000000000000000 0.7999999999999972 0.0000000000000000 F F F	0.0000000000000000 0.7999999999999972 0.0000000000000000 F F F	0.0000000000000000 0.7999999999999972 0.0000000000000000 F F F
0.2000000000000028 0.0000000000000000 0.0000000000000000 F F F	0.2000000000000028 0.0000000000000000 0.0000000000000000 F F F	0.2000000000000028 0.0000000000000000 0.0000000000000000 F F F
0.2000000000000028 0.2000000000000028 0.0000000000000000 F F F	0.2000000000000028 0.2000000000000028 0.0000000000000000 F F F	0.2000000000000028 0.2000000000000028 0.0000000000000000 F F F
0.2000000000000028 0.3999999999999986 0.0000000000000000 F F F	0.2000000000000028 0.3999999999999986 0.0000000000000000 F F F	0.2000000000000028 0.3999999999999986 0.0000000000000000 F F F
0.2000000000000028 0.6000000000000014 0.0000000000000000 F F F	0.2000000000000028 0.6000000000000014 0.0000000000000000 F F F	0.2000000000000028 0.6000000000000014 0.0000000000000000 F F F
0.2000000000000028 0.7999999999999972 0.0000000000000000 F F F	0.2000000000000028 0.7999999999999972 0.0000000000000000 F F F	0.2000000000000028 0.7999999999999972 0.0000000000000000 F F F
0.3999999999999986 0.0000000000000000 0.0000000000000000 F F F	0.3999999999999986 0.0000000000000000 0.0000000000000000 F F F	0.3999999999999986 0.0000000000000000 0.0000000000000000 F F F
0.3999999999999986 0.2000000000000028 0.0000000000000000 F F F	0.3999999999999986 0.2000000000000028 0.0000000000000000 F F F	0.3999999999999986 0.2000000000000028 0.0000000000000000 F F F
0.3999999999999986 0.3999999999999986 0.0000000000000000 F F F	0.3999999999999986 0.3999999999999986 0.0000000000000000 F F F	0.3999999999999986 0.3999999999999986 0.0000000000000000 F F F
0.3999999999999986 0.6000000000000014 0.0000000000000000 F F F	0.3999999999999986 0.6000000000000014 0.0000000000000000 F F F	0.3999999999999986 0.6000000000000014 0.0000000000000000 F F F
0.3999999999999986 0.7999999999999972 0.0000000000000000 F F F	0.3999999999999986 0.7999999999999972 0.0000000000000000 F F F	0.3999999999999986 0.7999999999999972 0.0000000000000000 F F F
0.6000000000000014 0.0000000000000000 0.0000000000000000 F F F	0.6000000000000014 0.0000000000000000 0.0000000000000000 F F F	0.6000000000000014 0.0000000000000000 0.0000000000000000 F F F
0.6000000000000014 0.2000000000000028 0.0000000000000000 F F F	0.6000000000000014 0.2000000000000028 0.0000000000000000 F F F	0.6000000000000014 0.2000000000000028 0.0000000000000000 F F F
0.6000000000000014 0.3999999999999986 0.0000000000000000 F F F	0.6000000000000014 0.3999999999999986 0.0000000000000000 F F F	0.6000000000000014 0.3999999999999986 0.0000000000000000 F F F
0.6000000000000014 0.6000000000000014 0.0000000000000000 F F F	0.6000000000000014 0.6000000000000014 0.0000000000000000 F F F	0.6000000000000014 0.6000000000000014 0.0000000000000000 F F F
0.6000000000000014 0.7999999999999972 0.0000000000000000 F F F	0.6000000000000014 0.7999999999999972 0.0000000000000000 F F F	0.6000000000000014 0.7999999999999972 0.0000000000000000 F F F
0.7999999999999972 0.0000000000000000 0.0000000000000000 F F F	0.7999999999999972 0.0000000000000000 0.0000000000000000 F F F	0.7999999999999972 0.0000000000000000 0.0000000000000000 F F F
0.7999999999999972 0.2000000000000028 0.0000000000000000 F F F	0.7999999999999972 0.2000000000000028 0.0000000000000000 F F F	0.7999999999999972 0.2000000000000028 0.0000000000000000 F F F
0.7999999999999972 0.3999999999999986 0.0000000000000000 F F F	0.7999999999999972 0.3999999999999986 0.0000000000000000 F F F	0.7999999999999972 0.3999999999999986 0.0000000000000000 F F F
0.7999999999999972 0.6000000000000014 0.0000000000000000 F F F	0.7999999999999972 0.6000000000000014 0.0000000000000000 F F F	0.7999999999999972 0.6000000000000014 0.0000000000000000 F F F
0.7999999999999972 0.7999999999999972 0.0000000000000000 F F F	0.7999999999999972 0.7999999999999972 0.0000000000000000 F F F	0.7999999999999972 0.7999999999999972 0.0000000000000000 F F F
0.2754853223973319 0.9280542119740220 0.1321050992058303 T T T	0.2779467328907058 0.9274786982060373 0.1328680434655334 T T T	0.2764735790403282 0.9298606641832522 0.1317411825212328 T T T
0.4666644456031577 0.7333340047990546 0.1368027475005462 T T T	0.4666644456031577 0.7333340047990546 0.1368027475005462 T T T	0.4666644456031577 0.7333340047990546 0.1368027475005462 T T T
0.6666644456031534 0.5333340047990589 0.1368027475005462 T T T	0.6666644456031534 0.5333340047990589 0.1368027475005462 T T T	0.6666644456031534 0.5333340047990589 0.1368027475005462 T T T
0.9148840435160694 0.3929005659329380 0.1418594404705856 T T T	0.8717571811346642 0.3463703330189130 0.1335636207064972 T T T	0.8777576134271079 0.3515309694160303 0.1346822972908201 T T T
0.2657420588702997 0.7332813529504331 0.1304389382652731 T T T	0.2674400484483616 0.7319499330193718 0.1308969524243295 T T T	0.2666048300313900 0.7335834981613010 0.1301761024445074 T T T
0.4741852410382195 0.9369321166432620 0.1336590460086046 T T T	0.4765484565139981 0.9383210172820501 0.1340200845263425 T T T	0.4745407560315668 0.9379265828528840 0.1337459772896743 T T T
0.4666644456031577 0.5333340047990589 0.1368027475005462 T T T	0.4666644456031577 0.5333340047990589 0.1368027475005462 T T T	0.4666644456031577 0.5333340047990589 0.1368027475005462 T T T
0.6666644456031534 0.7333340047990546 0.1368027475005462 T T T	0.6666644456031534 0.7333340047990546 0.1368027475005462 T T T	0.6666644456031534 0.7333340047990546 0.1368027475005462 T T T
0.8689739798321495 0.5503257444040384 0.1318670190409217 T T T	0.8653970596813474 0.5363846600766958 0.1314306819210543 T T T	0.8662518373325289 0.5375538636040946 0.1314145509677827 T T T
0.6810023127692383 0.3429524475393212 0.1329783987262073 T T T	0.6692023224909674 0.3363639594151375 0.1323481067978575 T T T	0.6708947713819536 0.3369829216146950 0.1325678499264052 T T T
0.2554994795859796 0.5247714509766100 0.1337815173310091 T T T	0.2589824985372806 0.5254936080949807 0.1326465464557882 T T T	0.2622532498248409 0.5278208214523722 0.1342726410511423 T T T
0.4706228454925862 0.3357591357267293 0.1337186116044826 T T T	0.4683233368218829 0.3347309092086306 0.1331334936417652 T T T	0.4685263025065909 0.3350024299494282 0.1333734119339760 T T T
0.2724162563437057 0.3345251642184403 0.1291092241241057 T T T	0.2732318904629051 0.3334434117114330 0.1294872382279098 T T T	0.2721500916272092 0.3331579644641910 0.1284282945220616 T T T
0.6705103970574626 0.9385017892242716 0.1325544778275857 T T T	0.6736833502130224 0.9399538157300926 0.1323269745263141 T T T	0.6706103343121271 0.9385633358758662 0.1331608093891978 T T T
0.8615657789221387 0.7386133438463136 0.1375829405649014 T T T	0.8601118087533485 0.7320625544692392 0.1374244291348447 T T T	0.8602451692886606 0.7322910333966455 0.1374410415587587 T T T
0.8660202490703673 0.9348352038262692 0.1291917255091476 T T T	0.8651815342059140 0.9312423689408165 0.1298181727480951 T T T	0.8635210769997296 0.9306271705389524 0.1302661439093837 T T T
0.3515268595702523 0.8559791901326022 0.2646143106998788 T T T	0.3541601517979648 0.8523768136743111 0.2654714835874044 T T T	0.3492892470068243 0.8597172266856841 0.2644716483208983 T T T
0.5333313179997745 0.6666662410030355 0.2736054950010853 T T T	0.5333313179997745 0.6666662410030355 0.2736054950010853 T T T	0.5333313179997745 0.6666662410030355 0.2736054950010853 T T T
0.3024080918916358 0.6474429079468154 0.2636411558646067 T T T	0.3150809189174854 0.6482837129860780 0.2648873942946844 T T T	0.3171141202310075 0.6578289987268154 0.2626393219444799 T T T
0.5422227549871768 0.4634972219223635 0.2680778031855087 T T T	0.5344137895330142 0.4600229371722881 0.2672555413411487 T T T	0.5351891432324477 0.4612477584400704 0.2672863958469836 T T T
0.3429008566790230 0.4408863331893378 0.2708616790423115 T T T	0.3406524024591572 0.4508014211146286 0.2698799199726926 T T T	0.3390573677271339 0.4490365333504341 0.2669853258955897 T T T
0.5494226515305862 0.8688780568293838 0.2679776089179000 T T T	0.5538151415358672 0.8708394580930059 0.2654187142534057 T T T	0.5455141686826148 0.8676977395463875 0.2670019164774842 T T T
0.7268393318207258 0.6658184685287586 0.2758578135854551 T T T	0.7227275328386916 0.6615163406612473 0.2769200922946284 T T T	0.7228968380046429 0.6624667609390483 0.2759567113862244 T T T
0.7410033700217820 0.8680209527206089 0.2611271031844126 T T T	0.7419418937828427 0.8640266021883283 0.2636974017904944 T T T	0.7371840144524868 0.8643462216027873 0.2642781730170591 T T T
0.7417995616102280 0.4788628266180843 0.2627524974422909 T T T	0.7330265553279214 0.4712193237563865 0.2612882265690246 T T T	0.7342432525651291 0.4724195666770727 0.2612703659164554 T T T
0.4199930313257727 0.7675017991715182 0.3912758324158004 T T T	0.4321037077929593 0.7624078311912311 0.3877804613338307 T T T	0.4203835746834200 0.7754145642038496 0.3924705580488284 T T T
0.3326257673607005 0.5511734291025511 0.3961446476130203 T T T	0.2968202780735863 0.5354555102654469 0.4015468424941161 T T T	0.3115998945871231 0.5483228439766182 0.3929997520121437 T T T
0.6305695713035999 0.7939000299613547 0.3974806687305341 T T T	0.6334291557055264 0.7743224847555686 0.4011030484882953 T T T	0.6197480358023882 0.7833418224340384 0.3997337600019402 T T T
0.5540743993567845 0.5730892511733611 0.4025654818151094 T T T	0.4957439547243520 0.5434102166369655 0.3979018267645351 T T T	0.5064427242322260 0.5491262981284272 0.3998077152763629 T T T
0.4819548441992225 0.6678088245021883 0.5079311926994450 T T T	0.4681485590556491 0.6633578456454501 0.5053766494427947 T T T	0.4577938451979691 0.6600196115028965 0.5034607420002999 T T T
0.1948979619679175 0.4354987649826234 0.3187145500790093 T T T	0.1983926890850226 0.4716993519208851 0.2641848229993236 T T T	0.2017419951583855 0.4796043147600400 0.2878442797819495 T T T
0.1520632172441079 0.4693876247846010 0.2423382806841823 T T T	0.0840457080685928 0.4081328119604031 0.2494396634305433 T T T	0.0900384175615799 0.4180793399952625 0.2637762456418165 T T T
0.0299071774453494 0.3327213129785165 0.3418969059994203 T T T	0.0811463045534051 0.3825806330331169 0.4341034536557290 T T T	0.9575504127459556 0.3267717907606929 0.4742459420470073 T T T
0.1216140089162640 0.6403281611538626 0.6049935409320039 T T T	0.0935443194829141 0.5897099729750483 0.6184792503901948 T T T	0.1084314166833238 0.6286543230042945 0.6025014530927951 T T T
0.0548303461594674 0.5658161727860495 0.5243887781969900 T T T	0.1225887822540675 0.4808243947291030 0.5089855367821633 T T T	0.0788650181234323 0.3995233385597401 0.4255570187039974 T T T
0.3468346232764675 0.6561279919594573 0.4748624442031349 T T T	0.1817363829243160 0.6654077795006089 0.5439047115413121 T T T	0.0933522887661183 0.5389955278993832 0.5288406015456194 T T T
0.1062979769565863 0.3514217476198621 0.3713175605133378 T T T	0.1460706267863556 0.4289095614307292 0.4752589448114898 T T T	0.0417849486807895 0.3857000176821640 0.4836203904516509 T T T
0.0360737784583452 0.4173627581253126 0.2318436661195032 T T T	0.9758340433608577 0.3491031599363528 0.2377360539017425 T T T	0.9849399689309276 0.3604719042692881 0.2422250092697757 T T T
0.0459516610100328 0.6085433142975686 0.5736921802055815 T T T	0.1066593371177582 0.5912176135088791 0.5556790224022721 T T T	0.1332636009250236 0.6252216887110227 0.5431219664371980 T T T

Table S7. Atomic coordinates of IS, TS, and FS of Reaction 3b listed in Table S1.

Reaction 3b: *COH–CO+ H^++e^-→*C–CO + H_2O on Cu-DAN via surface *H transfer		
IS	TS	FS
Cu C H O	Cu C H O	Cu C H O
1.00000000000000	1.00000000000000	1.0
12.8585004805000001 0.0000000000000000 0.0000000000000000	12.8585004805000001 0.0000000000000000 0.0000000000000000	12.8585004805000001 0.0000000000000000 0.0000000000000000
-6.4291002405000004 11.1357880710000003 0.0000000000000000	-6.4291002405000004 11.1357880710000003 0.0000000000000000	-6.4291002405000004 11.1357880710000003 0.0000000000000000
0.0000000000000000 0.0000000000000000 15.2994003295999992	0.0000000000000000 0.0000000000000000 15.2994003295999992	0.0000000000000000 0.0000000000000000 15.2994003295999992
Cu C H O	Cu C H O	Cu C H O
55 2 5 2	55 2 5 2	55 2 5 2
Selective	Selective dynamics	Selective
Direct	Direct	Direct
0.0000000000000000 0.0000000000000000 0.0000000000000000 F F F	0.0000000000000000 0.0000000000000000 0.0000000000000000 F F F	0.0000000000000000 0.0000000000000000 0.0000000000000000 F F F
0.0000000000000000 0.2000000000000028 0.0000000000000000 F F F	0.0000000000000000 0.2000000000000028 0.0000000000000000 F F F	0.0000000000000000 0.2000000000000030 0.0000000000000000 F F F
0.0000000000000000 0.3999999999999986 0.0000000000000000 F F F	0.0000000000000000 0.3999999999999986 0.0000000000000000 F F F	0.0000000000000000 0.3999999999999990 0.0000000000000000 F F F
0.0000000000000000 0.6000000000000014 0.0000000000000000 F F F	0.0000000000000000 0.6000000000000014 0.0000000000000000 F F F	0.0000000000000000 0.6000000000000010 0.0000000000000000 F F F
0.0000000000000000 0.7999999999999972 0.0000000000000000 F F F	0.0000000000000000 0.7999999999999972 0.0000000000000000 F F F	0.0000000000000000 0.7999999999999970 0.0000000000000000 F F F
0.2000000000000028 0.0000000000000000 0.0000000000000000 F F F	0.2000000000000028 0.0000000000000000 0.0000000000000000 F F F	0.2000000000000030 0.0000000000000000 0.0000000000000000 F F F
0.2000000000000028 0.2000000000000028 0.0000000000000000 F F F	0.2000000000000028 0.2000000000000028 0.0000000000000000 F F F	0.2000000000000030 0.2000000000000030 0.0000000000000000 F F F
0.2000000000000028 0.3999999999999986 0.0000000000000000 F F F	0.2000000000000028 0.3999999999999986 0.0000000000000000 F F F	0.2000000000000030 0.3999999999999990 0.0000000000000000 F F F
0.2000000000000028 0.6000000000000014 0.0000000000000000 F F F	0.2000000000000028 0.6000000000000014 0.0000000000000000 F F F	0.2000000000000030 0.6000000000000010 0.0000000000000000 F F F
0.2000000000000028 0.7999999999999972 0.0000000000000000 F F F	0.2000000000000028 0.7999999999999972 0.0000000000000000 F F F	0.2000000000000030 0.7999999999999970 0.0000000000000000 F F F
0.3999999999999986 0.0000000000000000 0.0000000000000000 F F F	0.3999999999999986 0.0000000000000000 0.0000000000000000 F F F	0.3999999999999990 0.0000000000000000 0.0000000000000000 F F F
0.3999999999999986 0.2000000000000028 0.0000000000000000 F F F	0.3999999999999986 0.2000000000000028 0.0000000000000000 F F F	0.3999999999999990 0.2000000000000030 0.0000000000000000 F F F
0.3999999999999986 0.3999999999999986 0.0000000000000000 F F F	0.3999999999999986 0.3999999999999986 0.0000000000000000 F F F	0.3999999999999990 0.3999999999999990 0.0000000000000000 F F F
0.3999999999999986 0.6000000000000014 0.0000000000000000 F F F	0.3999999999999986 0.6000000000000014 0.0000000000000000 F F F	0.3999999999999990 0.6000000000000010 0.0000000000000000 F F F
0.3999999999999986 0.7999999999999972 0.0000000000000000 F F F	0.3999999999999986 0.7999999999999972 0.0000000000000000 F F F	0.3999999999999990 0.7999999999999970 0.0000000000000000 F F F
0.6000000000000014 0.0000000000000000 0.0000000000000000 F F F	0.6000000000000014 0.0000000000000000 0.0000000000000000 F F F	0.6000000000000010 0.0000000000000000 0.0000000000000000 F F F
0.6000000000000014 0.2000000000000028 0.0000000000000000 F F F	0.6000000000000014 0.2000000000000028 0.0000000000000000 F F F	0.6000000000000010 0.2000000000000030 0.0000000000000000 F F F
0.6000000000000014 0.3999999999999986 0.0000000000000000 F F F	0.6000000000000014 0.3999999999999986 0.0000000000000000 F F F	0.6000000000000010 0.3999999999999990 0.0000000000000000 F F F
0.6000000000000014 0.6000000000000014 0.0000000000000000 F F F	0.6000000000000014 0.6000000000000014 0.0000000000000000 F F F	0.6000000000000010 0.6000000000000010 0.0000000000000000 F F F
0.6000000000000014 0.7999999999999972 0.0000000000000000 F F F	0.6000000000000014 0.7999999999999972 0.0000000000000000 F F F	0.6000000000000010 0.7999999999999970 0.0000000000000000 F F F
0.7999999999999972 0.0000000000000000 0.0000000000000000 F F F	0.7999999999999972 0.0000000000000000 0.0000000000000000 F F F	0.7999999999999970 0.0000000000000000 0.0000000000000000 F F F
0.7999999999999972 0.2000000000000028 0.0000000000000000 F F F	0.7999999999999972 0.2000000000000028 0.0000000000000000 F F F	0.7999999999999970 0.2000000000000030 0.0000000000000000 F F F
0.7999999999999972 0.3999999999999986 0.0000000000000000 F F F	0.7999999999999972 0.3999999999999986 0.0000000000000000 F F F	0.7999999999999970 0.3999999999999990 0.0000000000000000 F F F
0.7999999999999972 0.6000000000000014 0.0000000000000000 F F F	0.7999999999999972 0.6000000000000014 0.0000000000000000 F F F	0.7999999999999970 0.6000000000000010 0.0000000000000000 F F F
0.7999999999999972 0.7999999999999972 0.0000000000000000 F F F	0.7999999999999972 0.7999999999999972 0.0000000000000000 F F F	0.7999999999999970 0.7999999999999970 0.0000000000000000 F F F
0.2752053958105344 0.9339439589761694 0.1327670696624419 T T T	0.2774982481182827 0.9354884448322739 0.1329477155694769 T T T	0.2754785041014220 0.9346477123971870 0.1319474087029630 T T T
0.4666644456187115 0.7333340047990546 0.1368027474874722 T T T	0.4666644456098155 0.7333340047990546 0.1368027474949471 T T T	0.4666644456031580 0.7333340047990550 0.1368027475005460 T T T
0.6666644456187072 0.5333340047990589 0.1368027474874722 T T T	0.6666644456098112 0.5333340047990589 0.1368027474949471 T T T	0.6666644456031530 0.5333340047990590 0.1368027475005460 T T T
0.9102105890265042 0.3684869562188853 0.1436267400812801 T T T	0.9007223515121709 0.3658418985977799 0.1427679051892002 T T T	0.8795853102034310 0.3535464450022020 0.1344318105018020 T T T
0.2640084312338805 0.7324654224752489 0.1277175090534191 T T T	0.2662541670287100 0.7331485566375998 0.1274757468213157 T T T	0.2650038568977710 0.7304563758970271 0.1276427338019130 T T T
0.4727051250732295 0.9330780525467548 0.1334889884629417 T T T	0.4750414662286031 0.9346744188913859 0.1329483286072327 T T T	0.4735059615993860 0.9334180497982980 0.1325727993976220 T T T
0.4666644456187115 0.5333340047990589 0.1368027474874722 T T T	0.4666644456098155 0.5333340047990589 0.1368027474949471 T T T	0.4666644456031580 0.5333340047990590 0.1368027475005460 T T T
0.6666644456187072 0.7333340047990546 0.1368027474874722 T T T	0.6666644456098112 0.7333340047990546 0.1368027474949471 T T T	0.6666644456031530 0.7333340047990550 0.1368027475005460 T T T
0.8717735845097668 0.5382143799940207 0.1289171270272521 T T T	0.8701259946768864 0.5395360414207374 0.1297078836318406 T T T	0.8665319350962970 0.5392709172994120 0.1315233291991760 T T T
0.6785653253105682 0.3422523273193867 0.1341273253941728 T T T	0.6773308631856756 0.3405063651678481 0.1332151652333212 T T T	0.6717288015992650 0.3387514112022100 0.1333126033021020 T T T
0.2477907084217909 0.5256224929232950 0.1339067486259621 T T T	0.2354843020731359 0.5153230415825005 0.1372082025447597 T T T	0.2618586349985180 0.5261256970988550 0.1367820835991370 T T T
0.4616251920961347 0.3378668945654455 0.1375267820476050 T T T	0.4653581309603667 0.3365351750960127 0.1349948850031779 T T T	0.4683740083990880 0.3362468962974600 0.1337736493005080 T T T
0.2545863581217992 0.3376575447231827 0.1315045518840904 T T T	0.2646092271826690 0.3363928013807855 0.1278492189900385 T T T	0.2703869120018250 0.3307954519979650 0.1273849746012200 T T T
0.6697290446791372 0.9385423379270396 0.1324561431277958 T T T	0.6728247724267694 0.9394092289996248 0.1319635286257767 T T T	0.6705988258026350 0.9386255072975160 0.1335289338986390 T T T
0.8627709831984931 0.7314896965716159 0.1347630218409190 T T T	0.8609479911620095 0.7316887787340370 0.1361954327603670 T T T	0.8615832712012120 0.7324887824995659 0.1363858865019050 T T T
0.8636861106498250 0.9309436314968084 0.1295527729413490 T T T	0.8649482030392173 0.9315244790236371 0.1294688869249080 T T T	0.8637590566978660 0.9315684116973700 0.1302542951009970 T T T
0.3376756246337181 0.8572032306723175 0.2653428931031695 T T T	0.3422376229956424 0.8601405206216430 0.2644618701805572 T T T	0.3397644656954950 0.8641590507959260 0.2626543217007940 T T T
0.5333313180231087 0.6666662410030355 0.2736054949749445 T T T	0.5333313180097790 0.6666662410030355 0.2736054949898943 T T T	0.5333313179997750 0.6666662410030360 0.2736054950010850 T T T
0.3111338473132184 0.6526030185607582 0.2630663753300698 T T T	0.3080357857629651 0.6525935692435644 0.2642256395240517 T T T	0.3135840463020050 0.6629872780020490 0.2640669819969120 T T T
0.5581294652948900 0.4659464274831417 0.2718448985462245 T T T	0.5497468604026643 0.4621673103686444 0.2692921253482010 T T T	0.5390479424052400 0.4626556214029450 0.2688265743032260 T T T
0.3528770182459049 0.4067714450049785 0.2711019715667438 T T T	0.3487567296987099 0.4251667073487765 0.2681354252546602 T T T	0.3420350012947040 0.4435534365962890 0.2641533464015000 T T T
0.5429170065121565 0.8672547529853452 0.2685154323681632 T T T	0.5493111186923897 0.8711269806913090 0.2654399713897201 T T T	0.5427583814999440 0.8675087142020740 0.2689453753974430 T T T
0.7326360558265756 0.6715886588173202 0.2724439713953231 T T T	0.7264944204977748 0.6658376054046979 0.2759342377610673 T T T	0.7243744524994820 0.6635763254011380 0.2764048854005380 T T T
0.7364443251447784 0.8710149056858995 0.2614121622517396 T T T	0.7389722231085872 0.8677909422875612 0.2625008672057105 T T T	0.7356356616027140 0.8661251495992131 0.2632147045991640 T T T
0.7575375311771058 0.4899374496393202 0.2616536838231379 T T T	0.7493015837535477 0.4827523357739296 0.2611099287241918 T T T	0.7376270041990550 0.4766004875956110 0.2634005536023100 T T T
0.4108278119102587 0.7569425423433168 0.3949785249229972 T T T	0.4199069908416884 0.7678319176470986 0.3912856924123126 T T T	0.4114909273967410 0.7721433918980690 0.3973085689992500 T T T
0.3622578115283588 0.5438237276776903 0.3799756615266006 T T T	0.3463966307119719 0.5477506688880425 0.3847793362525636 T T T	0.3345007450996960 0.5506958747958000 0.3883820922970360 T T T
0.6248134710537756 0.8010599682900509 0.3967195754218141 T T T	0.6223475698054410 0.7970668628766425 0.3968795590981641 T T T	0.6186337585053470 0.7948775416040310 0.3983460441001010 T T T
0.5844222992818175 0.5879826871336482 0.4020482153950768 T T T	0.5607559580644494 0.5749599544572633 0.4007495255625768 T T T	0.5502113912001930 0.5727072819038740 0.4026802159023650 T T T
0.4816708519180253 0.6590139467013927 0.5009420032674186 T T T	0.4732109073934367 0.6606487788709074 0.5024867163153246 T T T	0.4668659490000100 0.6618749029980510 0.5036452511012540 T T T
0.2056445540343730 0.4071932828588076 0.3075310875910325 T T T	0.2010129379630213 0.4192742070413628 0.3087012158925224 T T T	0.2110866705969200 0.4809927662999270 0.2787468453027630 T T T
0.1486522525363599 0.4060366934049549 0.2248036335478256 T T T	0.1311237832861465 0.4132812835590142 0.2354065190013193 T T T	0.0972033558029182 0.4224741589014020 0.2591156015004190 T T T
0.0468107870493780 0.3306116326002592 0.3525109881295637 T T T	0.0552793351659478 0.3587456466031958 0.3899922197670536 T T T	0.0050579217003133 0.2668402119952660 0.4009300565939500 T T T
0.2779164418935965 0.9403804602137472 0.2395338777622968 T T T	0.2878295783873985 0.9481175115174425 0.2395225580296755 T T T	0.2890064198992730 0.9557903928971070 0.2379681478989820 T T T
0.1963965594648570 0.6144200045825042 0.1913983494226776 T T T	0.1955829467265681 0.6168592419222305 0.1910893205382998 T T T	0.1963965595029290 0.6144200045992390 0.1913983494003090 T T T
0.2893761197383481 0.7440543424158584 0.3406825034212908 T T T	0.2935840567295274 0.7489015871697543 0.3427520778048979 T T T	0.2912995514023460 0.7573862270030250 0.3392410654983920 T T T
0.3456372728707347 0.6316951974031232 0.4678288473353099 T T T	0.2255896914902443 0.4505774013271088 0.4412649723606199 T T T	0.1487918408246570 0.3700218268728220 0.4506394668202200 T T T
0.1275809400186538 0.3534499251203177 0.3755849822832679 T T T	0.1333341442832632 0.3607737919126963 0.3914767424607822 T T T	0.0858280746966855 0.2896785044967490 0.4240040507959930 T T T
0.0322154251444465 0.3554680308979235 0.2221100895220382 T T T	0.0160986675899098 0.3648527187402158 0.2340971901011210 T T T	0.9906429924986140 0.3683237032034940 0.2416208720970590 T T T

Table S8. Atomic coordinates of IS, TS, and FS of Reaction 4a listed in Table S1.

Reaction 4a: *COH–CO+ H^++e^-→*COH–CHO on Cu-DAN via surface *H transfer

IS	TS	FS
Cu C H O	Cu C H O	Cu C H O
1.00000000000000	1.00000000000000	1.00000000000000
12.8585004805000001 0.0000000000000000 0.0000000000000000	12.8585004805000001 0.0000000000000000 0.0000000000000000	12.8585004805000001 0.0000000000000000 0.0000000000000000
-6.4291002405000004 11.1357880710000003 0.0000000000000000	-6.4291002405000004 11.1357880710000003 0.0000000000000000	-6.4291002405000004 11.1357880710000003 0.0000000000000000
0.0000000000000000 0.0000000000000000 15.2994003295999992	0.0000000000000000 0.0000000000000000 15.2994003295999992	0.0000000000000000 0.0000000000000000 15.2994003295999992
Cu C H O	Cu C H O	Cu C H O
55 2 4 3	55 2 4 3	55 2 4 3
Selective dynamics	Selective dynamics	Selective dynamics
Direct	Direct	Direct
0.0000000000000000 0.0000000000000000 0.0000000000000000 F F F	0.0000000000000000 0.0000000000000000 0.0000000000000000 F F F	0.0000000000000000 0.0000000000000000 0.0000000000000000 F F F
0.0000000000000000 0.2000000000000028 0.0000000000000000 F F F	0.0000000000000000 0.2000000000000028 0.0000000000000000 F F F	0.0000000000000000 0.2000000000000028 0.0000000000000000 F F F
0.0000000000000000 0.3999999999999986 0.0000000000000000 F F F	0.0000000000000000 0.3999999999999986 0.0000000000000000 F F F	0.0000000000000000 0.3999999999999986 0.0000000000000000 F F F
0.0000000000000000 0.6000000000000014 0.0000000000000000 F F F	0.0000000000000000 0.6000000000000014 0.0000000000000000 F F F	0.0000000000000000 0.6000000000000014 0.0000000000000000 F F F
0.0000000000000000 0.7999999999999972 0.0000000000000000 F F F	0.0000000000000000 0.7999999999999972 0.0000000000000000 F F F	0.0000000000000000 0.7999999999999972 0.0000000000000000 F F F
0.2000000000000028 0.0000000000000000 0.0000000000000000 F F F	0.2000000000000028 0.0000000000000000 0.0000000000000000 F F F	0.2000000000000028 0.0000000000000000 0.0000000000000000 F F F
0.2000000000000028 0.2000000000000028 0.0000000000000000 F F F	0.2000000000000028 0.2000000000000028 0.0000000000000000 F F F	0.2000000000000028 0.2000000000000028 0.0000000000000000 F F F
0.2000000000000028 0.3999999999999986 0.0000000000000000 F F F	0.2000000000000028 0.3999999999999986 0.0000000000000000 F F F	0.2000000000000028 0.3999999999999986 0.0000000000000000 F F F
0.2000000000000028 0.6000000000000014 0.0000000000000000 F F F	0.2000000000000028 0.6000000000000014 0.0000000000000000 F F F	0.2000000000000028 0.6000000000000014 0.0000000000000000 F F F
0.2000000000000028 0.7999999999999972 0.0000000000000000 F F F	0.2000000000000028 0.7999999999999972 0.0000000000000000 F F F	0.2000000000000028 0.7999999999999972 0.0000000000000000 F F F
0.3999999999999986 0.0000000000000000 0.0000000000000000 F F F	0.3999999999999986 0.0000000000000000 0.0000000000000000 F F F	0.3999999999999986 0.0000000000000000 0.0000000000000000 F F F
0.3999999999999986 0.2000000000000028 0.0000000000000000 F F F	0.3999999999999986 0.2000000000000028 0.0000000000000000 F F F	0.3999999999999986 0.2000000000000028 0.0000000000000000 F F F
0.3999999999999986 0.3999999999999986 0.0000000000000000 F F F	0.3999999999999986 0.3999999999999986 0.0000000000000000 F F F	0.3999999999999986 0.3999999999999986 0.0000000000000000 F F F
0.3999999999999986 0.6000000000000014 0.0000000000000000 F F F	0.3999999999999986 0.6000000000000014 0.0000000000000000 F F F	0.3999999999999986 0.6000000000000014 0.0000000000000000 F F F
0.3999999999999986 0.7999999999999972 0.0000000000000000 F F F	0.3999999999999986 0.7999999999999972 0.0000000000000000 F F F	0.3999999999999986 0.7999999999999972 0.0000000000000000 F F F
0.6000000000000014 0.0000000000000000 0.0000000000000000 F F F	0.6000000000000014 0.0000000000000000 0.0000000000000000 F F F	0.6000000000000014 0.0000000000000000 0.0000000000000000 F F F
0.6000000000000014 0.2000000000000028 0.0000000000000000 F F F	0.6000000000000014 0.2000000000000028 0.0000000000000000 F F F	0.6000000000000014 0.2000000000000028 0.0000000000000000 F F F
0.6000000000000014 0.3999999999999986 0.0000000000000000 F F F	0.6000000000000014 0.3999999999999986 0.0000000000000000 F F F	0.6000000000000014 0.3999999999999986 0.0000000000000000 F F F
0.6000000000000014 0.6000000000000014 0.0000000000000000 F F F	0.6000000000000014 0.6000000000000014 0.0000000000000000 F F F	0.6000000000000014 0.6000000000000014 0.0000000000000000 F F F
0.6000000000000014 0.7999999999999972 0.0000000000000000 F F F	0.6000000000000014 0.7999999999999972 0.0000000000000000 F F F	0.6000000000000014 0.7999999999999972 0.0000000000000000 F F F
0.7999999999999972 0.0000000000000000 0.0000000000000000 F F F	0.7999999999999972 0.0000000000000000 0.0000000000000000 F F F	0.7999999999999972 0.0000000000000000 0.0000000000000000 F F F
0.7999999999999972 0.2000000000000028 0.0000000000000000 F F F	0.7999999999999972 0.2000000000000028 0.0000000000000000 F F F	0.7999999999999972 0.2000000000000028 0.0000000000000000 F F F
0.7999999999999972 0.3999999999999986 0.0000000000000000 F F F	0.7999999999999972 0.3999999999999986 0.0000000000000000 F F F	0.7999999999999972 0.3999999999999986 0.0000000000000000 F F F
0.7999999999999972 0.6000000000000014 0.0000000000000000 F F F	0.7999999999999972 0.6000000000000014 0.0000000000000000 F F F	0.7999999999999972 0.6000000000000014 0.0000000000000000 F F F
0.7999999999999972 0.7999999999999972 0.0000000000000000 F F F	0.7999999999999972 0.7999999999999972 0.0000000000000000 F F F	0.7999999999999972 0.7999999999999972 0.0000000000000000 F F F
0.2796943016308203 0.9317068161380664 0.1305827619309295 T T T	0.2728232980128804 0.9289203393505542 0.1306950360003955 T T T	0.2729929277172859 0.9297772274426410 0.1303624970686971 T T T
0.4666644456187115 0.7333340047990546 0.1368027474874722 T T T	0.4666644456187115 0.7333340047990546 0.1368027474874722 T T T	0.4666644456187115 0.7333340047990546 0.1368027474874722 T T T
0.6666644456187072 0.5333340047990589 0.1368027474874722 T T T	0.6666644456187072 0.5333340047990589 0.1368027474874722 T T T	0.6666644456187072 0.5333340047990589 0.1368027474874722 T T T
0.9175099794238600 0.3880853146057087 0.1458854691989256 T T T	0.8944044523014442 0.3667593351122457 0.1395120083341442 T T T	0.8763302222758490 0.3480499878659764 0.1367199227794972 T T T
0.2533085952427774 0.7253665194305916 0.1314481873374182 T T T	0.2606745712737109 0.7307482473791244 0.1328530754242690 T T T	0.2632528336438612 0.7320122100427358 0.1310551650781501 T T T
0.4783399481237271 0.9416495440184156 0.1337600702483050 T T T	0.4716970210869418 0.9358193342744222 0.1331945540447110 T T T	0.4724672958381290 0.9375115904095712 0.1339604219939373 T T T
0.4666644456187115 0.5333340047990589 0.1368027474874722 T T T	0.4666644456187115 0.5333340047990589 0.1368027474874722 T T T	0.4666644456187115 0.5333340047990589 0.1368027474874722 T T T
0.6666644456187072 0.7333340047990546 0.1368027474874722 T T T	0.6666644456187072 0.7333340047990546 0.1368027474874722 T T T	0.6666644456187072 0.7333340047990546 0.1368027474874722 T T T
0.8699855904405507 0.5453100802765608 0.1308272382140089 T T T	0.8695077886097431 0.5424524584191067 0.1304016820028025 T T T	0.8632300421388935 0.5352322236001567 0.1330645504419588 T T T
0.6818592220855718 0.3421351895945606 0.1332703604629227 T T T	0.6772975941506491 0.3402118101477879 0.1330212619263656 T T T	0.6695979998212085 0.3344441805028023 0.1311932916691460 T T T
0.2561416618033415 0.5192572985736794 0.1321778646330710 T T T	0.2512805645492763 0.5213260569319559 0.1366960736788470 T T T	0.2630024879128680 0.5319940128653448 0.1320851954940759 T T T
0.4686727974263157 0.3372659541589615 0.1358728079260946 T T T	0.4674056166090474 0.3356631973370561 0.1343919758865350 T T T	0.4693380537603241 0.3361998894363065 0.1340762146762512 T T T
0.2670523026740779 0.3267528222889919 0.1240424656338551 T T T	0.2685596828047868 0.3330008430999249 0.1263944918334683 T T T	0.2719868866967235 0.3372022547287425 0.1295261834299136 T T T
0.6716969466481695 0.9379332295917626 0.1323399691318839 T T T	0.6678193031184636 0.9372940399213858 0.1338942002980632 T T T	0.6662839443765669 0.9351314581265198 0.1348008844516944 T T T
0.8619109667243207 0.7353853970291550 0.1360476120099854 T T T	0.8607393887905028 0.7339120813908122 0.1369627368592458 T T T	0.8617447072442188 0.7323946671200590 0.1355517970872361 T T T
0.8650197910289508 0.9327456683779652 0.1290444473648214 T T T	0.8618669230981428 0.9311895415565369 0.1305459826073564 T T T	0.8603403726474588 0.9289641138007266 0.1326634739992980 T T T
0.3507161948570421 0.8462644079096314 0.2584714235710577 T T T	0.3461391458659135 0.8658276638154017 0.2631055776282937 T T T	0.3413787839789078 0.8625602381081479 0.2615905859582662 T T T
0.5333313180231087 0.6666662410030355 0.2736054949749445 T T T	0.5333313180231087 0.6666662410030355 0.2736054949749445 T T T	0.5333313180231087 0.6666662410030355 0.2736054949749445 T T T
0.2908024755522102 0.6341065172237058 0.2643686007126078 T T T	0.3317095097561940 0.6704470806974940 0.2624157016483607 T T T	0.3326355253160293 0.6687060112976141 0.2613052940847528 T T T
0.5458464122683822 0.4650277933084669 0.2696768657316754 T T T	0.5441465780692447 0.4620701176442590 0.2681219684497911 T T T	0.5273354950788235 0.4652634918392893 0.2693340670326633 T T T
0.3373781858032339 0.4118079536463745 0.2626068858683608 T T T	0.3419374848654747 0.4299754471741756 0.2652035972477771 T T T	0.3265617026237381 0.4617753832798963 0.2654949335693352 T T T
0.5502983817159030 0.8714685655960007 0.2677671763920775 T T T	0.5393587131770825 0.8662659667988604 0.2700202428739741 T T T	0.5352061688015421 0.8668710122799136 0.2720857173377234 T T T
0.7290881567137978 0.6671477434291664 0.2744070680336458 T T T	0.7248271423278452 0.6647139113646018 0.2760743178111921 T T T	0.7246466891405701 0.6614912851850313 0.2714003959526963 T T T
0.7411578783569842 0.8680250205903626 0.2623892239779709 T T T	0.7339154106428359 0.8652626521129144 0.2645594668667074 T T T	0.7265021148722226 0.8601810089293906 0.2685437365384400 T T T
0.7455109565654552 0.4812121052110473 0.2617164809868030 T T T	0.7452272738740533 0.4800562274070552 0.2610593869639999 T T T	0.7219078882703610 0.4632966554492442 0.2614698036955323 T T T
0.4125213788326579 0.7598964598455814 0.3909939830361572 T T T	0.4051452680660595 0.7729831462623321 0.4006149400711324 T T T	0.3887391598520006 0.7776479447184177 0.3979788481618768 T T T
0.3622578115319257 0.5438237276787249 0.3799756615237435 T T T	0.3348440041259860 0.5472402711340377 0.3819020057979962 T T T	0.2771877113899249 0.5369733390800261 0.3929443525832658 T T T
0.6216535681157477 0.7985975633158312 0.3948803250730785 T T T	0.6107176323322914 0.7930376726618975 0.3974673025187130 T T T	0.5871625032142173 0.7825152397749671 0.4019081421640222 T T T
0.5762445455244836 0.5833255631365809 0.4014170577646454 T T T	0.5488976836399643 0.5702401343250676 0.4007720647824234 T T T	0.4778224321698687 0.5447083317272042 0.3997196185675860 T T T
0.4816708519165286 0.6590139467039255 0.5009420032697598 T T T	0.4640681246685778 0.6546150553182071 0.5008895418501699 T T T	0.4258947263493524 0.6522795539252482 0.5027346650835467 T T T
0.2047180917904713 0.4306545357868545 0.3191541795883955 T T T	0.1902802064495621 0.4198058753987398 0.3134140527561339 T T T	0.1550538427350676 0.4070576127933664 0.3155892641847701 T T T
0.1557958774749468 0.4516815445865356 0.2406408540963999 T T T	0.1323867339777658 0.4338454108453548 0.2359658458285511 T T T	0.0796452971650534 0.4273284003171841 0.2573422711969393 T T T
0.0420343146968721 0.3377012990390427 0.3544548036668936 T T T	0.0306582970082497 0.2974800241276276 0.3440386537931097 T T T	0.0220766247906616 0.2471247877565359 0.3194676099531389 T T T
0.2041709945575623 0.6173878941678945 0.4702911504705081 T T T	0.2146796787154242 0.6841859461963091 0.5042376236146758 T T T	0.2036528195659758 0.6878443661784567 0.4981184559168570 T T T
0.1872849022577914 0.6964500126003027 0.4019932208269788 T T T	0.2020909788827947 0.7671856853669379 0.4364053415692032 T T T	0.1857147513203133 0.7744052756524745 0.4375231712145397 T T T
0.2180635253554659 0.7176041731062375 0.2465285302907642 T T T	0.2021167872110124 0.5919335356926260 0.2038442145595609 T T T	0.1116551529887108 0.5206065411396487 0.2354004671430756 T T T
0.1208635771727074 0.3628179368423970 0.3821124431768357 T T T	0.1122618027033370 0.3290827368801025 0.3682538051379341 T T T	0.1019999065025345 0.2914223480294780 0.3475671965606031 T T T
0.0393589120680367 0.3982318888203152 0.2320645108510543 T T T	0.0186106060586901 0.3780375329417323 0.2273503701688108 T T T	0.9739232450350146 0.3420127999871758 0.2349742154017027 T T T
0.2310222580275883 0.7013226336985462 0.4558456108992784 T T T	0.2574560251108374 0.7649003548664119 0.4791788882818100 T T T	0.2414465449383610 0.7720313578417725 0.4798846608306375 T T T

Table S9. Atomic coordinates of IS, TS, and FS of Reaction 4c listed in Table S1.

Reaction 4b: *COH–CO+ H^++e^-→*COH–CHO on planar Cu (100) via surface *H transfer		
IS	TS	FS
Cu C H O<br>1.00000000000000<br>10.9033256753999996 0.0000000000000000 0.0000000000000000<br>0.0000000000000000 10.9033256753999996 0.0000000000000000<br>0.0000000000000000 0.0000000000000000 10.6344418917999999<br>Cu C H O<br>36 2 2 2<br>Selective dynamics<br>Direct<br>0.0000000000000000 0.0000000000000000 0.0000000000000000 F F F<br>0.1661041259954941 0.0008695378088244 0.1619882777796406 T T T<br>0.9995854207921430 0.1670516348230785 0.1649245184631450 T T T<br>0.1666666666666643 0.1666666666666643 0.0000000000000000 F F F<br>0.0000000000000000 0.3333333333333357 0.0000000000000000 F F F<br>0.1664415101985676 0.3332290967530651 0.1617822724428833 T T T<br>0.9997562743553990 0.4997578192274045 0.1622764859777086 T T T<br>0.1666666666666643 0.5000000000000000 0.0000000000000000 F F F<br>0.0000000000000000 0.6666666666666643 0.0000000000000000 F F F<br>0.1639901206847034 0.6682089858481457 0.1611648800777758 T T T<br>0.9991196295680950 0.8338781576929283 0.1620198075772152 T T T<br>0.1666666666666643 0.8333333333333357 0.0000000000000000 F F F<br>0.3333333333333357 0.0000000000000000 0.0000000000000000 F F F<br>0.5002715303656241 0.0002944146804081 0.1623122392668561 T T T<br>0.3336233147562353 0.1663903303844037 0.1621524171165534 T T T<br>0.5000000000000000 0.1666666666666643 0.0000000000000000 F F F<br>0.3333333333333357 0.3333333333333357 0.0000000000000000 F F F<br>0.5025121458238192 0.3314724932912396 0.1678101234102215 T T T<br>0.3318164580322804 0.5016036180781258 0.1692018541826215 T T T<br>0.5000000000000000 0.5000000000000000 0.0000000000000000 F F F<br>0.3333333333333357 0.6666666666666643 0.0000000000000000 F F F<br>0.4981462177966607 0.6681366770134289 0.1692124547645107 T T T<br>0.3317795142791436 0.8361575700248703 0.1611647837928961 T T T<br>0.5000000000000000 0.8333333333333357 0.0000000000000000 F F F<br>0.6666666666666643 0.0000000000000000 0.0000000000000000 F F F<br>0.8329676755448812 0.0002971881994578 0.1649166396738098 T T T<br>0.6682781463880616 0.1644864174007575 0.1651160889588236 T T T<br>0.8333333333333357 0.1666666666666643 0.0000000000000000 F F F<br>0.6666666666666643 0.3333333333333357 0.0000000000000000 F F F<br>0.8354067672288140 0.3318334795686460 0.1649933419499269 T T T<br>0.6684751656146590 0.4976723635318578 0.1677835236298524 T T T<br>0.8333333333333357 0.5000000000000000 0.0000000000000000 F F F<br>0.6666666666666643 0.6666666666666643 0.0000000000000000 F F F<br>0.8336068064568966 0.6664258514133563 0.1621331480542856 T T T<br>0.6666895664454081 0.8334454813277262 0.1617969452907017 T T T<br>0.8333333333333357 0.8333333333333357 0.0000000000000000 F F F<br>0.4554538836332186 0.5444410637074751 0.3044095668841552 T T T<br>0.5514402691501205 0.4484101218772729 0.3139921071042192 T T T<br>0.4611097447278152 0.5393161924450083 0.4832854698334224 T T T<br>0.8309714754755351 0.1695980991580818 0.2185727790337578 T T T<br>0.4146957401267218 0.5849918964564399 0.4185270683446837 T T T<br>0.5901722201266092 0.4102828789059565 0.4167617771546612 T T T	Cu C H O<br>1.00000000000000<br>10.9033256753999996 0.0000000000000000 0.0000000000000000<br>0.0000000000000000 10.9033256753999996 0.0000000000000000<br>0.0000000000000000 0.0000000000000000 10.6344418917999999<br>Cu C H O<br>36 2 2 2<br>Selective dynamics<br>Direct<br>0.0000000000000000 0.0000000000000000 0.0000000000000000 F F F<br>0.1660864284965375 0.0006090524000273 0.1627564135632032 T T T<br>0.9996828870111660 0.1668916261782245 0.1626618321231320 T T T<br>0.1666666666876395 0.1666666666876395 0.0000000000000000 F F F<br>0.0000000000000000 0.3333333333123605 0.0000000000000000 F F F<br>0.1658222599191973 0.3326776229563612 0.1619516827540445 T T T<br>0.9997830061794007 0.4998960179630280 0.1625296523692658 T T T<br>0.1666666666876395 0.5000000000000000 0.0000000000000000 F F F<br>0.0000000000000000 0.6666666666876395 0.0000000000000000 F F F<br>0.1633564751298621 0.6681582706254641 0.1617415544105775 T T T<br>0.9993978075783454 0.8339086054070536 0.1627536257032900 T T T<br>0.1666666666876395 0.8333333333123605 0.0000000000000000 F F F<br>0.3333333333123605 0.0000000000000000 0.0000000000000000 F F F<br>0.5001249938557017 0.0002197588932932 0.1625404939251031 T T T<br>0.3328861589628708 0.1657047200658749 0.1619740438635099 T T T<br>0.5000000000000000 0.1666666666876395 0.0000000000000000 F F F<br>0.3333333333123605 0.3333333333123605 0.0000000000000000 F F F<br>0.5006357921907516 0.3309144818895444 0.1663883799392043 T T T<br>0.3334125651700385 0.4995335749729921 0.1678294464889087 T T T<br>0.5000000000000000 0.5000000000000000 0.0000000000000000 F F F<br>0.3333333333123605 0.6666666666876395 0.0000000000000000 F F F<br>0.5001992639966042 0.6667820022390294 0.1680663349987641 T T T<br>0.3318355744755442 0.8366914310170881 0.1617232703452010 T T T<br>0.5000000000000000 0.8333333333123605 0.0000000000000000 F F F<br>0.6666666666876395 0.0000000000000000 0.0000000000000000 F F F<br>0.8330999758248234 0.0003237298301323 0.1626557912014171 T T T<br>0.6674386438918012 0.1649206114047846 0.1634686225089745 T T T<br>0.8333333333123605 0.1666666666876395 0.0000000000000000 F F F<br>0.6666666666876395 0.3333333333123605 0.0000000000000000 F F F<br>0.8351419044383710 0.3325601912296752 0.1634518135231096 T T T<br>0.6689797095209409 0.4993305772400200 0.1665047791496560 T T T<br>0.8333333333123605 0.5000000000000000 0.0000000000000000 F F F<br>0.6666666666876395 0.6666666666876395 0.0000000000000000 F F F<br>0.8342751768706803 0.6671091429721240 0.1619770986975105 T T T<br>0.6673077023610385 0.8341505597131198 0.1619687885209637 T T T<br>0.8333333333123605 0.8333333333123605 0.0000000000000000 F F F<br>0.4315929954476401 0.5676622453318021 0.3126968767666341 T T T<br>0.5127388659590715 0.4918038624039694 0.3746750242326554 T T T<br>0.3773172475163558 0.6196597526995888 0.4842695374618884 T T T<br>0.6272254083304288 0.3726698310489057 0.2440934425380004 T T T<br>0.3583034425455959 0.6391831356396294 0.3964899238392943 T T T<br>0.5604084871526547 0.4478124346847349 0.4646995466481276 T T T	Cu C H O<br>1.00000000000000<br>10.9033256753999996 0.0000000000000000 0.0000000000000000<br>0.0000000000000000 10.9033256753999996 0.0000000000000000<br>0.0000000000000000 0.0000000000000000 10.6344418917999999<br>Cu C H O<br>36 2 2 2<br>Selective dynamics<br>Direct<br>0.0000000000000000 0.0000000000000000 0.0000000000000000 F F F<br>0.1656183641702520 0.0009120206962821 0.1625777155000722 T T T<br>0.9996863978847994 0.1668664325953029 0.1627126800127553 T T T<br>0.1666666667033496 0.1666666667033496 0.0000000000000000 F F F<br>0.0000000000000000 0.3333333332966504 0.0000000000000000 F F F<br>0.1665743031880270 0.3330611318288907 0.1619518881803572 T T T<br>0.9991427312927184 0.4993331392748290 0.1625478197274055 T T T<br>0.1666666667033496 0.5000000000000000 0.0000000000000000 F F F<br>0.0000000000000000 0.6666666667033496 0.0000000000000000 F F F<br>0.1622367575357500 0.6677071395809152 0.1622162458447615 T T T<br>0.9991126624570652 0.8342478379006895 0.1625551841130585 T T T<br>0.1666666667033496 0.8333333332966504 0.0000000000000000 F F F<br>0.3333333332966504 0.0000000000000000 0.0000000000000000 F F F<br>0.5006544469543923 0.0008257786984203 0.1626075557627554 T T T<br>0.3329985705370204 0.1657798649206059 0.1628190670802992 T T T<br>0.5000000000000000 0.1666666667033496 0.0000000000000000 F F F<br>0.3333333332966504 0.3333333332966504 0.0000000000000000 F F F<br>0.5001192642486006 0.3299752870446936 0.1618319291567784 T T T<br>0.3334353045168431 0.4990121075085339 0.1676474627907450 T T T<br>0.5000000000000000 0.5000000000000000 0.0000000000000000 F F F<br>0.3333333332966504 0.6666666667033496 0.0000000000000000 F F F<br>0.5000054676479334 0.6671354351717098 0.1680288414207116 T T T<br>0.3322061947958173 0.8380210143808798 0.1620445200039283 T T T<br>0.5000000000000000 0.8333333332966504 0.0000000000000000 F F F<br>0.6666666667033496 0.0000000000000000 0.0000000000000000 F F F<br>0.8331263542994207 0.0001650938241613 0.1627000399878095 T T T<br>0.6670816427661515 0.1661724664039810 0.1625402005455117 T T T<br>0.8333333332966504 0.1666666667033496 0.0000000000000000 F F F<br>0.6666666667033496 0.3333333332966504 0.0000000000000000 F F F<br>0.8337281909035029 0.3329582240493775 0.1625430453029703 T T T<br>0.6698268903690056 0.4999588078432516 0.1617648901991565 T T T<br>0.8333333332966504 0.5000000000000000 0.0000000000000000 F F F<br>0.6666666667033496 0.6666666667033496 0.0000000000000000 F F F<br>0.8341611197343570 0.6669487047425560 0.1627844198494415 T T T<br>0.6667821242038060 0.8332780704974725 0.1619951397918860 T T T<br>0.8333333332966504 0.8333333332966504 0.0000000000000000 F F F<br>0.4077626903381310 0.5908809374249930 0.3109118082981768 T T T<br>0.4841703392037808 0.5201356331287620 0.3972210108965136 T T T<br>0.3444777582969926 0.6509239316088790 0.4660718536578153 T T T<br>0.5514938920491012 0.4553852715759961 0.3518780932489524 T T T<br>0.3274867100251982 0.6671011635961941 0.3754455049486158 T T T<br>0.4749276522117104 0.5311597608130554 0.5130415835288753 T T T

Table S10. Atomic coordinates of IS, TS, and FS of Reaction 6 listed in Table S1.

Reaction 5: *COH–CHO+ H^{+}+e^{-}→*CHOH–CHO on Cu-DAN via surface *H transfer

IS	TS	FS
Cu C H O	Cu C H O	Cu C H O
1.00000000000000	1.00000000000000	1.00000000000000
12.8585004805000001 0.0000000000000000 0.0000000000000000	12.8585004805000001 0.0000000000000000 0.0000000000000000	12.8585004805000001 0.0000000000000000 0.0000000000000000
-6.4291002405000004 11.1357880710000003 0.0000000000000000	-6.4291002405000004 11.1357880710000003 0.0000000000000000	-6.4291002405000004 11.1357880710000003 0.0000000000000000
0.0000000000000000 0.0000000000000000 15.2994003295999992	0.0000000000000000 0.0000000000000000 15.2994003295999992	0.0000000000000000 0.0000000000000000 15.2994003295999992
Cu C H O	Cu C H O	Cu C H O
55 2 3 2	55 2 3 2	55 2 3 2
Selective dynamics	Selective dynamics	Selective dynamics
Direct	Direct	Direct
0.0000000000000000 0.0000000000000000 0.0000000000000000 F F F	0.0000000000000000 0.0000000000000000 0.0000000000000000 F F F	0.0000000000000000 0.0000000000000000 0.0000000000000000 F F F
0.0000000000000000 0.2000000000000028 0.0000000000000000 F F F	0.0000000000000000 0.2000000000000028 0.0000000000000000 F F F	0.0000000000000000 0.2000000000000028 0.0000000000000000 F F F
0.0000000000000000 0.3999999999999986 0.0000000000000000 F F F	0.0000000000000000 0.3999999999999986 0.0000000000000000 F F F	0.0000000000000000 0.3999999999999986 0.0000000000000000 F F F
0.0000000000000000 0.6000000000000014 0.0000000000000000 F F F	0.0000000000000000 0.6000000000000014 0.0000000000000000 F F F	0.0000000000000000 0.6000000000000014 0.0000000000000000 F F F
0.0000000000000000 0.7999999999999972 0.0000000000000000 F F F	0.0000000000000000 0.7999999999999972 0.0000000000000000 F F F	0.0000000000000000 0.7999999999999972 0.0000000000000000 F F F
0.2000000000000028 0.0000000000000000 0.0000000000000000 F F F	0.2000000000000028 0.0000000000000000 0.0000000000000000 F F F	0.2000000000000028 0.0000000000000000 0.0000000000000000 F F F
0.2000000000000028 0.2000000000000028 0.0000000000000000 F F F	0.2000000000000028 0.2000000000000028 0.0000000000000000 F F F	0.2000000000000028 0.2000000000000028 0.0000000000000000 F F F
0.2000000000000028 0.3999999999999986 0.0000000000000000 F F F	0.2000000000000028 0.3999999999999986 0.0000000000000000 F F F	0.2000000000000028 0.3999999999999986 0.0000000000000000 F F F
0.2000000000000028 0.6000000000000014 0.0000000000000000 F F F	0.2000000000000028 0.6000000000000014 0.0000000000000000 F F F	0.2000000000000028 0.6000000000000014 0.0000000000000000 F F F
0.2000000000000028 0.7999999999999972 0.0000000000000000 F F F	0.2000000000000028 0.7999999999999972 0.0000000000000000 F F F	0.2000000000000028 0.7999999999999972 0.0000000000000000 F F F
0.3999999999999986 0.0000000000000000 0.0000000000000000 F F F	0.3999999999999986 0.0000000000000000 0.0000000000000000 F F F	0.3999999999999986 0.0000000000000000 0.0000000000000000 F F F
0.3999999999999986 0.2000000000000028 0.0000000000000000 F F F	0.3999999999999986 0.2000000000000028 0.0000000000000000 F F F	0.3999999999999986 0.2000000000000028 0.0000000000000000 F F F
0.3999999999999986 0.3999999999999986 0.0000000000000000 F F F	0.3999999999999986 0.3999999999999986 0.0000000000000000 F F F	0.3999999999999986 0.3999999999999986 0.0000000000000000 F F F
0.3999999999999986 0.6000000000000014 0.0000000000000000 F F F	0.3999999999999986 0.6000000000000014 0.0000000000000000 F F F	0.3999999999999986 0.6000000000000014 0.0000000000000000 F F F
0.3999999999999986 0.7999999999999972 0.0000000000000000 F F F	0.3999999999999986 0.7999999999999972 0.0000000000000000 F F F	0.3999999999999986 0.7999999999999972 0.0000000000000000 F F F
0.6000000000000014 0.0000000000000000 0.0000000000000000 F F F	0.6000000000000014 0.0000000000000000 0.0000000000000000 F F F	0.6000000000000014 0.0000000000000000 0.0000000000000000 F F F
0.6000000000000014 0.2000000000000028 0.0000000000000000 F F F	0.6000000000000014 0.2000000000000028 0.0000000000000000 F F F	0.6000000000000014 0.2000000000000028 0.0000000000000000 F F F
0.6000000000000014 0.3999999999999986 0.0000000000000000 F F F	0.6000000000000014 0.3999999999999986 0.0000000000000000 F F F	0.6000000000000014 0.3999999999999986 0.0000000000000000 F F F
0.6000000000000014 0.6000000000000014 0.0000000000000000 F F F	0.6000000000000014 0.6000000000000014 0.0000000000000000 F F F	0.6000000000000014 0.6000000000000014 0.0000000000000000 F F F
0.6000000000000014 0.7999999999999972 0.0000000000000000 F F F	0.6000000000000014 0.7999999999999972 0.0000000000000000 F F F	0.6000000000000014 0.7999999999999972 0.0000000000000000 F F F
0.7999999999999972 0.0000000000000000 0.0000000000000000 F F F	0.7999999999999972 0.0000000000000000 0.0000000000000000 F F F	0.7999999999999972 0.0000000000000000 0.0000000000000000 F F F
0.7999999999999972 0.2000000000000028 0.0000000000000000 F F F	0.7999999999999972 0.2000000000000028 0.0000000000000000 F F F	0.7999999999999972 0.2000000000000028 0.0000000000000000 F F F
0.7999999999999972 0.3999999999999986 0.0000000000000000 F F F	0.7999999999999972 0.3999999999999986 0.0000000000000000 F F F	0.7999999999999972 0.3999999999999986 0.0000000000000000 F F F
0.7999999999999972 0.6000000000000014 0.0000000000000000 F F F	0.7999999999999972 0.6000000000000014 0.0000000000000000 F F F	0.7999999999999972 0.6000000000000014 0.0000000000000000 F F F
0.7999999999999972 0.7999999999999972 0.0000000000000000 F F F	0.7999999999999972 0.7999999999999972 0.0000000000000000 F F F	0.7999999999999972 0.7999999999999972 0.0000000000000000 F F F
0.2749572387050879 0.9309358175651332 0.1307200272892057 T T T	0.2745307534447609 0.9298610595608592 0.1309321554590475 T T T	0.2715003010494951 0.9267470601173382 0.1305958371233524 T T T
0.4666644456187115 0.7333340047990546 0.1368027474874722 T T T	0.4666644456187115 0.7333340047990546 0.1368027474874722 T T T	0.4666644456187115 0.7333340047990546 0.1368027474874722 T T T
0.6666644456187072 0.5333340047990589 0.1368027474874722 T T T	0.6666644456187072 0.5333340047990589 0.1368027474874722 T T T	0.6666644456187072 0.5333340047990589 0.1368027474874722 T T T
0.8751501080392288 0.3456020029828772 0.1357551308583945 T T T	0.8764478190503052 0.3482321636408392 0.1359267878986532 T T T	0.8854925864756757 0.3584626882077612 0.1381377935205611 T T T
0.2634815931144797 0.7336465136383843 0.1299711893578452 T T T	0.2630953566803464 0.7323686092923913 0.1308908454540787 T T T	0.2609672462036827 0.7285108702576125 0.1308641027294543 T T T
0.4739818706573261 0.9374439313533059 0.1337955913696540 T T T	0.4722172662871298 0.9366122270742764 0.1344089232323916 T T T	0.4697196902106006 0.9353155085750878 0.1345632605051716 T T T
0.4666644456187115 0.5333340047990589 0.1368027474874722 T T T	0.4666644456187115 0.5333340047990589 0.1368027474874722 T T T	0.4666644456187115 0.5333340047990589 0.1368027474874722 T T T
0.6666644456187072 0.7333340047990546 0.1368027474874722 T T T	0.6666644456187072 0.7333340047990546 0.1368027474874722 T T T	0.6666644456187072 0.7333340047990546 0.1368027474874722 T T T
0.8627555964959942 0.5345344312852757 0.1332729969493650 T T T	0.8617433249048382 0.5347563643495657 0.1338672991999574 T T T	0.8661339508299709 0.5401873980953772 0.1326392935679157 T T T
0.6690931560936799 0.3343155246559910 0.1311943589054069 T T T	0.6689819345952859 0.3339665236884587 0.1312835406777208 T T T	0.6733705730926021 0.3377098542206333 0.1320968696936541 T T T
0.2626812230223050 0.5320342042619988 0.1315451986884447 T T T	0.2631513669131710 0.5324724753512612 0.1308226213865315 T T T	0.2621232210770117 0.5286982534360950 0.1307874916080362 T T T
0.4691716079585302 0.3362475670303962 0.1340905848774796 T T T	0.4695337808317065 0.3365088954526411 0.1342129052315802 T T T	0.4705838244007388 0.3371550897602982 0.1345849529866167 T T T
0.2718924470193333 0.3378054394829134 0.1299484369986734 T T T	0.2724905011652489 0.3384957840906558 0.1304537453366072 T T T	0.2706427422541151 0.3364491697463526 0.1301755418519660 T T T
0.6696488612914034 0.9358158570144874 0.1334306696769441 T T T	0.6674108453579601 0.9353434886998487 0.1344972299225069 T T T	0.6653122867565464 0.9353583694023820 0.1348431000932789 T T T
0.8619844817741069 0.7316301092606330 0.1352203887486433 T T T	0.8615102481353439 0.7323146450823637 0.1353568477072525 T T T	0.8633471678512129 0.7342849411670848 0.1350017022026100 T T T
0.8616993367101874 0.9290779634566980 0.1321924273005906 T T T	0.8608358436417799 0.9291054021357308 0.1324960602512162 T T T	0.8591208991520473 0.9290516775848724 0.1333393279338262 T T T
0.3487031337339976 0.8666911791373820 0.2626600332986361 T T T	0.3428225009847072 0.8618567712748081 0.2646348124972653 T T T	0.3356673867822670 0.8548841632160956 0.2631420888549333 T T T
0.5333313180231087 0.6666662410030355 0.2736054949749445 T T T	0.5333313180231087 0.6666662410030355 0.2736054949749445 T T T	0.5333313180231087 0.6666662410030355 0.2736054949749445 T T T
0.3270136069881073 0.6682509215751307 0.2582034379080965 T T T	0.3316553813241094 0.6682028583502269 0.2585970803972064 T T T	0.3223541356273887 0.6559672015897690 0.2591199776152909 T T T
0.5258094641424575 0.4654854835422800 0.2699303932074159 T T T	0.5236197407222551 0.4652037606364730 0.2707541823322207 T T T	0.5327029086500313 0.4686016386819827 0.2715563329194058 T T T
0.3237140244110364 0.4614854223111029 0.2667075981255879 T T T	0.3225122283220377 0.4646858744092153 0.2672150837109139 T T T	0.3343236368247049 0.4627197970938926 0.2636587631058437 T T T
0.5448858786777627 0.8715671824697988 0.2703917919363797 T T T	0.5397395889743234 0.8684213109217959 0.2710582739617974 T T T	0.5336770292007164 0.8660304875780103 0.2705636088116523 T T T
0.7246765428582623 0.6607565931524024 0.2710393068565731 T T T	0.7241555910629394 0.6601422972662520 0.2717666323089726 T T T	0.7288041854313724 0.6661898199484104 0.2697720847736219 T T T
0.7311235721136649 0.8605993506756376 0.2680183003764355 T T T	0.7291023793183443 0.8595838509271293 0.2681348719960618 T T T	0.7248038354040672 0.8612572139197083 0.2691747153781587 T T T
0.7207303941835592 0.4627082748066372 0.2619705153250779 T T T	0.7161362877696540 0.4604728123941494 0.2626990162062761 T T T	0.7309323156604179 0.4718176755690248 0.2615816036231264 T T T
0.4043436858119964 0.7693529867835289 0.3925332188421380 T T T	0.3993094549602701 0.7736079410141741 0.3933126451651265 T T T	0.3880036425476195 0.7703267915496561 0.3909219761730294 T T T
0.2643974497590707 0.5400342043884944 0.3907244453060288 T T T	0.2706285958830123 0.5408098449356201 0.3967147580905991 T T T	0.2565943078318819 0.5285378264110914 0.3883273317117349 T T T
0.5991413867677747 0.7827393555644692 0.4029587445011084 T T T	0.5962160482939394 0.7801986035602223 0.4031410513077281 T T T	0.5851913621810022 0.7804995176203077 0.4037801913148893 T T T
0.4653714224704237 0.5410732536101805 0.3978640343613609 T T T	0.4665785637422857 0.5449306454520448 0.3996123318659107 T T T	0.4570065048601006 0.5405790081257892 0.3976162538796837 T T T
0.4274329279271105 0.6530773893959864 0.5043931175693327 T T T	0.4255346470464081 0.6583142936579496 0.5055330531912018 T T T	0.4133944194189720 0.6523824029256242 0.5007014391540586 T T T
0.1516565105472588 0.3984066741818298 0.3134586437001274 T T T	0.1514108077489530 0.4070519444255158 0.3216997063256145 T T T	0.0720475180117184 0.4076334290490687 0.3819724008632919 T T T
0.0755438136805919 0.4183180571402835 0.2556512097609271 T T T	0.0734494975317682 0.4285339929330197 0.2650052332992077 T T T	0.0221089733606538 0.4327233689726996 0.3072964203584352 T T T
0.0229727937080223 0.2359409269719859 0.3139386914057656 T T T	0.0168623305830347 0.2493232267963118 0.3332586587035923 T T T	0.0254269973788378 0.2539632798219378 0.3326842166971006 T T T
0.2621399879992259 0.6715647057225248 0.3624420414101656 T T T	0.1449965222050245 0.5204368281214775 0.4115591152718436 T T T	0.0613943106499491 0.4436675579005697 0.4445334496976969 T T T
0.1065282206019879 0.5118506484214487 0.2352913199894024 T T T	0.1023278448725197 0.5227139395564944 0.2483799283185678 T T T	0.0251686380002753 0.5200633870353409 0.3049942173581585 T T T
0.1012729559487974 0.2824485251211821 0.3439176856858405 T T T	0.1014423500522688 0.2929797104309962 0.3530695524907151 T T T	0.0538045319981566 0.2919510883861496 0.3902116132352266 T T T
0.9700699706660176 0.3330894831738468 0.2325332523352996 T T T	0.9744353972614448 0.3420535793512579 0.2361307660873716 T T T	0.9739612498582289 0.3563564328914779 0.2451775995757434 T T T

Table S11. Atomic coordinates of IS, TS, and FS of Reaction 5 listed in Table S1.

Reaction 6: *COH–CHO+ H^{+}+e^{-}→*C–CHO on Cu-DAN via surface *H transfer		
IS	TS	FS
Cu C H O	Cu C H O	Cu C H O
1.00000000000000	1.00000000000000	1.00000000000000
12.8585004805000001 0.0000000000000000 0.0000000000000000	12.8585004805000001 0.0000000000000000 0.0000000000000000	12.8585004805000001 0.0000000000000000 0.0000000000000000
-6.4291002405000004 11.1357880710000003 0.0000000000000000	-6.4291002405000004 11.1357880710000003 0.0000000000000000	-6.4291002405000004 11.1357880710000003 0.0000000000000000
0.0000000000000000 0.0000000000000000 15.2994003295999992	0.0000000000000000 0.0000000000000000 15.2994003295999992	0.0000000000000000 0.0000000000000000 15.2994003295999992
Cu C H O	Cu C H O	Cu C H O
55 2 3 2	55 2 3 2	55 2 3 2
Selective dynamics	Selective dynamics	Selective dynamics
Direct	Direct	Direct
0.0000000000000000 0.0000000000000000 0.0000000000000000 F F F	0.0000000000000000 0.0000000000000000 0.0000000000000000 F F F	0.0000000000000000 0.0000000000000000 0.0000000000000000 F F F
0.0000000000000000 0.2000000000000028 0.0000000000000000 F F F	0.0000000000000000 0.2000000000000028 0.0000000000000000 F F F	0.0000000000000000 0.2000000000000028 0.0000000000000000 F F F
0.0000000000000000 0.3999999999999986 0.0000000000000000 F F F	0.0000000000000000 0.3999999999999986 0.0000000000000000 F F F	0.0000000000000000 0.3999999999999986 0.0000000000000000 F F F
0.0000000000000000 0.6000000000000014 0.0000000000000000 F F F	0.0000000000000000 0.6000000000000014 0.0000000000000000 F F F	0.0000000000000000 0.6000000000000014 0.0000000000000000 F F F
0.0000000000000000 0.7999999999999972 0.0000000000000000 F F F	0.0000000000000000 0.7999999999999972 0.0000000000000000 F F F	0.0000000000000000 0.7999999999999972 0.0000000000000000 F F F
0.2000000000000028 0.0000000000000000 0.0000000000000000 F F F	0.2000000000000028 0.0000000000000000 0.0000000000000000 F F F	0.2000000000000028 0.0000000000000000 0.0000000000000000 F F F
0.2000000000000028 0.2000000000000028 0.0000000000000000 F F F	0.2000000000000028 0.2000000000000028 0.0000000000000000 F F F	0.2000000000000028 0.2000000000000028 0.0000000000000000 F F F
0.2000000000000028 0.3999999999999986 0.0000000000000000 F F F	0.2000000000000028 0.3999999999999986 0.0000000000000000 F F F	0.2000000000000028 0.3999999999999986 0.0000000000000000 F F F
0.2000000000000028 0.6000000000000014 0.0000000000000000 F F F	0.2000000000000028 0.6000000000000014 0.0000000000000000 F F F	0.2000000000000028 0.6000000000000014 0.0000000000000000 F F F
0.2000000000000028 0.7999999999999972 0.0000000000000000 F F F	0.2000000000000028 0.7999999999999972 0.0000000000000000 F F F	0.2000000000000028 0.7999999999999972 0.0000000000000000 F F F
0.3999999999999986 0.0000000000000000 0.0000000000000000 F F F	0.3999999999999986 0.0000000000000000 0.0000000000000000 F F F	0.3999999999999986 0.0000000000000000 0.0000000000000000 F F F
0.3999999999999986 0.2000000000000028 0.0000000000000000 F F F	0.3999999999999986 0.2000000000000028 0.0000000000000000 F F F	0.3999999999999986 0.2000000000000028 0.0000000000000000 F F F
0.3999999999999986 0.3999999999999986 0.0000000000000000 F F F	0.3999999999999986 0.3999999999999986 0.0000000000000000 F F F	0.3999999999999986 0.3999999999999986 0.0000000000000000 F F F
0.3999999999999986 0.6000000000000014 0.0000000000000000 F F F	0.3999999999999986 0.6000000000000014 0.0000000000000000 F F F	0.3999999999999986 0.6000000000000014 0.0000000000000000 F F F
0.3999999999999986 0.7999999999999972 0.0000000000000000 F F F	0.3999999999999986 0.7999999999999972 0.0000000000000000 F F F	0.3999999999999986 0.7999999999999972 0.0000000000000000 F F F
0.6000000000000014 0.0000000000000000 0.0000000000000000 F F F	0.6000000000000014 0.0000000000000000 0.0000000000000000 F F F	0.6000000000000014 0.0000000000000000 0.0000000000000000 F F F
0.6000000000000014 0.2000000000000028 0.0000000000000000 F F F	0.6000000000000014 0.2000000000000028 0.0000000000000000 F F F	0.6000000000000014 0.2000000000000028 0.0000000000000000 F F F
0.6000000000000014 0.3999999999999986 0.0000000000000000 F F F	0.6000000000000014 0.3999999999999986 0.0000000000000000 F F F	0.6000000000000014 0.3999999999999986 0.0000000000000000 F F F
0.6000000000000014 0.6000000000000014 0.0000000000000000 F F F	0.6000000000000014 0.6000000000000014 0.0000000000000000 F F F	0.6000000000000014 0.6000000000000014 0.0000000000000000 F F F
0.6000000000000014 0.7999999999999972 0.0000000000000000 F F F	0.6000000000000014 0.7999999999999972 0.0000000000000000 F F F	0.6000000000000014 0.7999999999999972 0.0000000000000000 F F F
0.7999999999999972 0.0000000000000000 0.0000000000000000 F F F	0.7999999999999972 0.0000000000000000 0.0000000000000000 F F F	0.7999999999999972 0.0000000000000000 0.0000000000000000 F F F
0.7999999999999972 0.2000000000000028 0.0000000000000000 F F F	0.7999999999999972 0.2000000000000028 0.0000000000000000 F F F	0.7999999999999972 0.2000000000000028 0.0000000000000000 F F F
0.7999999999999972 0.3999999999999986 0.0000000000000000 F F F	0.7999999999999972 0.3999999999999986 0.0000000000000000 F F F	0.7999999999999972 0.3999999999999986 0.0000000000000000 F F F
0.7999999999999972 0.6000000000000014 0.0000000000000000 F F F	0.7999999999999972 0.6000000000000014 0.0000000000000000 F F F	0.7999999999999972 0.6000000000000014 0.0000000000000000 F F F
0.7999999999999972 0.7999999999999972 0.0000000000000000 F F F	0.7999999999999972 0.7999999999999972 0.0000000000000000 F F F	0.7999999999999972 0.7999999999999972 0.0000000000000000 F F F
0.2749572387050879 0.9309358175651332 0.1307200272892057 T T T	0.2766753296932336 0.9305738362641733 0.1285479763820661 T T T	0.2731829895066407 0.9333091507936000 0.1304414487981246 T T T
0.4666644456187115 0.7333340047990546 0.1368027474874722 T T T	0.4666644456031577 0.7333340047990546 0.1368027475005462 T T T	0.4666644456031577 0.7333340047990546 0.1368027475005462 T T T
0.6666644456187072 0.5333340047990589 0.1368027474874722 T T T	0.6666644456031534 0.5333340047990589 0.1368027475005462 T T T	0.6666644456031534 0.5333340047990589 0.1368027475005462 T T T
0.8751501080392288 0.3456020029828772 0.1357551308583945 T T T	0.8825178758874870 0.3525854871721421 0.1335193707457805 T T T	0.8844839633723388 0.3595133270023359 0.1386039072340100 T T T
0.2634815931144797 0.7336465136383843 0.1299711893578452 T T T	0.2690355179355564 0.7354193947586154 0.1284383926880395 T T T	0.2662556927913377 0.7382295295453830 0.1288687148043082 T T T
0.4739818706573261 0.9374439313533059 0.1337955913696540 T T T	0.4765384801487045 0.9401663796921227 0.1286946936902424 T T T	0.4713733706479514 0.9377636340362704 0.1334800918747687 T T T
0.4666644456187115 0.5333340047990589 0.1368027474874722 T T T	0.4666644456031577 0.5333340047990589 0.1368027475005462 T T T	0.4666644456031577 0.5333340047990589 0.1368027475005462 T T T
0.6666644456187072 0.7333340047990546 0.1368027474874722 T T T	0.6666644456031534 0.7333340047990546 0.1368027475005462 T T T	0.6666644456031534 0.7333340047990546 0.1368027475005462 T T T
0.8627555964959942 0.5345344312852757 0.1332729969493650 T T T	0.8638458349467797 0.5362156394479385 0.1276778718818434 T T T	0.8663609764172332 0.5403054774470335 0.1321109224436171 T T T
0.6690931560936799 0.3343155246559910 0.1311943589054069 T T T	0.6696596500683327 0.3353545623732757 0.1293389679103782 T T T	0.6741852037629568 0.3372542021662746 0.1319746830994769 T T T
0.2626812230223050 0.5320342042619988 0.1315451986884447 T T T	0.2639728315250096 0.5288142885091887 0.1356974297276992 T T T	0.2584246429098896 0.5264021517941578 0.1381397102527550 T T T
0.4691716079585302 0.3362475670303962 0.1340905848774796 T T T	0.4658965399515722 0.3313891099007478 0.1275101511184360 T T T	0.4706689109523867 0.3358123845465387 0.1341964295244632 T T T
0.2718924470193333 0.3378054394829134 0.1299484369986734 T T T	0.2692722121463973 0.3344301523069930 0.1259006510015723 T T T	0.2727390620021706 0.3345459689412506 0.1285262544267302 T T T
0.6696488612914034 0.9358158570144874 0.1334306696769441 T T T	0.6745982153229093 0.9406879577370759 0.1293720044095323 T T T	0.6670577816128642 0.9378383417611704 0.1343091345522208 T T T
0.8619844817741069 0.7316301092606330 0.1352203887486433 T T T	0.8612692360833023 0.7316951596723982 0.1324398654829437 T T T	0.8619190900230567 0.7343465570564935 0.1354399438248876 T T T
0.8616993367101874 0.9290779634566980 0.1321924273005906 T T T	0.8633699998491053 0.9307900696719642 0.1257579365707784 T T T	0.8597123765888207 0.9301310706927138 0.1322659704099688 T T T
0.3487031337339976 0.8666911791373820 0.2626600332986361 T T T	0.3564710867139240 0.8679721741541542 0.2603397457550969 T T T	0.3425843518699067 0.8685008147625205 0.2638015242913752 T T T
0.5333313180231087 0.6666662410030355 0.2736054949749445 T T T	0.5333313179997745 0.6666662410030355 0.2736054950010853 T T T	0.5333313179997745 0.6666662410030355 0.2736054950010853 T T T
0.3270136069881073 0.6682509215751307 0.2582034379080965 T T T	0.3285800259525402 0.6681549149528223 0.2654357294024692 T T T	0.3136308030884463 0.6680522452827820 0.2611681577493407 T T T
0.5258094641424575 0.4654854835422800 0.2699303932074159 T T T	0.5308079193505648 0.4495386401555166 0.2612173100899469 T T T	0.5363043209664436 0.4647364600803867 0.2696437845320231 T T T
0.3237140244110364 0.4614854223111029 0.2667075981255879 T T T	0.3339550799094866 0.4461779770324981 0.2651353759580388 T T T	0.3380988002768040 0.4498551122022579 0.2671475477334174 T T T
0.5448858786777627 0.8715671824697988 0.2703917919363797 T T T	0.5501988717313040 0.8747227463490361 0.2592893325191525 T T T	0.5342127015013701 0.8657100163737939 0.2676594962445215 T T T
0.7246765428582623 0.6607565931524024 0.2710393068565731 T T T	0.7217316969738050 0.6580612293872363 0.2755505595713146 T T T	0.7261449591691064 0.6641469536817476 0.2721246778975461 T T T
0.7311235721136649 0.8605993506756376 0.2680183003764355 T T T	0.7395279193836402 0.8645719492200001 0.2622387331190765 T T T	0.7264964611795538 0.8624672713139764 0.2665104916905593 T T T
0.7207303941835592 0.4627082748066372 0.2619705153250779 T T T	0.7379710274147797 0.4708707395894365 0.2600035579334374 T T T	0.7320931981736686 0.4705343972802679 0.2613948335366666 T T T
0.4043436858119964 0.7693529867835289 0.3925332188421380 T T T	0.4642805678061777 0.7611929955249082 0.3894082494869124 T T T	0.4018171688233069 0.7803846943536763 0.3948180766515512 T T T
0.2643974497590707 0.5400342043884944 0.3907244453060288 T T T	0.3012876970598128 0.5412287359158725 0.3903915345975264 T T T	0.3096123553011442 0.5559887384203561 0.3872244870182152 T T T
0.5991413867677747 0.7827393555644692 0.4029587445011084 T T T	0.6588894853183263 0.7503228439989940 0.3985042999028963 T T T	0.5954475503560787 0.7853106338933865 0.4002240261359933 T T T
0.4653714224704237 0.5410732536101805 0.3978640343613609 T T T	0.4988640785229919 0.5297027037692956 0.3908878612524163 T T T	0.4981028920281349 0.5508356344133490 0.4025813884328926 T T T
0.4274329279271105 0.6530773893959864 0.5043931175693327 T T T	0.5004604734743765 0.6566398811214782 0.5042387037989258 T T T	0.4410876152178007 0.6620346869033661 0.5055085191585839 T T T
0.1516565105472588 0.3984066741818298 0.3134586437001274 T T T	0.2097052112319146 0.4936707765450665 0.2717356574268423 T T T	0.2113095020164276 0.4929046182056642 0.2752435683784388 T T T
0.0755438136805919 0.4183180571402835 0.2556512097609271 T T T	0.0834071252601503 0.4478571288219834 0.2686715299041044 T T T	0.0842445696412110 0.4249913725959378 0.2790954520835555 T T T
0.0229727937080223 0.2359409269719859 0.3139386914057656 T T T	0.1918767416035243 0.5286409430758371 0.5672485571991110 T T T	0.0985740452177204 0.2976842838054491 0.5087917202980373 T T T
0.2621399879992259 0.6715647057225248 0.3624420414101656 T T T	0.2920395873934075 0.6558292586785712 0.5331759729430655 T T T	0.9978521539811700 0.2808425083522703 0.5717209242337030 T T T
0.1065282206019879 0.5118506484214487 0.2352913199894024 T T T	0.0445501097416646 0.4832109567453968 0.3175952775914572 T T T	0.0419807250117815 0.4134863955495169 0.3444463004654241 T T T
0.1012729559487974 0.2824485251211821 0.3439176856858405 T T T	0.2645936488524119 0.5697032612334356 0.5315001253576105 T T T	0.0783814346865220 0.3397494110207233 0.5524948175668991 T T T
0.9700699706660176 0.3330894831738468 0.2325332523352996 T T T	0.0110513984104420 0.3722935983693709 0.2125094123339619 T T T	0.0144483761403342 0.3754435910716315 0.2137013755827944 T T T

Table S12. Atomic coordinates of IS, TS, and FS of Reaction 7 listed in Table S1.

Reaction 7: *CHOH–CHO+ H^++e^-→*CH_2OH–CHO on Cu-DAN via surface *H transfer

IS	TS	FS
Cu C H O<br>1.00000000000000<br>12.8585004805000001 0.0000000000000000 0.0000000000000000<br>-6.4291002405000004 11.1357880710000003 0.0000000000000000<br>0.0000000000000000 0.0000000000000000 15.2994003295999992<br>Cu C H O<br>55 2 4 2<br>Selective dynamics<br>Direct<br>0.0000000000000000 0.0000000000000000 0.0000000000000000 F F F<br>0.0000000000000000 0.2000000000000028 0.0000000000000000 F F F<br>0.0000000000000000 0.3999999999999986 0.0000000000000000 F F F<br>0.0000000000000000 0.6000000000000014 0.0000000000000000 F F F<br>0.0000000000000000 0.7999999999999972 0.0000000000000000 F F F<br>0.2000000000000028 0.0000000000000000 0.0000000000000000 F F F<br>0.2000000000000028 0.2000000000000028 0.0000000000000000 F F F<br>0.2000000000000028 0.3999999999999986 0.0000000000000000 F F F<br>0.2000000000000028 0.6000000000000014 0.0000000000000000 F F F<br>0.2000000000000028 0.7999999999999972 0.0000000000000000 F F F<br>0.3999999999999986 0.0000000000000000 0.0000000000000000 F F F<br>0.3999999999999986 0.2000000000000028 0.0000000000000000 F F F<br>0.3999999999999986 0.3999999999999986 0.0000000000000000 F F F<br>0.3999999999999986 0.6000000000000014 0.0000000000000000 F F F<br>0.3999999999999986 0.7999999999999972 0.0000000000000000 F F F<br>0.6000000000000014 0.0000000000000000 0.0000000000000000 F F F<br>0.6000000000000014 0.2000000000000028 0.0000000000000000 F F F<br>0.6000000000000014 0.3999999999999986 0.0000000000000000 F F F<br>0.6000000000000014 0.6000000000000014 0.0000000000000000 F F F<br>0.6000000000000014 0.7999999999999972 0.0000000000000000 F F F<br>0.7999999999999972 0.0000000000000000 0.0000000000000000 F F F<br>0.7999999999999972 0.2000000000000028 0.0000000000000000 F F F<br>0.7999999999999972 0.3999999999999986 0.0000000000000000 F F F<br>0.7999999999999972 0.6000000000000014 0.0000000000000000 F F F<br>0.7999999999999972 0.7999999999999972 0.0000000000000000 F F F<br>0.2768842633337026 0.9263701058982168 0.1319859884029393 T T T<br>0.4666644456187115 0.7333340047990546 0.1368027474874722 T T T<br>0.6666644456187072 0.5333340047990589 0.1368027474874722 T T T<br>0.8814619781179763 0.3538842400109978 0.1368996987185896 T T T<br>0.2601853987481614 0.7264692982260241 0.1290711299568778 T T T<br>0.4747730081179445 0.9368374878746980 0.1346797826198278 T T T<br>0.4666644456187115 0.5333340047990589 0.1368027474874722 T T T<br>0.6666644456187072 0.7333340047990546 0.1368027474874722 T T T<br>0.8637377554744010 0.5373618089428061 0.1333510255940112 T T T<br>0.6708415204181082 0.3353572229363184 0.1315163021463490 T T T<br>0.2614728532354219 0.5264142527478071 0.1290610850474772 T T T<br>0.4705794933891757 0.3372568091794533 0.1350712605420796 T T T<br>0.2711203734909837 0.3345145866781458 0.1300078827152449 T T T<br>0.6703683354619134 0.9365236828446027 0.1338531443911362 T T T<br>0.8628075100594271 0.7327259958495609 0.1352999237448195 T T T<br>0.8621631386896529 0.9295123641366068 0.1317377457786914 T T T<br>0.3471235020908705 0.8492955649837179 0.2617632598223177 T T T<br>0.5333313180231087 0.6666662410030355 0.2736054949749445 T T T<br>0.3063759753547645 0.6426469006201850 0.2583423263221214 T T T<br>0.5290938731522471 0.4675224721323431 0.2731917346894284 T T T<br>0.3322299850869059 0.4608413538336575 0.2627001221647493 T T T<br>0.5506853405241698 0.8716207563631492 0.2698047312012992 T T T<br>0.7258497381077069 0.6614610828915424 0.2710229482919422 T T T<br>0.7353947009884829 0.8614700864028313 0.2679330252108892 T T T<br>0.7239013618062736 0.4657516328293147 0.2620240526413571 T T T<br>0.4065910308329426 0.7437584063430558 0.3873051505200079 T T T<br>0.2423698948233720 0.5118004580200001 0.3870726091475440 T T T<br>0.6081705903289361 0.7786286038786671 0.4047143548916508 T T T<br>0.4487997989080419 0.5456829510160216 0.3977033782873480 T T T<br>0.4425630559563447 0.6638301335319416 0.5139817829856133 T T T<br>0.0584748752810936 0.3934315041799267 0.3828325202740581 T T T<br>0.0103772485872298 0.4204575313371999 0.3081850986977471 T T T<br>0.0107704016584024 0.2398159629369817 0.3326331272267211 T T T<br>0.0141451595957280 0.5081606975800880 0.3065001061184824 T T T<br>0.0474033490884412 0.4288318861851282 0.4455934243544923 T T T<br>0.2874916578510464 0.7388447914169152 0.3390496791716815 T T T<br>0.0384597508792987 0.2768717907704269 0.3904907920686352 T T T<br>0.9631232522787602 0.3452687933413510 0.2450947157266563 T T T	Cu C H O<br>1.00000000000000<br>12.8585004805000001 0.0000000000000000 0.0000000000000000<br>-6.4291002405000004 11.1357880710000003 0.0000000000000000<br>0.0000000000000000 0.0000000000000000 15.2994003295999992<br>Cu C H O<br>55 2 4 2<br>Selective dynamics<br>Direct<br>0.0000000000000000 0.0000000000000000 0.0000000000000000 F F F<br>0.0000000000000000 0.2000000000000028 0.0000000000000000 F F F<br>0.0000000000000000 0.3999999999999986 0.0000000000000000 F F F<br>0.0000000000000000 0.6000000000000014 0.0000000000000000 F F F<br>0.0000000000000000 0.7999999999999972 0.0000000000000000 F F F<br>0.2000000000000028 0.0000000000000000 0.0000000000000000 F F F<br>0.2000000000000028 0.2000000000000028 0.0000000000000000 F F F<br>0.2000000000000028 0.3999999999999986 0.0000000000000000 F F F<br>0.2000000000000028 0.6000000000000014 0.0000000000000000 F F F<br>0.2000000000000028 0.7999999999999972 0.0000000000000000 F F F<br>0.3999999999999986 0.0000000000000000 0.0000000000000000 F F F<br>0.3999999999999986 0.2000000000000028 0.0000000000000000 F F F<br>0.3999999999999986 0.3999999999999986 0.0000000000000000 F F F<br>0.3999999999999986 0.6000000000000014 0.0000000000000000 F F F<br>0.3999999999999986 0.7999999999999972 0.0000000000000000 F F F<br>0.6000000000000014 0.0000000000000000 0.0000000000000000 F F F<br>0.6000000000000014 0.2000000000000028 0.0000000000000000 F F F<br>0.6000000000000014 0.3999999999999986 0.0000000000000000 F F F<br>0.6000000000000014 0.6000000000000014 0.0000000000000000 F F F<br>0.6000000000000014 0.7999999999999972 0.0000000000000000 F F F<br>0.7999999999999972 0.0000000000000000 0.0000000000000000 F F F<br>0.7999999999999972 0.2000000000000028 0.0000000000000000 F F F<br>0.7999999999999972 0.3999999999999986 0.0000000000000000 F F F<br>0.7999999999999972 0.6000000000000014 0.0000000000000000 F F F<br>0.7999999999999972 0.7999999999999972 0.0000000000000000 F F F<br>0.2762090528019860 0.9268521388648761 0.1317638917295824 T T T<br>0.4666644456187115 0.7333340047990546 0.1368027474874722 T T T<br>0.6666644456187072 0.5333340047990589 0.1368027474874722 T T T<br>0.8703264889433930 0.3422857941158472 0.1328180779170704 T T T<br>0.2630095656856550 0.7287482023117032 0.1316052816097734 T T T<br>0.4755908123507301 0.9371001799443269 0.1334545252434286 T T T<br>0.4666644456187115 0.5333340047990589 0.1368027474874722 T T T<br>0.6666644456187072 0.7333340047990546 0.1368027474874722 T T T<br>0.8646315669923613 0.5357049709345892 0.1321531409356810 T T T<br>0.6680686702925908 0.3362511172446067 0.1324603522328909 T T T<br>0.2637944727791444 0.5295598182287081 0.1324323410466271 T T T<br>0.4674579154400220 0.3348801756254912 0.1329276312739459 T T T<br>0.2710199210436064 0.3370806584627956 0.1307955722180187 T T T<br>0.6726075397700411 0.9377534037223337 0.1322313141472142 T T T<br>0.8616954165280170 0.7314932845908612 0.1372380002863965 T T T<br>0.8648828255616996 0.9303254275175301 0.1300873386837008 T T T<br>0.3534834312713863 0.8588307018021855 0.2626842587247638 T T T<br>0.5333313180231087 0.6666662410030355 0.2736054949749445 T T T<br>0.3159687633997231 0.6519072136302236 0.2627231830978201 T T T<br>0.5312770310423403 0.4611885128658791 0.2679304296801592 T T T<br>0.3372653415209746 0.4619763178925494 0.2670688420222474 T T T<br>0.5533085711333935 0.8744891349460522 0.2676977154254172 T T T<br>0.7220897885771906 0.6607956461423442 0.2744100787988743 T T T<br>0.7399278882453902 0.8642613896619945 0.2639810933742229 T T T<br>0.7315433240487996 0.4713090904572151 0.2621592205727845 T T T<br>0.4211720736183301 0.7730206848275349 0.3907187982000820 T T T<br>0.3145664627405325 0.5548241431310460 0.3996842103816325 T T T<br>0.6208620112734150 0.7899764210842715 0.3983080798040624 T T T<br>0.4990293754584899 0.5480678754139806 0.4003350428801752 T T T<br>0.4709809576730417 0.6730474276637771 0.5079214703528898 T T T<br>0.0518748909469833 0.3664581257071153 0.3646459738246945 T T T<br>0.0153949889783266 0.4021616606732116 0.2909944989881934 T T T<br>0.9645742853723148 0.2058045596393630 0.3278408266958671 T T T<br>0.0452496395252894 0.4978086066043228 0.2816615093411201 T T T<br>0.0976276306173631 0.4260435523418025 0.4197619576974912 T T T<br>0.2748063932238528 0.6610658254367560 0.3716693985659347 T T T<br>0.0124707919147178 0.2484965739910724 0.3797247140322691 T T T<br>0.9420286545880943 0.3207529033713045 0.2354140146984040 T T T	Cu C H O<br>1.00000000000000<br>12.8585004805000001 0.0000000000000000 0.0000000000000000<br>-6.4291002405000004 11.1357880710000003 0.0000000000000000<br>0.0000000000000000 0.0000000000000000 15.2994003295999992<br>Cu C H O<br>55 2 4 2<br>Selective dynamics<br>Direct<br>0.0000000000000000 0.0000000000000000 0.0000000000000000 F F F<br>0.0000000000000000 0.2000000000000028 0.0000000000000000 F F F<br>0.0000000000000000 0.3999999999999986 0.0000000000000000 F F F<br>0.0000000000000000 0.6000000000000014 0.0000000000000000 F F F<br>0.0000000000000000 0.7999999999999972 0.0000000000000000 F F F<br>0.2000000000000028 0.0000000000000000 0.0000000000000000 F F F<br>0.2000000000000028 0.2000000000000028 0.0000000000000000 F F F<br>0.2000000000000028 0.3999999999999986 0.0000000000000000 F F F<br>0.2000000000000028 0.6000000000000014 0.0000000000000000 F F F<br>0.2000000000000028 0.7999999999999972 0.0000000000000000 F F F<br>0.3999999999999986 0.0000000000000000 0.0000000000000000 F F F<br>0.3999999999999986 0.2000000000000028 0.0000000000000000 F F F<br>0.3999999999999986 0.3999999999999986 0.0000000000000000 F F F<br>0.3999999999999986 0.6000000000000014 0.0000000000000000 F F F<br>0.3999999999999986 0.7999999999999972 0.0000000000000000 F F F<br>0.6000000000000014 0.0000000000000000 0.0000000000000000 F F F<br>0.6000000000000014 0.2000000000000028 0.0000000000000000 F F F<br>0.6000000000000014 0.3999999999999986 0.0000000000000000 F F F<br>0.6000000000000014 0.6000000000000014 0.0000000000000000 F F F<br>0.6000000000000014 0.7999999999999972 0.0000000000000000 F F F<br>0.7999999999999972 0.0000000000000000 0.0000000000000000 F F F<br>0.7999999999999972 0.2000000000000028 0.0000000000000000 F F F<br>0.7999999999999972 0.3999999999999986 0.0000000000000000 F F F<br>0.7999999999999972 0.6000000000000014 0.0000000000000000 F F F<br>0.7999999999999972 0.7999999999999972 0.0000000000000000 F F F<br>0.2743659231457726 0.9251492809960306 0.1320908533464791 T T T<br>0.4666644456187115 0.7333340047990546 0.1368027474874722 T T T<br>0.6666644456187072 0.5333340047990589 0.1368027474874722 T T T<br>0.8629821939165943 0.3368582690513640 0.1299021977515819 T T T<br>0.2632242062210838 0.7272196741781962 0.1342780815550796 T T T<br>0.4719085474449865 0.9356854878291168 0.1340998310271381 T T T<br>0.4666644456187115 0.5333340047990589 0.1368027474874722 T T T<br>0.6666644456187072 0.7333340047990546 0.1368027474874722 T T T<br>0.8629579665638079 0.5338672105917504 0.1332002780098050 T T T<br>0.6662190354911330 0.3365108783142655 0.1332405896921454 T T T<br>0.2610460080355665 0.5297801602350909 0.1324718059937467 T T T<br>0.4675323230089852 0.3378386289051335 0.1354040497204658 T T T<br>0.2689612785744467 0.3356469257745357 0.1291832879302558 T T T<br>0.6701235750021315 0.9391753506313359 0.1324182377192616 T T T<br>0.8622207895055293 0.7325079704610094 0.1355079402532195 T T T<br>0.8644301717613339 0.9311563892425198 0.1290514155560016 T T T<br>0.3467311223532172 0.8524901290073614 0.2662337317267019 T T T<br>0.5333313180231087 0.6666662410030355 0.2736054949749445 T T T<br>0.3260430621395171 0.6520693875442434 0.2669808236040775 T T T<br>0.5318989195102770 0.4692809868689175 0.2724424401526956 T T T<br>0.3306874909437209 0.4594102559123397 0.2607151231805501 T T T<br>0.5474397320411839 0.8731070523906956 0.2667127510620786 T T T<br>0.7304338420316236 0.6676956653392224 0.2724625767460152 T T T<br>0.7408172829563247 0.8694043965865339 0.2604004569777698 T T T<br>0.7274325932197885 0.4719892652468760 0.2640083623697005 T T T<br>0.4232049117006613 0.7766828230537903 0.3963612748069681 T T T<br>0.3914115100534463 0.5719566652479930 0.3940603934536772 T T T<br>0.6287234739144756 0.8090938234779821 0.3937976938634589 T T T<br>0.5968728549970976 0.6031995480160363 0.4053099994577300 T T T<br>0.5090284357727921 0.6919901526091011 0.5122887127973696 T T T<br>0.0090509658467734 0.3420127886697791 0.3849727770203366 T T T<br>0.9831454433678312 0.3800863533159702 0.3000203163938397 T T T<br>0.9116481838910628 0.1792635980116119 0.3422959442962076 T T T<br>0.0232692632539337 0.4777496885958367 0.2876739370030019 T T T<br>0.9796156728810628 0.3820705262083626 0.4371362649340806 T T T<br>0.1087519519254265 0.3881817541679150 0.3909951857726459 T T T<br>0.9562496071253208 0.2174950510887692 0.3959795911433197 T T T<br>0.9180357014301158 0.3043672677706052 0.2449108564294740 T T T

Table S13. Atomic coordinates of IS, TS, and FS of Reaction 8 listed in Table S1.

Reaction 8: $^*CH_2OH–CHO + H^+ + e^- \rightarrow {}^*CH_2OH–CH_2O$ on Cu-DAN via surface *H transfer

IS	TS	FS
Cu C H O	Cu C H O	Cu C H O
1.00000000000000	1.00000000000000	1.00000000000000
12.8585004805000001 0.0000000000000000 0.0000000000000000	12.8585004805000001 0.0000000000000000 0.0000000000000000	12.8585004805000001 0.0000000000000000 0.0000000000000000
-6.4291002405000004 11.1357880710000003 0.0000000000000000	-6.4291002405000004 11.1357880710000003 0.0000000000000000	-6.4291002405000004 11.1357880710000003 0.0000000000000000
0.0000000000000000 0.0000000000000000 15.2994003295999992	0.0000000000000000 0.0000000000000000 15.2994003295999992	0.0000000000000000 0.0000000000000000 15.2994003295999992
Cu C H O	Cu C H O	Cu C H O
55 2 5 2	55 2 5 2	55 2 5 2
Selective dynamics	Selective dynamics	Selective dynamics
Direct	Direct	Direct
0.0000000000000000 0.0000000000000000 0.0000000000000000 F F F	0.0000000000000000 0.0000000000000000 0.0000000000000000 F F F	0.0000000000000000 0.0000000000000000 0.0000000000000000 F F F
0.0000000000000000 0.2000000000000028 0.0000000000000000 F F F	0.0000000000000000 0.2000000000000028 0.0000000000000000 F F F	0.0000000000000000 0.2000000000000028 0.0000000000000000 F F F
0.0000000000000000 0.3999999999999986 0.0000000000000000 F F F	0.0000000000000000 0.3999999999999986 0.0000000000000000 F F F	0.0000000000000000 0.3999999999999986 0.0000000000000000 F F F
0.0000000000000000 0.6000000000000014 0.0000000000000000 F F F	0.0000000000000000 0.6000000000000014 0.0000000000000000 F F F	0.0000000000000000 0.6000000000000014 0.0000000000000000 F F F
0.0000000000000000 0.7999999999999972 0.0000000000000000 F F F	0.0000000000000000 0.7999999999999972 0.0000000000000000 F F F	0.0000000000000000 0.7999999999999972 0.0000000000000000 F F F
0.2000000000000028 0.0000000000000000 0.0000000000000000 F F F	0.2000000000000028 0.0000000000000000 0.0000000000000000 F F F	0.2000000000000028 0.0000000000000000 0.0000000000000000 F F F
0.2000000000000028 0.2000000000000028 0.0000000000000000 F F F	0.2000000000000028 0.2000000000000028 0.0000000000000000 F F F	0.2000000000000028 0.2000000000000028 0.0000000000000000 F F F
0.2000000000000028 0.3999999999999986 0.0000000000000000 F F F	0.2000000000000028 0.3999999999999986 0.0000000000000000 F F F	0.2000000000000028 0.3999999999999986 0.0000000000000000 F F F
0.2000000000000028 0.6000000000000014 0.0000000000000000 F F F	0.2000000000000028 0.6000000000000014 0.0000000000000000 F F F	0.2000000000000028 0.6000000000000014 0.0000000000000000 F F F
0.2000000000000028 0.7999999999999972 0.0000000000000000 F F F	0.2000000000000028 0.7999999999999972 0.0000000000000000 F F F	0.2000000000000028 0.7999999999999972 0.0000000000000000 F F F
0.3999999999999986 0.0000000000000000 0.0000000000000000 F F F	0.3999999999999986 0.0000000000000000 0.0000000000000000 F F F	0.3999999999999986 0.0000000000000000 0.0000000000000000 F F F
0.3999999999999986 0.2000000000000028 0.0000000000000000 F F F	0.3999999999999986 0.2000000000000028 0.0000000000000000 F F F	0.3999999999999986 0.2000000000000028 0.0000000000000000 F F F
0.3999999999999986 0.3999999999999986 0.0000000000000000 F F F	0.3999999999999986 0.3999999999999986 0.0000000000000000 F F F	0.3999999999999986 0.3999999999999986 0.0000000000000000 F F F
0.3999999999999986 0.6000000000000014 0.0000000000000000 F F F	0.3999999999999986 0.6000000000000014 0.0000000000000000 F F F	0.3999999999999986 0.6000000000000014 0.0000000000000000 F F F
0.3999999999999986 0.7999999999999972 0.0000000000000000 F F F	0.3999999999999986 0.7999999999999972 0.0000000000000000 F F F	0.3999999999999986 0.7999999999999972 0.0000000000000000 F F F
0.6000000000000014 0.0000000000000000 0.0000000000000000 F F F	0.6000000000000014 0.0000000000000000 0.0000000000000000 F F F	0.6000000000000014 0.0000000000000000 0.0000000000000000 F F F
0.6000000000000014 0.2000000000000028 0.0000000000000000 F F F	0.6000000000000014 0.2000000000000028 0.0000000000000000 F F F	0.6000000000000014 0.2000000000000028 0.0000000000000000 F F F
0.6000000000000014 0.3999999999999986 0.0000000000000000 F F F	0.6000000000000014 0.3999999999999986 0.0000000000000000 F F F	0.6000000000000014 0.3999999999999986 0.0000000000000000 F F F
0.6000000000000014 0.6000000000000014 0.0000000000000000 F F F	0.6000000000000014 0.6000000000000014 0.0000000000000000 F F F	0.6000000000000014 0.6000000000000014 0.0000000000000000 F F F
0.6000000000000014 0.7999999999999972 0.0000000000000000 F F F	0.6000000000000014 0.7999999999999972 0.0000000000000000 F F F	0.6000000000000014 0.7999999999999972 0.0000000000000000 F F F
0.7999999999999972 0.0000000000000000 0.0000000000000000 F F F	0.7999999999999972 0.0000000000000000 0.0000000000000000 F F F	0.7999999999999972 0.0000000000000000 0.0000000000000000 F F F
0.7999999999999972 0.2000000000000028 0.0000000000000000 F F F	0.7999999999999972 0.2000000000000028 0.0000000000000000 F F F	0.7999999999999972 0.2000000000000028 0.0000000000000000 F F F
0.7999999999999972 0.3999999999999986 0.0000000000000000 F F F	0.7999999999999972 0.3999999999999986 0.0000000000000000 F F F	0.7999999999999972 0.3999999999999986 0.0000000000000000 F F F
0.7999999999999972 0.6000000000000014 0.0000000000000000 F F F	0.7999999999999972 0.6000000000000014 0.0000000000000000 F F F	0.7999999999999972 0.6000000000000014 0.0000000000000000 F F F
0.7999999999999972 0.7999999999999972 0.0000000000000000 F F F	0.7999999999999972 0.7999999999999972 0.0000000000000000 F F F	0.7999999999999972 0.7999999999999972 0.0000000000000000 F F F
0.2761236519117876 0.9278198311943484 0.1327233204184499 T T T	0.2775545662328540 0.9228353890411592 0.1334078947563524 T T T	0.2756720025716634 0.9236044371451840 0.1325433991272673 T T T
0.4666644456031577 0.7333340047990546 0.1368027475005462 T T T	0.4666644456120537 0.7333340047990546 0.1368027474930713 T T T	0.4666644456187115 0.7333340047990546 0.1368027474874722 T T T
0.6666644456031534 0.5333340047990589 0.1368027475005462 T T T	0.6666644456120494 0.5333340047990589 0.1368027474930713 T T T	0.6666644456187072 0.5333340047990589 0.1368027474874722 T T T
0.8618793007888876 0.3358245339651861 0.1301661018757480 T T T	0.8766927727345930 0.3510556565244288 0.1331325732304071 T T T	0.8616335111293771 0.3387953289014125 0.1301854000956983 T T T
0.2663797728966663 0.7316795867060356 0.1327196913245399 T T T	0.2648729348551537 0.7254605100439412 0.1330072391554513 T T T	0.2636079854157238 0.7255383613534767 0.1342986623259290 T T T
0.4736200859267635 0.9371571497732512 0.1343784281192424 T T T	0.4759588283108025 0.9367565272308778 0.1347475904347542 T T T	0.4740902423861987 0.9364123258434672 0.1342768273690592 T T T
0.4666644456031577 0.5333340047990589 0.1368027475005462 T T T	0.4666644456120537 0.5333340047990589 0.1368027474930713 T T T	0.4666644456187115 0.5333340047990589 0.1368027474874722 T T T
0.6666644456031534 0.7333340047990546 0.1368027475005462 T T T	0.6666644456120494 0.7333340047990546 0.1368027474930713 T T T	0.6666644456187072 0.7333340047990546 0.1368027474874722 T T T
0.8608241542170086 0.5325903660902195 0.1341499003968534 T T T	0.8609806949994184 0.5361187899933771 0.1352096266764411 T T T	0.8707147581596307 0.5436122895935079 0.1321671123730218 T T T
0.6654347127332602 0.3354039902319105 0.1327416466842824 T T T	0.6698400569350571 0.3354758913144252 0.1322387688301181 T T T	0.6558759480025973 0.3314017074198691 0.1301412957587763 T T T
0.2580862789839746 0.5286763141635877 0.1315618197512199 T T T	0.2580313665427508 0.5250541138666838 0.1304666244418921 T T T	0.2603223349461531 0.5278145711324801 0.1322920356853330 T T T
0.4679771093216881 0.3380139687097364 0.1360827147416684 T T T	0.4706105520417468 0.3379498967701501 0.1361263964225759 T T T	0.4649121274882424 0.3375837405774271 0.1360433389855049 T T T
0.2706183363415890 0.3338187958699417 0.1285086055384095 T T T	0.2709567471192422 0.3299415327180311 0.1270319169408700 T T T	0.2681237888407114 0.3342116179046336 0.1288233898106571 T T T
0.6712073760819974 0.9398540937026104 0.1319600995990565 T T T	0.6730893532603237 0.9392623732461318 0.1316530609758570 T T T	0.6722276497998285 0.9404802202511902 0.1320909311300175 T T T
0.8618965699893191 0.7326850497112367 0.1354466818458297 T T T	0.8625463761826326 0.7344449829522599 0.1351547786709444 T T T	0.8618263001920708 0.7347778079194587 0.1351792693694261 T T T
0.8655734476262216 0.9315875326188950 0.1286367334122483 T T T	0.8678127304746265 0.9334117848891652 0.1280401907030244 T T T	0.8662293243010195 0.9328247647625436 0.1285594946636733 T T T
0.3486553999039206 0.8521218609935657 0.2661965272922538 T T T	0.3524658750469685 0.8422290569421838 0.2654845081550919 T T T	0.3500751541295435 0.8500544612779126 0.2655051150450474 T T T
0.5333313179997745 0.6666662410030355 0.2736054950010853 T T T	0.5333313180131043 0.6666662410030355 0.2736054949861355 T T T	0.5333313180231087 0.6666662410030355 0.2736054949749445 T T T
0.3218100110889323 0.6490240545779831 0.2677532909866263 T T T	0.3163405704100769 0.6354528395215183 0.2669589426113659 T T T	0.3254414398205130 0.6472590874364180 0.2665086584928069 T T T
0.5254655764253243 0.4677396782528800 0.2740325986736656 T T T	0.5192818215193151 0.4658614035200854 0.2741301145886325 T T T	0.5378148315121434 0.4682748703729230 0.2714752004652617 T T T
0.3274513065068088 0.4576459046113668 0.2606002569263587 T T T	0.3145102151645132 0.4459560153075279 0.2588234654275842 T T T	0.3342537775339245 0.4557777477332600 0.2607159580251618 T T T
0.5492292418175059 0.8720095173576039 0.2660660684965180 T T T	0.5570203490055783 0.8745887183917127 0.2666081550271251 T T T	0.5528228697605423 0.8750010902902119 0.2663701473952645 T T T
0.7297906026016708 0.6663681231691140 0.2722053247047242 T T T	0.7299583364776656 0.6645144376746327 0.2705412604259360 T T T	0.7303288189889073 0.6654770881367615 0.2720240561334797 T T T
0.7422064188342645 0.8689042619638054 0.2596890268135197 T T T	0.7482071725670791 0.8687095265079027 0.2593829218781218 T T T	0.7447796791025745 0.8690325285354404 0.2599969937825141 T T T
0.7163182955247975 0.4655079784876336 0.2650235004820248 T T T	0.7115645522470216 0.4615233739177669 0.2651769516457628 T T T	0.7320878058340610 0.4672836818856664 0.2647205284984930 T T T
0.4258278598243272 0.7722354840751254 0.3945604981629520 T T T	0.4301472165473186 0.7594956688857349 0.3900674571405083 T T T	0.4274317016763530 0.7743306999848463 0.3934279115376569 T T T
0.3952751333454403 0.5669082310967621 0.3951111664309668 T T T	0.3979607366568985 0.5521108543447543 0.3919903912994750 T T T	0.3897252700263955 0.5677033790421474 0.3941047115411169 T T T
0.6320991481058638 0.8077789158225698 0.3937363048430915 T T T	0.6396697650242156 0.8064107883244881 0.3939088857384734 T T T	0.6335321588178053 0.8084807887630396 0.3935434452485014 T T T
0.5997808732466814 0.6021882778262472 0.4056268948864898 T T T	0.6061485052120664 0.6001554652616332 0.4041791024556071 T T T	0.5957476555672943 0.6000402952215469 0.4047642382871205 T T T
0.5100882853944445 0.6922356688235982 0.5122924292638521 T T T	0.5118291308632839 0.6829481273736832 0.5087997574788410 T T T	0.5101260044072872 0.6906780731063347 0.5108601492892423 T T T
0.0044120868068559 0.3336351872336645 0.3859114966795592 T T T	0.0344981605311752 0.3716649705200400 0.3768977972915147 T T T	0.9395633605272764 0.3595203960751032 0.3579163096785259 T T T
0.9755782631689500 0.3739633513463833 0.3030003725794074 T T T	0.0410840671689165 0.4205863157855325 0.2852852448429355 T T T	0.9711799291867348 0.4661808479222064 0.2988164674761227 T T T
0.9132259228097204 0.1727097351220463 0.3385035666934232 T T T	0.9514395137327949 0.2162987871934216 0.3230013068056860 T T T	0.8403444230093896 0.2132014517223341 0.2885433844531506 T T T
0.0087904226688885 0.4712156363937767 0.2947161736355418 T T T	0.0566984090896981 0.5132862484437531 0.2818366431018423 T T T	0.9870831449397820 0.5445342485872543 0.3387246249737805 T T T
0.9719418128100494 0.3675794954906367 0.4402202063617894 T T T	0.9661350278320844 0.3860750059098446 0.4113100781732704 T T T	0.8590990469942943 0.3385062581709525 0.3971160737122768 T T T
0.1041213090469381 0.3835304643949608 0.3919455029446464 T T T	0.1195337104765877 0.4221502106707982 0.4128366275776555 T T T	0.0147609949917773 0.3834348237471397 0.4032952411090668 T T T
0.2202473190227991 0.4992604912413686 0.2407693439332338 T T T	0.1801799245763534 0.4674033825845426 0.2564780213834455 T T T	0.0548272655391017 0.4904722369423264 0.2625338783565703 T T T
0.9575643503406566 0.2092846050273173 0.3925855583352295 T T T	1.0002619952624454 0.2489367332468920 0.3760657999682075 T T T	0.9224743084262468 0.2561018844129628 0.3106071311647022 T T T
0.9150496520480716 0.3003870233457944 0.2452865406171894 T T T	0.9682359748813840 0.3414592732540453 0.2300538329162405 T T T	0.8752071997042600 0.4376734745491226 0.2375647728796312 T T T

Table S14. Atomic coordinates of IS, TS, and FS of Reaction 9 listed in Table S1.

Reaction 9: $*CH_2OH–CH_2O + H^+ + e^- \rightarrow CH_2OH–CH_3 + *O$ on Cu-DAN via surface *H transfer

IS

```
Cu  C  H  O
  1.00000000000000
  12.8585004805000001  0.0000000000000000  0.0000000000000000
  -6.4291002405000004  11.1357880710000003  0.0000000000000000
  0.0000000000000000  0.0000000000000000  15.2994003295999992
Cu  C  H  O
55  2  6  2
Selective dynamics
Direct
  0.0000000000000000  0.0000000000000000  0.0000000000000000 F F F
  0.0000000000000000  0.2000000000000028  0.0000000000000000 F F F
  0.0000000000000000  0.3999999999999986  0.0000000000000000 F F F
  0.0000000000000000  0.6000000000000014  0.0000000000000000 F F F
  0.0000000000000000  0.7999999999999972  0.0000000000000000 F F F
  0.2000000000000028  0.0000000000000000  0.0000000000000000 F F F
  0.2000000000000028  0.2000000000000028  0.0000000000000000 F F F
  0.2000000000000028  0.3999999999999986  0.0000000000000000 F F F
  0.2000000000000028  0.6000000000000014  0.0000000000000000 F F F
  0.2000000000000028  0.7999999999999972  0.0000000000000000 F F F
  0.3999999999999986  0.0000000000000000  0.0000000000000000 F F F
  0.3999999999999986  0.2000000000000028  0.0000000000000000 F F F
  0.3999999999999986  0.3999999999999986  0.0000000000000000 F F F
  0.3999999999999986  0.6000000000000014  0.0000000000000000 F F F
  0.3999999999999986  0.7999999999999972  0.0000000000000000 F F F
  0.6000000000000014  0.0000000000000000  0.0000000000000000 F F F
  0.6000000000000014  0.2000000000000028  0.0000000000000000 F F F
  0.6000000000000014  0.3999999999999986  0.0000000000000000 F F F
  0.6000000000000014  0.6000000000000014  0.0000000000000000 F F F
  0.6000000000000014  0.7999999999999972  0.0000000000000000 F F F
  0.7999999999999972  0.0000000000000000  0.0000000000000000 F F F
  0.7999999999999972  0.2000000000000028  0.0000000000000000 F F F
  0.7999999999999972  0.3999999999999986  0.0000000000000000 F F F
  0.7999999999999972  0.6000000000000014  0.0000000000000000 F F F
  0.7999999999999972  0.7999999999999972  0.0000000000000000 F F F
  0.2744366393428607  0.9257087469915096  0.1320078141484228 T T T
  0.4666644456031577  0.7333340047990546  0.1368027475005462 T T T
  0.6666644456031534  0.5333340047990589  0.1368027475005462 T T T
  0.8582300728614861  0.3360903791083737  0.1301203999752996 T T T
  0.2645728620329414  0.7283313265232871  0.1337608814578433 T T T
  0.4720536622179538  0.9361657049862592  0.1336345825205599 T T T
  0.4666644456031577  0.5333340047990589  0.1368027475005462 T T T
  0.6666644456031534  0.7333340047990546  0.1368027475005462 T T T
  0.8694984896194502  0.5402863602253558  0.1309881900118947 T T T
  0.6515068779775574  0.3263117390964216  0.1323757735330723 T T T
  0.2612344478221794  0.5301123289773310  0.1330959665057073 T T T
  0.4617934197909201  0.3336845062759491  0.1414202176278924 T T T
  0.2690660870073077  0.3367342231745249  0.1293377488107725 T T T
  0.6706932676348192  0.9398997095808946  0.1327215686746877 T T T
  0.8623396892252876  0.7333617280805175  0.1348631903820176 T T T
  0.8640994974575894  0.9305563927154455  0.1294337164755407 T T T
  0.3460413838375380  0.8540087823658735  0.2655669290765321 T T T
  0.5333313179997745  0.6666662410030355  0.2736054950010853 T T T
  0.3275412939960604  0.6525984182022815  0.2667653491838564 T T T
  0.5423787208358840  0.4746344586181326  0.2740828674486979 T T T
  0.3372378832549888  0.4603540068482996  0.2622890860764242 T T T
  0.5466501736429041  0.8742825279349778  0.2658945953359768 T T T
  0.7323516432707685  0.6690013203240841  0.2718395771708835 T T T
  0.7396158168906993  0.8679148603763950  0.2611620657756177 T T T
  0.7433739395951015  0.4794614628248251  0.2658602189489243 T T T
  0.4202688435855573  0.7808286206635827  0.3959484328214158 T T T
  0.3932716519111416  0.5774732192184687  0.3950767601211713 T T T
  0.6250131702393575  0.8146463828715327  0.3937545023568692 T T T
  0.6007007757116833  0.6125485886106884  0.4068252135890286 T T T
  0.5087930054950230  0.6995622755835873  0.5133824253362546 T T T
  0.9437351048668278  0.3584963890562798  0.3610434801424955 T T T
  0.9732371361510824  0.4604827819219401  0.2976746049752621 T T T
  0.8441195367581296  0.2126925864953398  0.2916542506525999 T T T
  0.5228901283418337  0.2580227114329180  0.1837016055680720 T T T
  0.9943041349470728  0.5424839246110167  0.3347514371056869 T T T
  0.8660726739853926  0.3416884233845601  0.4020985768816490 T T T
  0.0215803039641006  0.3856958202172941  0.4044674846096891 T T T
  0.0536924231353299  0.4794671559045026  0.2590905723363494 T T T
  0.9233889503498970  0.2507998946860574  0.3188118177430584 T T T
  0.8739342189570407  0.4314289074205752  0.2392251491843614 T T T
```

TS

```
Cu  C  H  O
  1.00000000000000
  12.8585004805000001  0.0000000000000000  0.0000000000000000
  -6.4291002405000004  11.1357880710000003  0.0000000000000000
  0.0000000000000000  0.0000000000000000  15.2994003295999992
Cu  C  H  O
55   2   6   2
Selective dynamics
Direct
  0.0000000000000000  0.0000000000000000  0.0000000000000000  F  F  F
  0.0000000000000000  0.2000000000000028  0.0000000000000000  F  F  F
  0.0000000000000000  0.3999999999999986  0.0000000000000000  F  F  F
  0.0000000000000000  0.6000000000000014  0.0000000000000000  F  F  F
  0.0000000000000000  0.7999999999999972  0.0000000000000000  F  F  F
  0.2000000000000028  0.0000000000000000  0.0000000000000000  F  F  F
  0.2000000000000028  0.2000000000000028  0.0000000000000000  F  F  F
  0.2000000000000028  0.3999999999999986  0.0000000000000000  F  F  F
  0.2000000000000028  0.6000000000000014  0.0000000000000000  F  F  F
  0.2000000000000028  0.7999999999999972  0.0000000000000000  F  F  F
  0.3999999999999986  0.0000000000000000  0.0000000000000000  F  F  F
  0.3999999999999986  0.2000000000000028  0.0000000000000000  F  F  F
  0.3999999999999986  0.3999999999999986  0.0000000000000000  F  F  F
  0.3999999999999986  0.6000000000000014  0.0000000000000000  F  F  F
  0.3999999999999986  0.7999999999999972  0.0000000000000000  F  F  F
  0.6000000000000014  0.0000000000000000  0.0000000000000000  F  F  F
  0.6000000000000014  0.2000000000000028  0.0000000000000000  F  F  F
  0.6000000000000014  0.3999999999999986  0.0000000000000000  F  F  F
  0.6000000000000014  0.6000000000000014  0.0000000000000000  F  F  F
  0.6000000000000014  0.7999999999999972  0.0000000000000000  F  F  F
  0.7999999999999972  0.0000000000000000  0.0000000000000000  F  F  F
  0.7999999999999972  0.2000000000000028  0.0000000000000000  F  F  F
  0.7999999999999972  0.3999999999999986  0.0000000000000000  F  F  F
  0.7999999999999972  0.6000000000000014  0.0000000000000000  F  F  F
  0.7999999999999972  0.7999999999999972  0.0000000000000000  F  F  F
  0.2757634503209447  0.9245244526846964  0.1288281706361248  T  T  T
  0.4666644456000029  0.7333340047999997  0.1368027474999991  T  T  T
  0.6666644455999986  0.5333340047999968  0.1368027474999991  T  T  T
  0.8699991874356819  0.3308794638099289  0.1221901425777182  T  T  T
  0.2643744093620456  0.7266162447145027  0.1306106128498220  T  T  T
  0.4732084320997345  0.9347574707548032  0.1307713339885007  T  T  T
  0.4666644456000029  0.5333340047999968  0.1368027474999991  T  T  T
  0.6666644455999986  0.7333340047999997  0.1368027474999991  T  T  T
  0.8646574515717305  0.5338546104564008  0.1300109292731049  T  T  T
  0.6601977965049272  0.3313730691987100  0.1308974591233575  T  T  T
  0.2609895979947373  0.5275077939893738  0.1291156382887337  T  T  T
  0.4666214349245646  0.3375582070534683  0.1305974812837548  T  T  T
  0.2681334454766005  0.3340860664619403  0.1254219706502784  T  T  T
  0.6727551964288868  0.9385887390036880  0.1293371853950349  T  T  T
  0.8617806718055266  0.7311866356730008  0.1314128111247255  T  T  T
  0.8657276713809261  0.9308600764553980  0.1254077219649425  T  T  T
  0.3502503196643935  0.8487757465695928  0.2631042664245554  T  T  T
  0.5333313180000019  0.6666662410000015  0.2736054949999982  T  T  T
  0.3213998514535610  0.6448498094384922  0.2649010357572685  T  T  T
  0.5330364037059596  0.4678189402460641  0.2702545826234437  T  T  T
  0.3309150050279610  0.4550486986664333  0.2588456980323056  T  T  T
  0.5552382164472981  0.8781285715214756  0.2650464411121765  T  T  T
  0.7300792026479522  0.6614231789655369  0.2728590654772008  T  T  T
  0.7454261024262036  0.8663441787437700  0.2593594161507362  T  T  T
  0.7472654048985021  0.4666630220487892  0.2816881954318803  T  T  T
  0.4282743903988565  0.7712515438693422  0.3902541592434516  T  T  T
  0.3888080913863791  0.5642681794322457  0.3928604119077492  T  T  T
  0.6353609794893820  0.8100774766063001  0.3931605367392632  T  T  T
  0.5967451160429079  0.6012096049537409  0.4043938605837564  T  T  T
  0.5101395985739186  0.6888790484745534  0.5088779024639463  T  T  T
  0.7838693930862850  0.3325954469970556  0.4240954507872289  T  T  T
  0.8158618298675049  0.4611048592293185  0.4029060709765740  T  T  T
  0.8060502644819487  0.3013098322486332  0.3018625196682708  T  T  T
  0.7234173148610431  0.5219481698259989  0.3608139391638432  T  T  T
  0.8023652414301948  0.5033192708825294  0.4610442024463445  T  T  T
  0.6880586006467192  0.2779769592043795  0.4432742875685116  T  T  T
  0.8362860872408505  0.3384826412653301  0.4835010252729776  T  T  T
  0.9091918510904911  0.5172088926765318  0.3802856430883975  T  T  T
  0.8144029416534045  0.2726808632188301  0.3601990738603820  T  T  T
  0.7997731351685335  0.3782489437909359  0.2103970927387993  T  T  T
```

FS

```
Cu  C  H  O
  1.00000000000000
  12.8585004805000001  0.0000000000000000  0.0000000000000000
  -6.4291002405000004  11.1357880710000003  0.0000000000000000
  0.0000000000000000  0.0000000000000000  15.2994003295999992
Cu  C  H  O
55  2  6  2
Selective dynamics
Direct
  0.0000000000000000  0.0000000000000000  0.0000000000000000 F F F
  0.0000000000000000  0.2000000000000028  0.0000000000000000 F F F
  0.0000000000000000  0.3999999999999986  0.0000000000000000 F F F
  0.0000000000000000  0.6000000000000014  0.0000000000000000 F F F
  0.0000000000000000  0.7999999999999972  0.0000000000000000 F F F
  0.2000000000000028  0.0000000000000000  0.0000000000000000 F F F
  0.2000000000000028  0.2000000000000028  0.0000000000000000 F F F
  0.2000000000000028  0.3999999999999986  0.0000000000000000 F F F
  0.2000000000000028  0.6000000000000014  0.0000000000000000 F F F
  0.2000000000000028  0.7999999999999972  0.0000000000000000 F F F
  0.3999999999999986  0.0000000000000000  0.0000000000000000 F F F
  0.3999999999999986  0.2000000000000028  0.0000000000000000 F F F
  0.3999999999999986  0.3999999999999986  0.0000000000000000 F F F
  0.3999999999999986  0.6000000000000014  0.0000000000000000 F F F
  0.3999999999999986  0.7999999999999972  0.0000000000000000 F F F
  0.6000000000000014  0.0000000000000000  0.0000000000000000 F F F
  0.6000000000000014  0.2000000000000028  0.0000000000000000 F F F
  0.6000000000000014  0.3999999999999986  0.0000000000000000 F F F
  0.6000000000000014  0.6000000000000014  0.0000000000000000 F F F
  0.6000000000000014  0.7999999999999972  0.0000000000000000 F F F
  0.7999999999999972  0.0000000000000000  0.0000000000000000 F F F
  0.7999999999999972  0.2000000000000028  0.0000000000000000 F F F
  0.7999999999999972  0.3999999999999986  0.0000000000000000 F F F
  0.7999999999999972  0.6000000000000014  0.0000000000000000 F F F
  0.7999999999999972  0.7999999999999972  0.0000000000000000 F F F
  0.2775379839499419  0.9233865078508138  0.1330000619368655 T T T
  0.4666644456031577  0.7333340047990546  0.1368027475005462 T T T
  0.6666644456031534  0.5333340047990589  0.1368027475005462 T T T
  0.8635210701849614  0.3330009994386119  0.1266835065177122 T T T
  0.2632422750338969  0.7246379508662281  0.1344226265725328 T T T
  0.4759155207694119  0.9370793779099720  0.1344045719731181 T T T
  0.4666644456031577  0.5333340047990589  0.1368027475005462 T T T
  0.6666644456031534  0.7333340047990546  0.1368027475005462 T T T
  0.8668362197742544  0.5371993444258868  0.1339337158697246 T T T
  0.6598085826974381  0.3316231819964185  0.1338496263362990 T T T
  0.2593984124177633  0.5270074817571568  0.1315513592063421 T T T
  0.4665002368143330  0.3378635497964166  0.1350331963905259 T T T
  0.2673618317007911  0.3328800053235535  0.1279915233198526 T T T
  0.6738789839808194  0.9416251210983836  0.1316126413220370 T T T
  0.8623759090583770  0.7326146733952241  0.1347455268055910 T T T
  0.8672362485932450  0.9324144496009684  0.1277953729220505 T T T
  0.3526993334296759  0.8482798978495998  0.2653854651469106 T T T
  0.5333313179997745  0.6666662410030355  0.2736054950010853 T T T
  0.3236377489369072  0.6439590768945407  0.2664515945783989 T T T
  0.5326518646139627  0.4687878444001297  0.2719851620886350 T T T
  0.3301298694514506  0.4530429090371841  0.2590454927636089 T T T
  0.5573861922523281  0.8773018085774157  0.2662989776086173 T T T
  0.7313751546650880  0.6671224664744804  0.2714970606287361 T T T
  0.7473462455059243  0.8694813364933890  0.2586910712662308 T T T
  0.7268350721723220  0.4726826566520535  0.2716280289181344 T T T
  0.4323719429785368  0.7696326923892878  0.3915437291312154 T T T
  0.3924180194187462  0.5618607314836839  0.3928584244097783 T T T
  0.6404394387499245  0.8103129319755967  0.3937299039933084 T T T
  0.6019562611125300  0.5997232266545831  0.4032875728441005 T T T
  0.5144142786038537  0.6857572384206611  0.5087350916187374 T T T
  0.8906491994459922  0.1901027322087174  0.4655302985440436 T T T
  0.9278020482013972  0.2930992493231870  0.4016511442491482 T T T
  0.8039089215643681  0.0661825062119152  0.3754601816164663 T T T
  0.8542926470777089  0.2761246685192176  0.3564165031508429 T T T
  0.9518179057257986  0.3769641410225075  0.4367449254538479 T T T
  0.8152070368931169  0.1795056405609469  0.5067382812544913 T T T
  0.9652459557473704  0.2073862549541124  0.5096412478398552 T T T
  0.0058541626334373  0.3078856210735467  0.3626182614955770 T T T
  0.8593249286250998  0.0780870076710802  0.4232642250024882 T T T
  0.8029121628213129  0.3917128436138595  0.2164642966482045 T T T
```

Table S15. Atomic coordinates of IS, TS, and FS of Reaction 10 listed in Table S1.

Reaction 10: *CH_2OH–CH_2O+ H^++e^-→*CH_2OH–CH_2OH on Cu-DAN via H-shuttling

IS

```
Cu C H O
 1.00000000000000
 12.8585004805000001 0.0000000000000000 0.0000000000000000
 -6.4291002405000004 11.1357880710000003 0.0000000000000000
 0.0000000000000000 0.0000000000000000 15.2994003295999992
Cu C H O
55 2 8 3
Selective dynamics
Direct
 0.0000000000000000 0.0000000000000000 0.0000000000000000 F F F
 0.0000000000000000 0.2000000000000028 0.0000000000000000 F F F
 0.0000000000000000 0.3999999999999986 0.0000000000000000 F F F
 0.0000000000000000 0.6000000000000014 0.0000000000000000 F F F
 0.0000000000000000 0.7999999999999972 0.0000000000000000 F F F
 0.2000000000000028 0.0000000000000000 0.0000000000000000 F F F
 0.2000000000000028 0.2000000000000028 0.0000000000000000 F F F
 0.2000000000000028 0.3999999999999986 0.0000000000000000 F F F
 0.2000000000000028 0.6000000000000014 0.0000000000000000 F F F
 0.2000000000000028 0.7999999999999972 0.0000000000000000 F F F
 0.3999999999999986 0.0000000000000000 0.0000000000000000 F F F
 0.3999999999999986 0.2000000000000028 0.0000000000000000 F F F
 0.3999999999999986 0.3999999999999986 0.0000000000000000 F F F
 0.3999999999999986 0.6000000000000014 0.0000000000000000 F F F
 0.3999999999999986 0.7999999999999972 0.0000000000000000 F F F
 0.6000000000000014 0.0000000000000000 0.0000000000000000 F F F
 0.6000000000000014 0.2000000000000028 0.0000000000000000 F F F
 0.6000000000000014 0.3999999999999986 0.0000000000000000 F F F
 0.6000000000000014 0.6000000000000014 0.0000000000000000 F F F
 0.6000000000000014 0.7999999999999972 0.0000000000000000 F F F
 0.7999999999999972 0.0000000000000000 0.0000000000000000 F F F
 0.7999999999999972 0.2000000000000028 0.0000000000000000 F F F
 0.7999999999999972 0.3999999999999986 0.0000000000000000 F F F
 0.7999999999999972 0.6000000000000014 0.0000000000000000 F F F
 0.7999999999999972 0.7999999999999972 0.0000000000000000 F F F
 0.2752303944766232 0.9263359656921808 0.1301153490706918 T T T
 0.4666644456031577 0.7333340047990546 0.1368027475005462 T T T
 0.6666644456031534 0.5333340047990589 0.1368027475005462 T T T
 0.8545743282796847 0.3333469135437325 0.1303676321528229 T T T
 0.2682822594659300 0.7326501781691810 0.1365413568420741 T T T
 0.4741982126658023 0.9344040380957902 0.1324216036979791 T T T
 0.4666644456031577 0.5333340047990589 0.1368027475005462 T T T
 0.6666644456031534 0.7333340047990546 0.1368027475005462 T T T
 0.8652753651327058 0.5374113739578591 0.1310545448074178 T T T
 0.6468236552719199 0.3226605321043938 0.1330326819259660 T T T
 0.2615767367688727 0.5322797571850801 0.1331806255079303 T T T
 0.4593307370485910 0.3313632145452218 0.1410923288964443 T T T
 0.2685007721007158 0.3375545346299040 0.1294594853564495 T T T
 0.6742603994476639 0.9414422909065310 0.1322231547749926 T T T
 0.8620180535622982 0.7335269980148659 0.1351635213331809 T T T
 0.8670747331774857 0.9319923705029102 0.1281968981917073 T T T
 0.3583994239358973 0.8682957235667612 0.2696506011080131 T T T
 0.5333313179997745 0.6666662410030355 0.2736054950010853 T T T
 0.3312343802005254 0.6602757896688132 0.2677177967682297 T T T
 0.5381304692258596 0.4712494010898286 0.2721503981390175 T T T
 0.3343107668208665 0.4627393952531190 0.2611976837157955 T T T
 0.5546520437929250 0.8733061963723471 0.2658291367501808 T T T
 0.7300267009014604 0.6664417888046522 0.2722637038914287 T T T
 0.7475123838235719 0.8698902985245337 0.2594737651793512 T T T
 0.7352320553837279 0.4732795317225998 0.2666569122243488 T T T
 0.4352855329002502 0.7815409247424704 0.3969439205760735 T T T
 0.3915240303180250 0.5741833506062360 0.3951781868567371 T T T
 0.6377237258320959 0.8089669945556817 0.3928927745611411 T T T
 0.5965327898524324 0.6003238797633668 0.4053270733379669 T T T
 0.5162015444752682 0.6942264241604830 0.5117621525768934 T T T
 0.9402511749769648 0.3528227385312556 0.3564602049625749 T T T
 0.9662361837111290 0.4588663669934679 0.2984018110398110 T T T
 0.8475504677588446 0.2077069938537733 0.2839572390554156 T T T
 0.4405736584575494 0.1075672672590930 0.2762459783700361 T T T
 0.5013302300109148 0.0681642421409062 0.3465346431851808 T T T
 0.9811818533647404 0.5359868164690105 0.3392387244268551 T T T
 0.8595226364499621 0.3274178060902465 0.3967153320490787 T T T
 0.0169653532968931 0.3800841891648614 0.4010093553690381 T T T
 0.0490835787479282 0.4858107595994356 0.2603808129015729 T T T
 0.5132122262278708 0.2462520026865973 0.1799104008608621 T T T
 0.9283129749168602 0.2519666002883865 0.3085105608505694 T T T
 0.8677634096021904 0.4292733607225207 0.2391139970147138 T T T
 0.4227370165011188 0.0488325778579263 0.3234360195027663 T T T
```

TS

```
Cu C H O
 1.00000000000000
 12.8585004805000001 0.0000000000000000 0.0000000000000000
 -6.4291002405000004 11.1357880710000003 0.0000000000000000
 0.0000000000000000 0.0000000000000000 15.2994003295999992
 Cu C H O
 55 2 8 3
Selective dynamics
Direct
 0.0000000000000000 0.0000000000000000 0.0000000000000000 F F F
 0.0000000000000000 0.2000000000000028 0.0000000000000000 F F F
 0.0000000000000000 0.3999999999999986 0.0000000000000000 F F F
 0.0000000000000000 0.6000000000000014 0.0000000000000000 F F F
 0.0000000000000000 0.7999999999999972 0.0000000000000000 F F F
 0.2000000000000028 0.0000000000000000 0.0000000000000000 F F F
 0.2000000000000028 0.2000000000000028 0.0000000000000000 F F F
 0.2000000000000028 0.3999999999999986 0.0000000000000000 F F F
 0.2000000000000028 0.6000000000000014 0.0000000000000000 F F F
 0.2000000000000028 0.7999999999999972 0.0000000000000000 F F F
 0.3999999999999986 0.0000000000000000 0.0000000000000000 F F F
 0.3999999999999986 0.2000000000000028 0.0000000000000000 F F F
 0.3999999999999986 0.3999999999999986 0.0000000000000000 F F F
 0.3999999999999986 0.6000000000000014 0.0000000000000000 F F F
 0.3999999999999986 0.7999999999999972 0.0000000000000000 F F F
 0.6000000000000014 0.0000000000000000 0.0000000000000000 F F F
 0.6000000000000014 0.2000000000000028 0.0000000000000000 F F F
 0.6000000000000014 0.3999999999999986 0.0000000000000000 F F F
 0.6000000000000014 0.6000000000000014 0.0000000000000000 F F F
 0.6000000000000014 0.7999999999999972 0.0000000000000000 F F F
 0.7999999999999972 0.0000000000000000 0.0000000000000000 F F F
 0.7999999999999972 0.2000000000000028 0.0000000000000000 F F F
 0.7999999999999972 0.3999999999999986 0.0000000000000000 F F F
 0.7999999999999972 0.6000000000000014 0.0000000000000000 F F F
 0.7999999999999972 0.7999999999999972 0.0000000000000000 F F F
 0.2773777964800411 0.9222419000728921 0.1293458503576518 T T T
 0.4666644456031577 0.7333340047990546 0.1368027475005462 T T T
 0.6666644456031534 0.5333340047990589 0.1368027475005462 T T T
 0.8637138154419725 0.3380461849621274 0.1262996806660348 T T T
 0.2645416613321549 0.7239179526846687 0.1302513061034207 T T T
 0.4764097341321640 0.9359008310851725 0.1301166398179229 T T T
 0.4666644456031577 0.5333340047990589 0.1368027475005462 T T T
 0.6666644456031534 0.7333340047990546 0.1368027475005462 T T T
 0.8650346473115492 0.5359204975854532 0.1280487488118068 T T T
 0.6652200117175091 0.3363545180009431 0.1293041673734719 T T T
 0.2613212571552079 0.5250722925616592 0.1283069267876680 T T T
 0.4708258788601080 0.3300341624782520 0.1351409532446227 T T T
 0.2715803769007810 0.3320064415748434 0.1243567766023799 T T T
 0.6752795544548595 0.9395305877564261 0.1282591319630198 T T T
 0.8627357308360559 0.7318419356090969 0.1317985608571546 T T T
 0.8680797473818902 0.9329144361736561 0.1241730886407527 T T T
 0.3566140402145501 0.8449530615765880 0.2617040095968727 T T T
 0.5333313179997745 0.6666662410030355 0.2736054950010853 T T T
 0.3205890174985280 0.6384585720073458 0.2644456619464646 T T T
 0.5302636785046347 0.4692520020003283 0.2705822088605070 T T T
 0.3257554028970018 0.4477925254308346 0.2575516498985327 T T T
 0.5631839418831645 0.8805155965533321 0.2644922942229344 T T T
 0.7309640500603066 0.6711538979765426 0.2712991959118077 T T T
 0.7538939370031433 0.8772968316911793 0.2571862802156853 T T T
 0.7356705739654935 0.4792900835473503 0.2664868728935046 T T T
 0.4375590913078621 0.7647088796034128 0.3861725432188284 T T T
 0.3892168193151520 0.5538277736451751 0.3916222599171517 T T T
 0.6480441789575122 0.8088727335652416 0.3910585252117545 T T T
 0.5999064690210686 0.5971914140098252 0.4024869032842752 T T T
 0.5150263581283289 0.6849909463281264 0.5046154227002237 T T T
 0.8708558154559405 0.2576812270260797 0.3213179548175524 T T T
 0.8634408772340881 0.3682426544562322 0.3445785854286114 T T T
 0.8317461937053998 0.1555640667532708 0.2121671926938962 T T T
 0.4914548330241680 0.1846058993286511 0.3168970539045927 T T T
 0.6392564225935943 0.2538385456259750 0.3033094585225822 T T T
 0.8848537203476134 0.3851768604704701 0.4151603776782871 T T T
 0.7956108307997359 0.1764110771985987 0.3503468003917269 T T T
 0.9564369836343320 0.2687584638307717 0.3461405843881472 T T T
 0.9359048375200832 0.4459097719490311 0.3090810815352459 T T T
 0.5426795367931384 0.2544777252444228 0.2024667569351873 T T T
 0.8680175878745318 0.2413364249115310 0.2270267113117903 T T T
 0.7500603303498855 0.3572910259190437 0.3272026744583758 T T T
 0.5525660537017550 0.1895124752980919 0.2762482679011309 T T T
```

FS

```
Cu C H O
 1.00000000000000
 12.8585004805000001 0.0000000000000000 0.0000000000000000
 -6.4291002405000004 11.1357880710000003 0.0000000000000000
 0.0000000000000000 0.0000000000000000 15.2994003295999992
Cu C H O
55 2 8 3
Selective dynamics
Direct
 0.0000000000000000 0.0000000000000000 0.0000000000000000 F F F
 0.0000000000000000 0.2000000000000028 0.0000000000000000 F F F
 0.0000000000000000 0.3999999999999986 0.0000000000000000 F F F
 0.0000000000000000 0.6000000000000014 0.0000000000000000 F F F
 0.0000000000000000 0.7999999999999972 0.0000000000000000 F F F
 0.2000000000000028 0.0000000000000000 0.0000000000000000 F F F
 0.2000000000000028 0.2000000000000028 0.0000000000000000 F F F
 0.2000000000000028 0.3999999999999986 0.0000000000000000 F F F
 0.2000000000000028 0.6000000000000014 0.0000000000000000 F F F
 0.2000000000000028 0.7999999999999972 0.0000000000000000 F F F
 0.3999999999999986 0.0000000000000000 0.0000000000000000 F F F
 0.3999999999999986 0.2000000000000028 0.0000000000000000 F F F
 0.3999999999999986 0.3999999999999986 0.0000000000000000 F F F
 0.3999999999999986 0.6000000000000014 0.0000000000000000 F F F
 0.3999999999999986 0.7999999999999972 0.0000000000000000 F F F
 0.6000000000000014 0.0000000000000000 0.0000000000000000 F F F
 0.6000000000000014 0.2000000000000028 0.0000000000000000 F F F
 0.6000000000000014 0.3999999999999986 0.0000000000000000 F F F
 0.6000000000000014 0.6000000000000014 0.0000000000000000 F F F
 0.6000000000000014 0.7999999999999972 0.0000000000000000 F F F
 0.7999999999999972 0.0000000000000000 0.0000000000000000 F F F
 0.7999999999999972 0.2000000000000028 0.0000000000000000 F F F
 0.7999999999999972 0.3999999999999986 0.0000000000000000 F F F
 0.7999999999999972 0.6000000000000014 0.0000000000000000 F F F
 0.7999999999999972 0.7999999999999972 0.0000000000000000 F F F
 0.2759200299787876 0.9237234135863662 0.1326659582988172 T T T
 0.4666644456031577 0.7333340047990546 0.1368027475005462 T T T
 0.6666644456031534 0.5333340047990589 0.1368027475005462 T T T
 0.8624082871316023 0.3354230880915400 0.1296293874128203 T T T
 0.2622555927190314 0.7245190177616372 0.1341731224836595 T T T
 0.4726257254064523 0.9334384292329968 0.1332492052806979 T T T
 0.4666644456031577 0.5333340047990589 0.1368027475005462 T T T
 0.6666644456031534 0.7333340047990546 0.1368027475005462 T T T
 0.8613511523785923 0.5312156608517815 0.1318314290783674 T T T
 0.6614081027256387 0.3321227116115528 0.1348302264771014 T T T
 0.2600081300344605 0.5275384653678538 0.1320789038816905 T T T
 0.4658312704038034 0.3393666128196543 0.1349495812176396 T T T
 0.2671345222920231 0.3336736514574323 0.1284582196606954 T T T
 0.6728793269995632 0.9420197180467555 0.1321468571368237 T T T
 0.8623440171154060 0.7322032525434422 0.1353685968221877 T T T
 0.8659133563215219 0.9318939831614278 0.1284191460872809 T T T
 0.3501268520815790 0.8490386884167482 0.2651576807727232 T T T
 0.5333313179997745 0.6666662410030355 0.2736054950010853 T T T
 0.3241117691433762 0.6462335691312294 0.2663561796971608 T T T
 0.5336604134534190 0.4709126728081804 0.2717573253828087 T T T
 0.3301307442738687 0.4549161551878202 0.2600124029058704 T T T
 0.5547422624850828 0.8789831669624028 0.2662890200760519 T T T
 0.7312741753667077 0.6699429101270725 0.2712809275539816 T T T
 0.7453194929820134 0.8730471049172727 0.2594849626950212 T T T
 0.7333760134957218 0.4765351386600111 0.2677314259457009 T T T
 0.4278690608905713 0.7735058326859123 0.3923619329479785 T T T
 0.3911625425827952 0.5663393977845369 0.3933770166303957 T T T
 0.6352615952061270 0.8116516373021087 0.3927408525834914 T T T
 0.5993763646378202 0.6043466659175595 0.4044834906419001 T T T
 0.5108621961129370 0.6921141789467464 0.5094231152647895 T T T
 0.8772061104039978 0.2534144349519242 0.3273134831691970 T T T
 0.8763711632131590 0.3667945445079074 0.3518268463032506 T T T
 0.8241783580821355 0.1439673071467856 0.2218719340645386 T T T
 0.5044001717301544 0.1617568494817377 0.2558831038830605 T T T
 0.7010236184524472 0.2804269355245902 0.3162948498460212 T T T
 0.9062182713674524 0.3890328685846666 0.4201795015942699 T T T
 0.8084361784058248 0.1755492342972929 0.3642690535642501 T T T
 0.9664512537958490 0.2656681097047314 0.3432726390864139 T T T
 0.9400946665706572 0.4418252599973292 0.3101013188247346 T T T
 0.5792123609620253 0.1021191932979448 0.2270065735209265 T T T
 0.8540840999020844 0.2297852560970170 0.2346235353335083 T T T
 0.7594451747036228 0.3569232995960964 0.3455689837084643 T T T
 0.5871350755119658 0.1802067575216419 0.2456669763093481 T T T
```

Supplementary References

1 T. Cheng, H. Xiao, W. A. Goddard III, *Proc. Natl. Acad. Sci. U.S.A.*, 2017, **114**, 8, 1795–1800.

2 Y. Lum, T. Cheng, W. A. Goddard III, J. W. Ager, *J. Am. Chem. Soc.*, 2018, **140**, 9337–9340.

3 F. Calle-Vallejo, M. T. M. Koper, *Angew Chem. Int. Ed.*, 2013, **52**, 28, 7282–7285.

4 G. Henkelman, H. J. Jónsson, *Chem. Phys.*, 2000, **113**, 9978-9985.

5 X. Nie, M. R. Esopi, M. J. Janik, A. Asthagiri, *Angew. Chem., Int. Ed.*, 2013, **52**, 2459–2462.

6 W. Luo, X. Nie, M. J. Janik, A. Asthagiri, *ACS Catal.,* 2016, **6**, 219–229.

7 J. A. Garza, T. A. Bell, M. Head-Gardon, *ACS Catal.*, 2018, **8**, 1490–1499.
